# The Link between Type 2 Diabetes Mellitus and the Polymorphisms of Glutathione-Metabolizing Genes Suggests a New Hypothesis Explaining Disease Initiation and Progression

**DOI:** 10.3390/life11090886

**Published:** 2021-08-28

**Authors:** Iuliia Azarova, Elena Klyosova, Alexey Polonikov

**Affiliations:** 1 Department of Biological Chemistry, Kursk State Medical University, 3 Karl Marx Street, 305041 Kursk, Russia; azzzzar@yandex.ru; 2 Laboratory of Biochemical Genetics and Metabolomics, Research Institute for Genetic and Molecular Epidemiology, Kursk State Medical University, 18 Yamskaya St., 305041 Kursk, Russia; ecless@yandex.ru; 3 Laboratory of Statistical Genetics and Bioinformatics, Research Institute for Genetic and Molecular Epidemiology, Kursk State Medical University, 18 Yamskaya St., 305041 Kursk, Russia; 4 Department of Biology, Medical Genetics and Ecology, Kursk State Medical University, 3 Karl Marx Street, 305041 Kursk, Russia

**Keywords:** type 2 diabetes, etiology, gamma-glutamyl transferase, glutathione synthase, glutathione, insulin folding, unfolded protein response

## Abstract

The present study investigated whether type 2 diabetes (T2D) is associated with polymorphisms of genes encoding glutathione-metabolizing enzymes such as glutathione synthetase (*GSS*) and gamma-glutamyl transferase 7 (*GGT7*). A total of 3198 unrelated Russian subjects including 1572 T2D patients and 1626 healthy subjects were enrolled. Single nucleotide polymorphisms (SNPs) of the *GSS* and *GGT7* genes were genotyped using the MassArray-4 system. We found that the *GSS* and *GGT7* gene polymorphisms alone and in combinations are associated with T2D risk regardless of sex, age, and body mass index, as well as correlated with plasma glutathione, hydrogen peroxide, and fasting blood glucose levels. Polymorphisms of *GSS* (rs13041792) and *GGT7* (rs6119534 and rs11546155) genes were associated with the tissue-specific expression of genes involved in unfolded protein response and the regulation of proteostasis. Transcriptome-wide association analysis has shown that the pancreatic expression of some of these genes such as *EDEM2*, *MYH7B*, *MAP1LC3A*, and *CPNE1* is linked to the genetic risk of T2D. A comprehensive analysis of the data allowed proposing a new hypothesis for the etiology of type 2 diabetes that endogenous glutathione deficiency might be a key condition responsible for the impaired folding of proinsulin which triggered an unfolded protein response, ultimately leading to beta-cell apoptosis and disease development.

## 1. Introduction

Diabetes is a major global health threat. The International Diabetes Federation estimated that 1 in 11 adults aged 20–79 years (463 million adults) was affected by diabetes globally in 2019 [1]. In the Russian Federation, approximately 8.1 million people suffer from diabetes, and insulin non-dependent form or type 2 diabetes is responsible for a majority of these cases [2]. Type 2 diabetes (T2D) is a chronic metabolic disorder characterized by persistent hyperglycemia, insulin resistance, and a relative lack of insulin. Chronic hyperglycemia and hyperglycemia-driven micro- and macrovascular complications make T2D the 9th cause of mortality worldwide [3].

Type 2 diabetes is a multifactorial highly heterogeneous polygenic disease determined by tight interactions between various genetic, epigenetic, and environmental factors [4,5,6]. Numerous studies have identified hundreds of genetic polymorphisms associated with T2D susceptibility and disease-related pathological conditions such as β-cell function and mass, insulin action/resistance, glucagon secretion/action, incretin secretion/action, and fat distribution [7,8]. It has been argued that decreased peripheral glucose uptake combined with augmented endogenous glucose production resulting in insulin resistance is associated with the diminished β-cell function and decreased secretion of insulin by the pancreas in type 2 diabetes [9]. In addition, abnormal redox homeostasis and oxidative stress have been found to play a crucial role in the disease’s pathogenesis [10,11]. In particular, numerous studies have shown that the increased production of free radicals, poor antioxidant defense, and associated oxidative stress contribute to type 2 diabetes, beta-cell apoptosis and insulin resistance and disease complications [10,12,13,14,15,16].

Although the advent of genomic, transcriptomic, proteomic, and metabolomic technologies facilitated rapid advances in characterizing the pathological processes underlying type 2 diabetes in the molecular details, nevertheless, the exact etiological factors and mechanisms responsible for the initiation of apoptosis leading to a progressive loss of β-cell function remain elusive. The lack of progress in our understanding of the true triggering factors of type 2 diabetes seems to be explained by a poor understanding of the causal relationships between the key disease processes such as hyperglycemia, insulin secretion, insulin resistance, beta-cell apoptosis, oxidative stress, inflammation, and endoplasmic reticulum stress, thereby complicating the pathophysiological interpretation of newly discovered biomarkers in the context of their associative or causal link to the disease’s pathogenesis. This problem justifies the need to move beyond conventional insights into the primary mechanisms of type 2 diabetes and propose novel disease hypotheses that should be tested in relation to known pathological disorders underlying diabetes.

It is well known that the balance between oxidants and antioxidants is largely determined by the ability of cells to synthesize glutathione, a tripeptide (gamma-glutamyl-cysteinyl-glycine) with powerful antioxidant activity [17]. Glutathione has critical biological and molecular functions in the cell, including the regulation of gene expression, DNA and protein synthesis, protein folding, cell proliferation and apoptosis, signal transduction, the regeneration of vitamins C and E, control of cytokine production, antiviral defense, and the regulation of immune response [18,19,20]. Glutathione exists in reduced (GSH) and oxidized or glutathione disulfide (GSSG) forms, representing the major redox couple in cells. GSH functions as an antioxidant scavenging reactive oxygen species (ROS) protecting the cell from oxidative damage, and GSSG possesses a strong regulatory potential concerning posttranslational protein modulation (e.g., glutathionylation) and the epigenetic control of gene expression [21,22,23]. The deficiency of endogenous glutathione contributes to oxidative stress and plays a key role in both the aging and pathogenesis of many cardiovascular and degenerative diseases as well as type 2 diabetes [24]. Although abnormal glutathione metabolism plays an obvious role in the development of type 2 diabetes and its complications, existing explanations regarding the specific role of glutathione deficiency in T2D pathogenesis are limited by a hypothesis that decreased antioxidant defense and increased oxidative stress both contribute to the development and progression of this disease.

The present study was designed to hypothesize that the endogenous deficiency of glutathione, particularly in pancreatic beta-cells, is the initial factor triggering a chain of sequential pathological conditions, not limited by oxidative stress, that ultimately lead to the development of type 2 diabetes. Our hypothesis is based on research findings obtained over the last sixty years which show that glutathione deficiency seems to occur initially in type 2 diabetes due to impaired biosynthesis and/or the depletion of GSH [25,26,27,28,29,30]. This hypothesis is supported by our [31,32,33,34,35] and some other [36,37,38] studies which have revealed that single nucleotide polymorphisms (SNPs) at genes encoding glutathione-metabolizing enzymes such as catalytic and modifier subunits of glutamate cysteine ligase, gamma-glutamyl cyclotransferase, glutathione S-transferases (mu, pi, and theta classes), gamma-glutamyltransferase-6 and glutathione peroxidase-1 substantially contribute to type 2 diabetes’ susceptibility. This means that glutathione metabolism represents an attractive pathway for genetic research into type 2 diabetes because these genes are directly involved in biosynthesis, catabolism, and the utilization of reduced glutathione and thereby may influence disease risk. The purpose of this study was to investigate whether genetic susceptibility to type 2 diabetes is associated with common SNPs at genes encoding glutathione synthetase (GSS), the second enzyme in glutathione biosynthesis, and gamma-glutamyl transferase 7 (GGT7), an enzyme involved in glutathione recycling. We also performed for the first time a comprehensive bioinformatics analysis in an attempt to unravel the molecular mechanisms by which the glutathione-metabolizing enzymes contribute to the pathogenesis of type 2 diabetes.

## 2. Materials and Methods

### 2.1. Study Participants and Diagnosis of Type 2 Diabetes

The study protocol was approved by the Ethical Review Committee of Kursk State Medical University. Written informed consent was obtained from each participant before enrollment in the study. A total of 3198 unrelated Russian individuals were recruited for the study, including 1572 T2D patients and 1626 age- and sex-matched healthy subjects. All study subjects were mainly from the Kursk region, Central Russia.

Patients with T2D were admitted to the Division of Endocrinology of Kursk Emergency Hospital from November 2016 to October 2019. T2D was diagnosed based on WHO criteria [39] such as fasting blood glucose (FBG) levels ≥ 7.0 mmol/L or random blood glucose levels ≥ 11.1 mmol/L and/or glycated hemoglobin HbA1c levels ≥ 6.5%. The criteria for inclusion in the group of patients were: (1) a physician-verified disease confirmed by clinical, laboratory, and instrumental investigations; (2) age over 35 years; and (3) written informed consent to participate in the study. Criteria for the exclusion of patients in the case group were the following: (1) below 35 years of age; (2) the absence of written informed consent to participate in the study; and (3) clinical conditions such as a pronounced degree of decompensation of T2D or coma, immune-mediated or idiopathic type 1 diabetes, gestational diabetes, MODY types of diabetes, diseases of the exocrine pancreas such as pancreatitis, pancreatic trauma or pancreatectomy, pancreatic tumors, hereditary diseases affecting the pancreas, and any other endocrine disorders. The control group included healthy volunteers who visited Kursk Blood Transfusion Station within the same time frame as well as healthy individuals recruited in our previous studies [31,40,41,42]. The criteria for the inclusion of persons in the control group were: (1) age over 35 years; (2) normal blood glucose levels according to the WHO criteria; (3) the absence of chronic diseases; and (4) written informed consent. The exclusion criteria from the control group were the following: (1) age under 35 years; (2) a history of hyperglycemia, the presence of chronic diseases; and (3) a lack of written informed consent. Subjects of the control group had normal FBG levels and 75 g oral glucose tolerance test results.

### 2.2. Blood Specimen Collection and Processing

Fasting venous blood samples of study participants were collected by venipuncture in two separate tubes: the first one with EDTA solution (0.5 M) for genomic DNA extraction and genotyping and the second one with lithium heparin for biochemical investigations of redox homeostasis. Blood samples for molecular genetic analysis were frozen and maintained at −80 °C until processed. Blood samples with lithium heparin were centrifuged at 3500 rpm according to the manufacturer’s instructions (Cell Biolabs Inc., San Diego, CA, USA; Abcam, Waltham, MA, USA) immediately after blood sampling. Then, plasma samples were aliquoted and stored at −80 °C until further use. For total GSH determination, plasma was immediately deproteinized with 5% metaphosphoric acid. Genomic DNA was purified from thawed blood samples by phenol–chloroform extraction or by spin-column QIAamp Blood mini kit with the use of the robotic workstation QiaCube (QIAGEN). Samples were measured spectrophotometrically and DNA concentration was adjusted to a value of 10 ng/mL and stored at −80 °C until genotyping.

### 2.3. SNP Selection and Molecular Genetic Analysis

The SNPinfo bioinformatics tools such as GenePipe and FuncPred [43] were used for functional SNP selection (https://snpinfo.niehs.nih.gov, date of access 18 September 2017), as described previously [32]. Briefly, SNP selection was based on the default settings of GenePipe (genotype data from HapMap, CEU population, minor allele frequency cutoff value 0.05) with value 2 as a minimum number of SNPs tagged by a tag SNP. FuncPred was utilized to assess the functional significance of SNPs (regulatory potential, transcription factor binding site, etc.). Initially, the following SNPs such as rs13041792, rs6087653, rs6088660 of *GSS* and rs11546155, rs6119536, and rs6119534 of *GGT7* were selected by the GenePipe tool. The FuncPred tool confirmed the functional significance for all the SNPs except for rs6087653 and rs6119534. Then, SNP rs6087653 was substituted for rs1801310, a functionally significant polymorphism of the glutathione synthetase gene known from the literature [44]. SNP rs6119534 of *GGT7* was saved in the SNP set as an alternative to the rs6087653 polymorphism, which was excluded by Assay Design Suite (Agena Bioscience, USA), as it did not meet the conditions for co-genotyping in a single multiplex panel. Thus, the final multiplexing panel included three SNPs (rs13041792, rs1801310, and rs6088660) of the *GSS* gene and two SNPs (rs11546155 and rs6119534) of the *GGT7* gene. The genotyping of the polymorphisms was performed by MALDI-TOF mass spectrometry iPLEX technology with the MassArray System (Agena Bioscience Inc, San Diego, CA, USA). Primer sequences used for genotyping are available upon request. To ensure quality control, 10% of the samples were randomly selected for repeat genotyping that was performed blindly to the case–control status, and the repeatability test resulted in a 100% concordance rate.

### 2.4. Biochemical Analysis

Plasma ROS levels were assessed in 426 T2D patients and 153 healthy volunteers, whereas GSH levels were assessed in 258 patients and 137 controls were recruited during the final phase of this study. The total GSH levels were determined by colorimetric assay using the OxiSelect^TM^ Total Glutathione (GSSG/GSH) Assay Kit (Cell Biolabs, USA). The ROS levels were quantified by a fluorometric assay using the OxiSelect^TM^ In Vitro ROS/RNS Assay Kit (Cell Biolabs, USA). The assay employs a proprietary quenched fluorogenic probe, dichlorodihydrofluorescin DiOxyQ (DCFH-DiOxyQ), which is a specific ROS/RNS probe. It was first primed with a quench removal reagent and subsequently stabilized in the highly reactive DCFH form. In this reactive state, ROS and RNS can react with DCFH, which is rapidly oxidized to the highly fluorescent 2′,7′-dichlorodihydrofluorescein (DCF). The standard curve of H_2_O_2_ was used to quantify the levels of free radicals such as ROS and RNS in plasma samples. Absorbance was measured on a microplate reader Varioscan Flash (Thermo Fisher Scientific, Waltham, MA, USA) at 405 nm and fluorescence at 480 nm excitation/530 nm emission.

### 2.5. Statistical Power Calculation

Statistical power for the study was estimated using the genetic association study power calculator [45] available online at http://csg.sph.umich.edu/abecasis/gas_power_calculator/, date of access 12 May 2020. Association analysis between the polymorphisms of *GSS* and *GGT7* genes and the risk of T2D could detect the genotype relative risk (GRR) of 1.26–1.51 assuming 85% power and a 5% type I error (α = 0.05) on the sample size of 1572 cases and 1626 controls.

### 2.6. Association of GSS and GGT7 Gene Polymorphisms with Type 2 Diabetes

**Figure 1:**
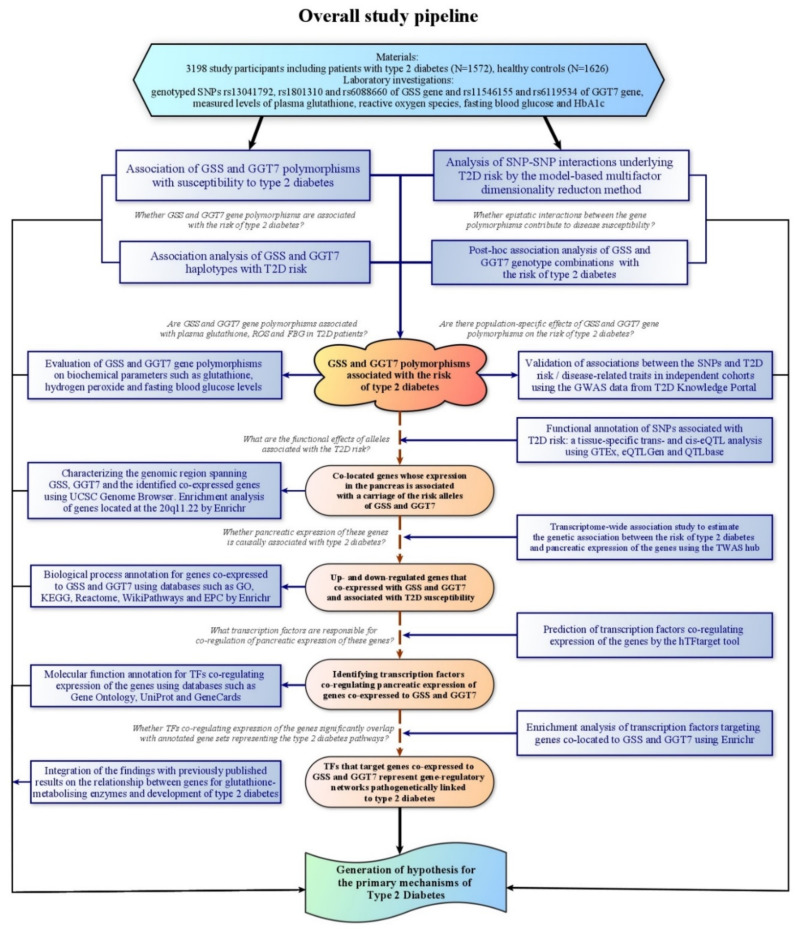
Overall study pipeline. The upper part of the figure shows the statistical genetic analysis for the association between type 2 diabetes risk and *GSS* and *GGT7* gene polymorphisms. The lower part of the figure shows a comprehensive bioinformatics analysis of the observed associations. Methods of statistical/bioinformatics analyses are indicated in blue rectangles. The key findings are designated by ellipses. Hypotheses tested at each stage of data analysis are indicated in Italics. Abbreviations used: KEGG, Kyoto Encyclopedia of Genes and Genomes; GO, Gene Ontology; EPC, Elsevier Pathway Collection. Figure is created using software yEd Graph Editor.

The overall study pipeline is summarized in Figure 1. Allele and genotype frequencies in cases and controls were counted and compared by the chi-square test with the values predicted by the assumption of the Hardy–Weinberg equilibrium. The association of SNPs with the risk of T2D was evaluated by multiple logistic regression analysis with the calculation of odds ratios (ORs) and 95% confidence intervals (95%CI) adjusted for covariates such as age, sex, and body mass index (BMI) using the SNPStats software [46] available online at https://snpstats.net, date of access 12 May 2020 Haplotypes and linkage disequilibrium (LD, D, and D’ values) between the polymorphisms were estimated and analyzed using the SNPStats software.

### 2.7. A Replication of the Associations in Independent Populations

It was argued that the replication of genetic association in independent populations provides additional evidence that the association is not due to uncontrolled bias [47]. However, inter-population differences in allele frequencies and linkage disequilibrium between SNPs may be significant factors influencing a replication of marker-disease association in a distinct individual population [48]. In the present study, we conducted a replication study in several populations at once and analyzed the frequencies of alleles and linkage disequilibrium between the markers in each and the populations to assess whether there are population-specific effects of *GSS* and *GGT7* gene polymorphisms on the risk of type 2 diabetes. The genotype–phenotype datasets from genome-wide association studies (GWAS) deposited in the T2D Knowledge Portal (https://t2d.hugeamp.org, date of access 31 January 2021) were utilized to validate associations between the SNPs with type 2 diabetes or disease-related traits in independent cohorts. Interethnic differences in the association between type 2 diabetes and functionally significant SNPs of *GSS* and *GGT7* genes were evaluated in two independent samples of diabetics from the UK Biobank (http://geneatlas.roslin.ed.ac.uk, date of access 14 January 2021). Allele frequencies and linkage disequilibrium (D’) between SNPs across the HapMap populations were evaluated with the LDpair tool available through the website https://ldlink.nci.nih.gov/?tab=ldpair (date of access 20 January 2021).

### 2.8. Analysis of the Contribution of SNP–SNP Interactions to T2D Susceptibility

It has been advised that if an SNP has not been replicated in an independent population, this polymorphism may be checked for interactions with other genetic variants [48]. This motivated us to perform an analysis of SNP–SNP interactions to evaluate whether epistatic interactions between the gene polymorphisms contributed to T2D susceptibility. Two-order SNP–SNP interactions associated with the risk of type 2 diabetes were evaluated with the model-based multifactor dimensionality reduction (MB-MDR) method proposed by Calle and co-workers [49], a dimension reduction method for exploring gene–gene and gene–environment interactions, and implemented in mbmdr package for R [50]. Statistical significance for each mbmdr model was assessed through permutation tests. For each significant SNP–SNP interaction model, the post hoc comparisons of genotype combinations between the groups were performed using the chi-square test. A false discovery rate (FDR) was calculated for all SNP–disease associations to control for multiple testing. FDR calculations were performed using the FDR calculator available online at http://www.sdmproject.com/utilities/?show=FDR (date of access 28 May 2021).

### 2.9. Impact of GSS and GGT7 Gene Polymorphisms on Biochemical Parameters

Biochemical parameters were analyzed for normality with the Kolmogorov–Smirnov test. Age and body mass index (BMI) showed normal distribution, expressed as the mean (M) with standard deviation (SD) and compared between the groups using the Student’s *t*-test. Non-normally distributed traits such as glycated hemoglobin (HbA1c), fasting blood glucose (FGB), total cholesterol, high- and low-density lipoproteins, triacylglycerol, hydrogen peroxide, and total glutathione were expressed as the median (Me) with the first and third quartiles (Q1–Q3). The impact of *GSS* and *GGT7* gene polymorphisms on biochemical parameters such as glutathione, hydrogen peroxide, fasting blood glucose levels, and glycated hemoglobin in diabetics was evaluated by the Kruskal–Wallis test using STATISTICA for Windows 10.0 package (StatSoft, Tulsa, OK, USA).

The upper part of the figure shows the statistical genetic analysis for the association between type 2 diabetes risk and *GSS* and *GGT7* gene polymorphisms. The lower part of the figure shows a comprehensive bioinformatics analysis of the observed associations. Methods of statistical/bioinformatics analyses are indicated in blue rectangles. The key findings are designated by ellipses. Hypotheses tested at each stage of data analysis are indicated in Italics.

The abbreviations used include: KEGG, Kyoto Encyclopedia of Genes and Genomes; GO, Gene Ontology; EPC, Elsevier Pathway Collection. Figure was created using the software yEd Graph Editor.

### 2.10. Functional Annotation of GSS and GGT7 Gene Polymorphisms

*GSS* and *GGT7* gene polymorphisms significantly associated with the risk of type 2 diabetes were a subject for comprehensive bioinformatics analysis (Figure 1). The functional annotation of SNPs associated with T2D risk included a tissue-specific *trans*- and *cis*-eQTL analysis. In particular, bioinformatics tools of the quantitative trait loci (QTL) databases such as the GTEx portal (https://gtexportal.org, date of access 11 November 2020) [51], eQTLGen Consortium (https://www.eqtlgen.org, date of access 12 November 2020) [52] and QTLbase (http://mulinlab.tmu.edu.cn/qtlbase/index.html, date of access 14 November 2020) [53] were used to evaluate whether the GSS and GGT7 gene polymorphisms represent significant QTLs correlating with a variation of molecular traits such as mRNA expression (eQTL), methylation (mQTL), histone modification (hQTL) and splicing events (sQTL). GTEx portal and QTLbase databases were used to identify various QTLs for the studied SNPs in a tissue-specific manner focusing on the pancreas and insulin-sensitive tissues such as skeletal muscle cells, visceral adipose tissue and the liver. The GTEx portal is a comprehensive public resource to study tissue-specific gene expression and regulation. QTLbase is a database curating and compiling genome-wide QTL summary statistics for multiple human molecular traits across over 70 tissues and cell types. The eQTLGen Consortium database, incorporating 37 datasets with a total of 31,684 individuals, was used to assess eQTLs for the studied SNPs in whole blood. Genes whose expression in the pancreas was found to be associated with a carriage of the risk alleles of GSS and GGT7 (i.e., genes co-expressed genes with the SNPs) were explored using the UCSC Genome Browser (genome.ucsc.edu/cgi-bin/hgTracks?db, date of access 20 May 2021) which was also used to characterize the genomic region spanning *GSS*, *GGT7* and co-located or co-expressed genes.

### 2.11. Transcriptome-Wide Association Analysis of Genes Co-Expressed with GSS and GGT7

A transcriptome-wide association study (TWAS) was done with the purpose to estimate the genetic association between the risk of type 2 diabetes and the pancreatic expression of the genes using the TWAS hub (http://twas-hub.org, date of access 9 June 2021). TWAS is a known integrative method for identifying the genes causally associated with phenotypes through the construction of expression prediction models for every gene using its cis-SNPs as predictors [54]. The TWAS hub, an integrative bioinformatics resource, was utilized to estimate the genetic association between the risk of type 2 diabetes and the expression of genes whose mRNA levels in various tissues are associated with the disease susceptibility loci observed in the present study. The TWAS hub interactive browser was developed to allow enabling the genetic association between gene expression and a complex phenotype using only GWAS summary-level data [55]. Gene-expression prediction models were built using the GWAS summary-level data implemented in the TWAS hub and transcriptome–genotype data from the GTEx [52], METSIM [56], NTR [57], and YFS [58] eQTL projects. Transcriptomic data on the major T2D-related tissues (pancreas, adipose tissue, skeletal muscle, and whole blood) available from public datasets [59,60,61] and The Neale lab were applied to estimate a predictive association model with a Z-score for each gene whose mRNA levels were related with SNPs of *GSS* (rs13041792) and *GGT7* (rs6119534 and rs11546155) genes. The Z-score shows the number of standard deviations that a value of gene expression is away from the mean of all the expression values for a gene in the same group. Probability Q-values for Z-scores were estimated with the Z-score Calculator (https://www.fourmilab.ch/rpkp/experiments/analysis/zCalc.html, date of access 11 June 2021). Thus, the TWAS analysis ultimately allows identifying the link between genes of interest and disease susceptibility.

### 2.12. In Silico Prediction of Transcription Factors Co-Regulating Expression of the Target Genes

Transcription factors co-regulating the target genes were predicted by hTFtarget (http://bioinfo.life.hust.edu.cn/hTFtarget/, date of access 14 June 2021), a comprehensive database for the investigation of TF-target regulations in humans [62]. The only transcription factors whose co-regulation effects have been confirmed by two or more independent datasets were selected to include in a gene set. Among them, TFs that are significantly expressed in the pancreas (FDR ≤ 0.05), as assessed by the Tissue Expression database (also called as “Jensen Tissues”), were used for further analysis. TFs expressed in the pancreas at excessively low levels (TPM value less than 0.003 at the GTEx portal) were excluded from the gene set. Molecular function annotation for the selected TFs (i.e., activator or repressor function) was done with the use of databases such as Gene Ontology, UniProt, and GeneCards. Protein–protein interaction (PPI) networks of transcription factors belonging to the T2D pathways were constructed by STRING, a database of known and predicted protein–protein interactions [63].

### 2.13. Gene Set Enrichment Analysis

Genes with the up- and downregulated expression and co-located to *GSS* and *GGT7* were annotated towards a biological process using databases such as Gene Ontology, KEGG, Reactome, WikiPathways, and Elsevier Pathway Collection by Enrichr [64], an interactive gene list enrichment analysis available online at https://maayanlab.cloud/Enrichr/ (date of access 7 May 2021). Enrichment analysis was also used to annotate genes spanning genomic cluster 20q11.22 as well as the transcription factors expressed in the pancreas towards biological processes and molecular functions. Finally, the enrichment analysis was performed to evaluate whether the query TFs significantly overlap with the annotated gene sets representing pathogenetically important pathways for type 2 diabetes.

## 3. Results

### 3.1. Demographic, Clinical and Laboratory Characteristics of the Study Participants

Demographic, clinical and laboratory characteristics of the study groups are shown in Table 1. The study groups were matched according to both sex and age. The majority of T2D patients (87.1%) were overweight or obese. Most T2D patients suffered from hypertension, and approximately one-third had coronary artery disease. The number of patients with a positive history of diabetes was greater in the case group than in the control group. The number of smokers was higher among healthy subjects than among diabetics. Plasma ROS levels were significantly higher in diabetics compared to the controls (*p* < 0.0001), whereas total glutathione concentration was lower in T2D patients than in healthy subjects (*p* = 0.037). Moreover, diabetic patients showed significantly increased levels of glycated hemoglobin, FBG, triacylglycerols, total cholesterol, and low-density lipoproteins as compared with healthy controls. Meanwhile, the levels of high-density lipoproteins were higher in healthy subjects than in T2D patients.

### 3.2. Association of GSS and GGT7 Gene Polymorphisms with the Risk of T2D

Genotype and allele frequencies for the studied SNPs are shown in Table 2. Genotype frequencies for all polymorphisms were in Hardy–Weinberg equilibrium in both cases and controls. As can be seen from Table 2, SNPs such as rs13041792 of *GSS*, rs11546155, and rs6119534 of *GGT7* showed associations with the risk of type 2 diabetes. In particular, genotypes G/A-A/A at rs13041792 were significantly associated with an increased risk of T2D (OR 1.21, 95CI 1.04–1.42, *p* = 0.046) and this association remained significant after making adjustments for age, sex and body mass index (Q = 0.06). Meanwhile, SNPs rs6119534 (OR 0.73, 95%CI 0.53–0.99) and rs11546155 (OR 0.42, 95%CI 0.22–0.80) of the *GGT7* gene showed significant associations with a decreased risk of T2D regardless of sex, age and BMI after adjustment for multiple testing by the FDR procedure. The frequency of minor allele rs6119534-T *GGT7* was higher in controls compared to patients (OR 0.85, 95CI 0.76–0.95, Q = 0.03).

### 3.3. Associations of GSS and GGT7 Haplotypes with Disease Susceptibility

The estimated haplotype frequencies of *GSS* and *GGT7* genes in T2D patients and healthy controls are given in Table 3. Four and three common haplotypes were identified for *GSS* and *GGT7* genes, respectively. Haplotype rs1801310G–rs6088660C–rs13041792A of the *GSS* gene showed a significant association with an increased risk of T2D (OR 1.26, 95% CI 1.08–1.47). Haplotypes rs6119534T–rs11546155G (OR 0.80, 95% CI 0.68–0.95) and rs6119534C–rs11546155A (OR 0.76, 95% CI 0.61–0.94) of the *GGT7* gene were associated with a decreased T2D risk. The observed associations remained significant after adjustments for multiple tests (Q ≤ 0.05). Supplementary Table S1 shows linkage disequilibrium values between the polymorphisms of *GSS* and *GGT7* genes. SNPs of the GSS gene were found in a strong (D’ > 0.8) negative linkage disequilibrium to each other (*p* < 0.0001), meaning that the reference alleles such as rs13041792-G, rs1801310-G, and rs6088660-C are more likely to be associated with the alternative alleles rs6088660-T rs13041792-A and rs1801310-A of *GSS*. Polymorphisms rs6119534 and rs11546155 of the *GGT7* gene were also in negative linkage disequilibrium with each other (D’ = 0.8228, *p* < 0.0001). Moreover, the SNPs of the *GSS* gene were in significant linkage disequilibrium to the SNPs of the *GGT7* gene. The D’values between pairs of the studied SNPs across populations from the 1000 Genomes Project are shown in Supplementary Table S2.

### 3.4. Epistatic Interactions between the Polymorphisms and Susceptibility to Type 2 Diabetes

The MB-MDR method was applied to evaluate two-order SNP–SNP interaction models determining genetic susceptibility to type 2 diabetes. The significance level (*p*_perm_) for each mbmdr model was assessed by permutations. Table 4 shows seven statistically significant two-order SNP–SNP interaction *mbmdr*-models associated with the risk of type 2 diabetes. The strongest epistatic interactions (*p*_perm_ < 0.001) were observed between SNP rs6119534 of the *GGT7* gene and polymorphisms rs13041792, rs1801310, and rs6088660 of the GSS gene, as well as between rs6119534 and rs11546155 of *GGT7*. Then, pairwise genotype combinations were compared between the study groups using the chi-square test for all seven mbmdr-models associated with disease risk. Table 5 lists eighteen genotype combinations significantly associated with the risk of type 2 diabetes after controlling for the false-discovery rate (Q ≤ 0.05). As can be seen from Table 5, the presence of the rs6119534-C/T *GGT7* genotype possessed a protective effect against the risk of type 2 diabetes by eliminating the negative effects of seven genotype combinations such as G1, G4, G5, G7, G8, G11, and G12 on the disease risk. However, genotype rs13041792-G/A *GSS* was found to eliminate the protective effects of the rs6119534-C/T *GGT7* genotype against T2D risk. In addition, we found that genotype rs11546155-A/A *GGT7* also possesses a protective effect against type 2 diabetes by eliminating the negative effects of *GSS* rs6088660-C/T, *GSS* rs1801310-G/G, and *GSS* rs13041792-G/G genotypes (i.e., G15, G16, and G18 in Table 5) on the disease risk. These results demonstrate that the polymorphisms of *GGT7* and GSS genes are in tight epistatic interactions with each other, and the observed antagonism between some SNPs (especially between rs6119534 *GGT7* and rs13041792 *GSS*) determines the likelihood of disease development.

### 3.5. Influence of GSS and GGT7 Gene Polymorphisms on Biochemical Parameters

Table 6 summarizes the impact of the studied polymorphisms on biochemical parameters such as redox homeostasis (hydrogen peroxide and total glutathione levels) and fasting blood glucose (FBG) in both entire and sex-stratified groups (Kruskal–Wallis test). Polymorphisms rs13041792 (*p* = 0.026) and rs6088660 (*p* = 0.007) of *GSS* were found to be associated with increased and decreased levels of FBG in diabetic males, respectively. An SNP rs6088660 of *GSS* showed association with decreased plasma levels of hydrogen peroxide in diabetic females (*p* = 0.039). Furthermore, SNP rs1801310 of *GSS* was associated with decreased levels of total glutathione in the plasma of T2D females (*p* = 0.039). However, the above genotype–phenotype associations did not survive after adjustment for multiple tests (Q > 0.05). Polymorphism rs6119534 of the *GGT7* gene showed association with increased levels of H_2_O_2_ in both sexes, but the association was stronger in females than in males after controlling the false-discovery rate. In particular, a carriage of the rs6119534-T allele of *GGT7* correlated with higher plasma levels of hydrogen peroxide in T2D patients, especially in females (Q = 0.02). In addition, the rs6119534-T allele of *GGT7* was associated with higher plasma levels of fasting blood glucose (*p* = 0.035), whereas genotype rs11546155-G/A of *GGT7* was associated with higher levels of hydrogen peroxide (*p* = 0.046), but these associations were only seen in diabetic females and did not remain significant after controlling the FDR (Q = 0.18). The carriers for the rs11546155-G/A genotype had higher glutathione levels in plasma than the carriers for other genotypes at this locus (*p* = 0.023).

**Figure 2:**
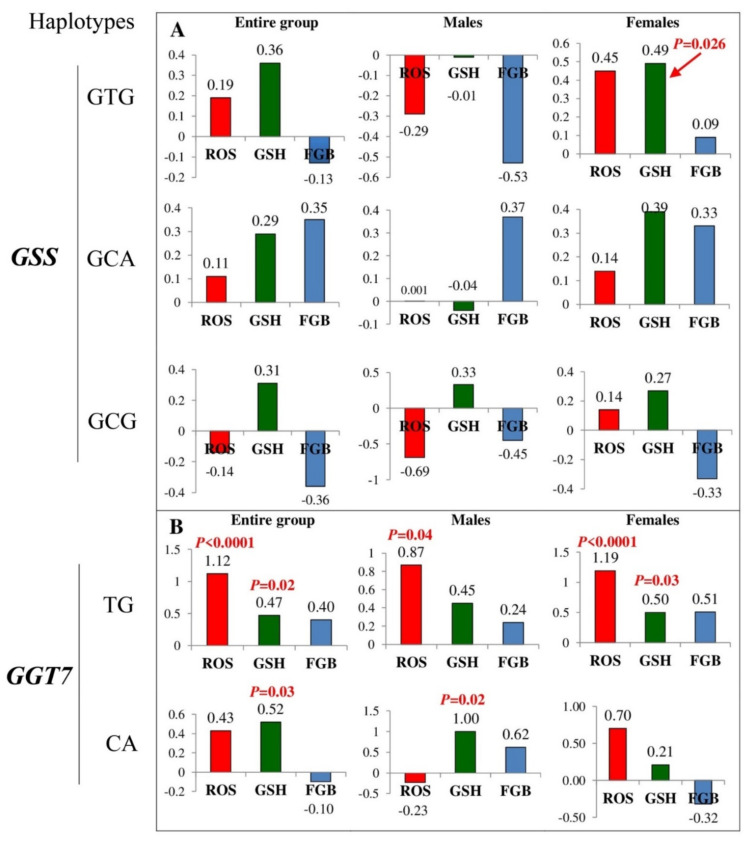
Relationship between the biochemical parameters and *GSS* and *GGT7* haplotypes in T2D patients: (**A**) haplotypes of the *GSS* gene (rs1801310, rs6088660 and rs13041792) in entire and sex-stratified groups; (**B**) haplotypes of the *GGT7* gene (rs6119534 and rs11546155) in entire and sex-stratified groups. The histograms show deviations of biochemical parameters from the median values (in µmol/L) in the assessed haplotypes according to the reference haplotypes such as the ACG of *GSS* and CG of *GGT7*. ROS, reactive oxygen species (hydrogen peroxide); GSH, total glutathione; FBG, fasting blood glucose.

Associations of *GSS* and *GGT7* haplotypes with plasma levels of hydrogen peroxide, glutathione, and fasting blood glucose are shown in Figure 2. A significant association between the CTG haplotype (rs1801310–rs6088660–rs13041792) of *GSS* and the increased levels of glutathione was exclusively observed in diabetic females (*p* = 0.026). In contrast, two common haplotypes of *GGT7* were significantly associated with the levels of both hydrogen peroxide and glutathione in patients with type 2 diabetes. A haplotype TG of *GGT7* was associated with increased levels of ROS (*p* < 0.0001) in both sexes but more strongly in females than males. Increased levels of glutathione were associated with haplotypes TG (*p* = 0.03) and CA (*p* = 0.02) in females and males, respectively. No significant associations were revealed between fasting blood glucose and haplotypes of the *GSS* and *GGT7* genes.

### 3.6. Validation for the Observed SNP-Disease Associations in Independent Populations

Replication of original association between SNP and disease phenotype in independent populations has become the gold standard for assessing the statistical results of genetic association studies [48]. In keeping with this approach, we performed a replication study for the observed SNP associations with type 2 diabetes and diabetes-related phenotypes in independent populations. We utilized population-specific whole-genome genotype datasets obtained from a large number of patients integrated into the T2D Knowledge Portal with the purpose of analyzing a relationship between the studied SNPs and type 2 diabetes as well as diabetes-related traits. Table 7 summarizes the results of the validation study for associations of SNPs with the T2D risk and disease-related phenotypes.

As can be seen from Table 7, the association of the SNP rs11546155 of the *GGT7* gene with a decreased risk of type 2 diabetes observed in our population has been successfully replicated in a large cohort of ethnically diverse individuals (*n =* 1,277,880, *p* = 0.018). In addition, this polymorphism was found to be associated with decreased levels of fasting plasma glucose in both diabetics and non-diabetics (*n* = 16,076, *p* = 0.016), as well as with decreased body mass index (*n* = 2,814,100, *p* = 0.027). In contrast with the above observations, SNP rs11546155 of *GGT7* also showed associations with increased waist–hip ratio adjusted by BMI (*n* = 1,834,510, *p* = 0.0009), the risk of chronic kidney disease (*n* = 300, *p* = 0.004), and neuropathy (*n* = 2344, *p* = 0.025) in type 2 diabetics, and increased levels of glycated hemoglobin (HbA1c) adjusted by BMI (*n* = 7267, *p* = 0.05). However, no associations of rs13041792 of *GSS* and rs6119534 *GGT7* with T2D susceptibility observed in our population were replicated in the studied population cohorts. Nonetheless, polymorphisms rs13041792 of *GSS* (*n =* 3,167,810, *p* = 0.0005) and rs6119534 of *GGT7* (*n* = 1,335,110, *p* = 0.0002) showed significant associations with an increased body mass index, as a diabetes-related phenotype, in two distinct cohorts of patients, respectively. Interestingly, a carriage of allele rs1801310 g of *GSS* in females and allele rs11546155-A of *GGT7* in males that are related to the increased levels of total glutathione in our population showed the link to a decreased fasting plasma glucose concentration in both diabetics and non-diabetics in a large cohort of individuals (*n* = 16,076).

To evaluate whether there exist interethnic differences in the association between type 2 diabetes and *GSS* and *GGT7* gene polymorphisms, we attempted to investigate all functionally significant SNPs (eQTL-SNPs) of these genes concerning the association with T2D in a large cohort of the UK Biobank, including two cohorts of patients, such as with non-insulin-dependent diabetes mellitus (19,860 cases and 432,404 controls) and with type 2 diabetes (2889 cases and 449,375 controls). For the association analysis, the GTEx database (date of access: 12 May 2021) was used to select 878 and 1292 significant eQTLs for the *GSS* and *GGT7* genes in skeletal muscle, respectively (no significant eQTLs for the genes were observed in both pancreas and liver). Supplementary Table S3 summarizes the results of SNP–disease associations in the two samples of the UK Biobank. A total of 220 SNPs (almost 20% of the genotyped/imputed SNPs in the cohort) at the *GGT7* gene were found to be associated with the risk of non-insulin-dependent diabetes mellitus at a *p*-value ≤ 0.05 (rs4911408, the top SNP associated at *p* = 9.9 × 10^−5^). Meanwhile, only 54 of 1034 SNPs (about 5% of the genotyped/imputed SNPs) of the *GGT7* gene showed disease association (*p*-value ≤ 0.05) in the type 2 diabetes cohort (rs73099541, the top SNP associated at *p* = 0.01). A relatively fewer number of T2D-associated SNPs were observed for the *GSS* compared to the *GGT7* gene: 53 (about 8% of the genotyped/imputed SNPs) in the NIDDM cohort and only 1 SNP rs78116891 (*p* = 0.02) in the T2D cohort.

### 3.7. Functional Annotation of GSS and GGT7 Gene Polymorphisms

Bioinformatics tools of the eQTLGen Consortium, GTEx portal, and QTLbase were used to assess whether the *GSS* and *GGT7* gene polymorphisms represent significant expression quantitative trait loci (QTL) correlating with a variation of molecular traits such as mRNA expression (eQTL), methylation, histone modification and splicing events. The databases allowed identifying significant eQTLs for each studied polymorphism. The results of tissue-specific eQTL analysis for the *GSS* and *GGT7* gene polymorphisms are summarized in Supplementary Table S4 (full list of statistically significant eQTLs obtained from QTLbase and GTEx portal are shown in Supplementary Tables S5 and S6, respectively). Genetic variation at *GSS* (rs13041792) and *GGT7* (rs11546155 and rs6119534) attracts great interest as the SNPs were found to be associated with the risk of type 2 diabetes in the present study. As can be seen from Supplementary Table S4, the rs13041792-A allele is associated with increased expression of the *GSS* gene only in the liver (*p* = 0.009). Meanwhile, this allele was associated with increased mRNA levels of *MYH7B* (*p* = 4.4 × 10^−8^), *PROCR* (*p* = 2.8 × 10^−10^), and *EDEM2* (*p* = 1.3 × 10^−18^) genes in the pancreas (Supplementary Table S6), *MAP1LC3A* (*p* = 9.9 × 10^−14^), *MYH7B* (*p* = 6.3 × 10^−8^) in adipose tissue, *GGT7* (*p* = 7.5 × 10^−10^), and *NFS1* (*p* = 0.0003) in skeletal muscle, as well as *MYH7B* (*p* = 4.5 × 10^−5^) and *PROCR* (*p* = 1.1 × 10^−6^) in the liver. The pancreatic expression of genes correlated with the T2D-associated SNPs of *GSS* and *GGT7* has attracted the most attention since the organ is primarily involved in the development of type 2 diabetes. Pursing this interest, HaploReg and GTEx databases were used to explore pancreatic eQTLs for SNPs that are in strong linkage disequilibrium (r^2^ > 0.8) with polymorphisms rs11546155 and rs6119534 of *GGT7* and rs13041792 of *GSS* (Supplementary Table S7). Sixteen identified SNPs have been in a strong LD with rs13041792, and all these polymorphisms, especially SNP rs7261969, were associated with the pancreatic expression of *EDEM2*, *PROCR*, and *MYH7B*. Moreover, eight SNPs were also associated with the expression level of *TRPC4AP*, and one SNP (rs6088662) was associated with the expression level of *FER1L4*. As can be seen from Supplementary Table S7, no SNPs with a strong LD were identified for the rs11546155 of the *GGT7* gene. In contrast, fifty-five polymorphisms were strongly linked to SNP rs6119534 of *GGT7*, and they were all associated with the pancreatic expression of *EDEM2*. A majority of these SNPs were associated with the expression level of *PROCR, MYH7B* and also *MAP1LC3A* in the pancreas. Thus, a carriage for the T2D-associated alleles of *GSS* and *GGT7* is related to the expression level of *MYH7B*, *PROCR*, and *EDEM2* in the pancreas as well as with the levels of *MYH7B*, *PROCR*, *EDEM2*, *MAP1LC3A*, *GGT7*, *NFS1*, *PIGU*, *NCOA6*, *TRPC4AP* and *ACSS2* in insulin-sensitive tissues.

The UCSC Genome Browser allowed identifying that all these genes are co-located with *GSS* and *GGT7* at a genomic cluster 20q11.22. Supplementary Table S8 shows that the genomic region spans 50 genes. The enrichment analysis of these genes performed by *Enrichr* has revealed numerous significantly enriched terms overlapping with various pathways, molecular functions, and biological processes that may be linked to the pathogenesis of type 2 diabetes: sulfur amino acid metabolism (*AHCY*, *GSS*, *GGT7*, *NFS1*), regulation of carbohydrate (*EIF6*) and lipid metabolism (*RALY*, *NCOA6*, *ACSS2*, *MMP24*, *EIF6*), glucose transmembrane transport (*MYH7B*), notch signaling pathway (*ITCH*, *MAP1LC3A*), TGF-beta signaling pathway (*ITCH*, *DYNLRB1*), regulation of protein translation (*EIF2S2*, *EIF6*, *UQCC1*), protein ubiquitination and degradation (*ITCH*, *TRPC4AP*, *EDEM2*), endoplasmic reticulum (ER) protein possessing and unfolded protein response (*EDEM2*, *CHMP4B*), quality control of unfolded/misfolded proteins (*PIGU*), regulation of reactive oxygen species (*EIF6*, *ROMO1*), autophagy (*CHMP4B*, *MAP1LC3A*, *TP53INP2*), apoptosis (*E2F1*, *CHMP4B*, *MAP1LC3A*) and host–virus interactions (*ACTL10*, *CHMP4B*, *ITCH*). Notably, some the genes at cluster 20q11.22 overlapped with pathogenetically important pathways for type 2 diabetes: *ASIP* (“Proteins Involved in Diabetes Mellitus Type 2”, “Insulin Regulation of Blood Glucose” and “Insulin-mediated Glucose Transport”), *EDEM2* (“Hyperglycemia and Hyperlipidemia Trigger beta-Cell Apoptosis” and “Nitric Oxide Effects on beta-Cell”) and *EIF6* (“Regulation of Glycolytic Process” and “Response to Insulin”). Thus, a number of genes located at 20q11.22 are involved in the pathways of unfolded protein response and the regulation of proteostasis.

### 3.8. Transcriptome-Wide Association Study for Genes Co-Expressed with GSS and GGT7

**Figure 3:**
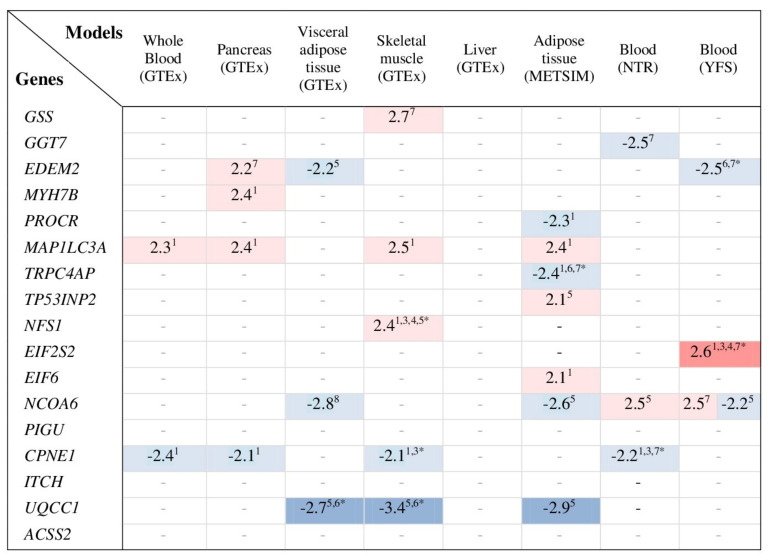
Heatmap of association between gene expression and diabetes traits across different diabetes-related tissues. The heatmap shows Z-score values calculated by bioinformatics tools of the TWAS hub (http://twas-hub.org, date of access 5 July 2021) for an association between expression of genes whose mRNA levels related with the T2D-associated SNPs of *GSS* and *GGT7* genes and diabetes traits in disease-related tissues (* Z-score values averaged from several cited diabetes traits). Color intensity shows the strength of association between gene expression and a trait. The following datasets were utilized to evaluate diabetes-related trait models: (1) Type 2 Diabetes (T2D) (2012), N = 48,761 [59]; (2) Type 2 Diabetes (T2D) (2018) UKBB N = 459324 [60]; (3) Diabetes diagnosed by doctor, UKBB N = 336,473 [www.nealelab.is/uk-biobank, date of access 5 July 2021]; (4) Diabetes (self-reported) N = 337,159 [www.nealelab.is/uk-biobank, date of access 5 July 2021]; (5) Diabetes (father) UKBB N = 293,407 [www.nealelab.is/uk-biobank, date of access 5 July 2021]; (6) Diabetes (mother) UKBB N = 309953 [www.nealelab.is/uk-biobank]; (7) Illnesses of siblings: Diabetes UKBB N = 261075 [www.nealelab.is/uk-biobank, date of access 5 July 2021]; (8) HbA1C N = 46,368 [61]. GTEx (Genotype-Tissue Expression) portal, a comprehensive public resource to study tissue-specific gene expression and regulation [51]; METSIM (METabolic Syndrome In Men), an expression quantitative trait locus (eQTL) study on abdominal subcutaneous adipose tissue [56]; NTR (Netherlands Twin Register), whole blood eQTL data from a large population-based sample including twins, their parents, siblings, spouses, and their adult offspring [57]; YFS (Young Finns Study), a large longitudinal study of cardiovascular risk from childhood to adulthood [58].

A transcriptome-wide association analysis was performed by the TWAS hub to estimate the genetic association between the risk of type 2 diabetes and tissue-specific expression of the genes whose co-expression is related to the disease risk alleles of SNPs of the *GSS* (rs13041792) and *GGT7* (rs6119534 and rs11546155) genes. A heatmap in Figure 3 summarizes statistically significant (Q ≤ 0.05) predictive T2D-association models for genes whose mRNA levels in the pancreas, adipose tissue, skeletal muscle, or whole blood have been related to the T2D-associated SNPs of *GSS* and *GGT7*. In diabetic relatives (parents and/or siblings) from the UK cohort, *EDEM2* expression was increased in the pancreas (Z = 2.2, Q = 0.01) and decreased in visceral adipose tissue (Z = −2.2, Q = 0.01) and blood (Z = −2.5, Q = 0.006). The genetic susceptibility to type 2 diabetes was associated with increased levels of *MYH7B* in pancreatic tissue (Z = 2.4, Q = 0.008). The increased risk of type 2 diabetes was significantly associated with the *MAP1LC3A* expression in various tissues such as the pancreas (Z = 2.4, Q = 0.008), skeletal muscle (Z = 2.5, Q = 0.006), adipose tissue (Z = 2.4, Q = 0.008), and whole blood (Z = 2.3, Q = 0.01). The risk of type 2 diabetes was associated with decreased levels of *CPNE1* in the pancreas (Z = −2.1, Q = 0.02), skeletal muscle (Z = −2.1, Q = 0.02), and blood (Z = −2.3, Q = 0.01). Thus, the TWAS analysis allowed revealing the genetic association between the changes in pancreatic expression of genes such as *EDEM2*, *MYH7B*, *MAP1LC3A*, and *CPNE1* and the risk of type 2 diabetes.

### 3.9. Prediction of Transcription Factors Co-Regulating Expression of T2D Associated Genes and Their Enrichment Analysis

Transcription factors co-regulating the pancreatic expression of *EDEM2*, *MYH7B*, *MAP1LC3A*, and *CPNE1* genes that associated with type 2 diabetes have been predicted by the hTFtarget tool. Supplementary Table S9 shows a list of 107 predicted TFs co-regulating the expression of *MAP1LC3A*, *MYH7B*, *CPNE1*, and *EDEM2* genes. A total of 99 transcription factors were selected by Enrichr (as assessed by the Tissue Expression database and TPM values of the GTEx portal) as being expressed in the pancreas for further functional enrichment analysis to assess whether the selected transcription factors co-regulating expression of *EDEM2*, *MYH7B*, *MAP1LC3A*, and *CPNE1* significantly overlap with annotated gene sets representing known diabetes-related pathways. The enriched terms were manually checked to identify the annotations linked to the pathogenesis of type 2 diabetes such as TGF-beta signaling, notch signaling, Wnt signaling, TP53 signaling, cell cycle, apoptosis, autophagy, SUMOylation, insulin resistance, and energy metabolism (i.e., pathways selected from the KEGG, Reactome, and WikiPathways databases). The functional terms significantly enriched (FDR ≤ 0.05) with the set of TFs are listed in Supplementary Table S10. PPI networks of TFs belonging to the T2D pathways were constructed with the STRING database. For instance, transcription factors such as CREB1, NR1H2, STAT3, NR1H3, FOXO1, and NFKB1 were significantly enriched (Q = 7.6 × 10^−5^) with the term insulin resistance (KEGG), whereas CREB1, HDAC1, EP300, PPARG, NRF1, FOXO3, FOXO1, and GABPA were enriched (Q = 2.1 × 10^−9^) with the term energy metabolism (WikiPathways), pathways involved in the pathogenesis of type 2 diabetes.

**Figure 4:**
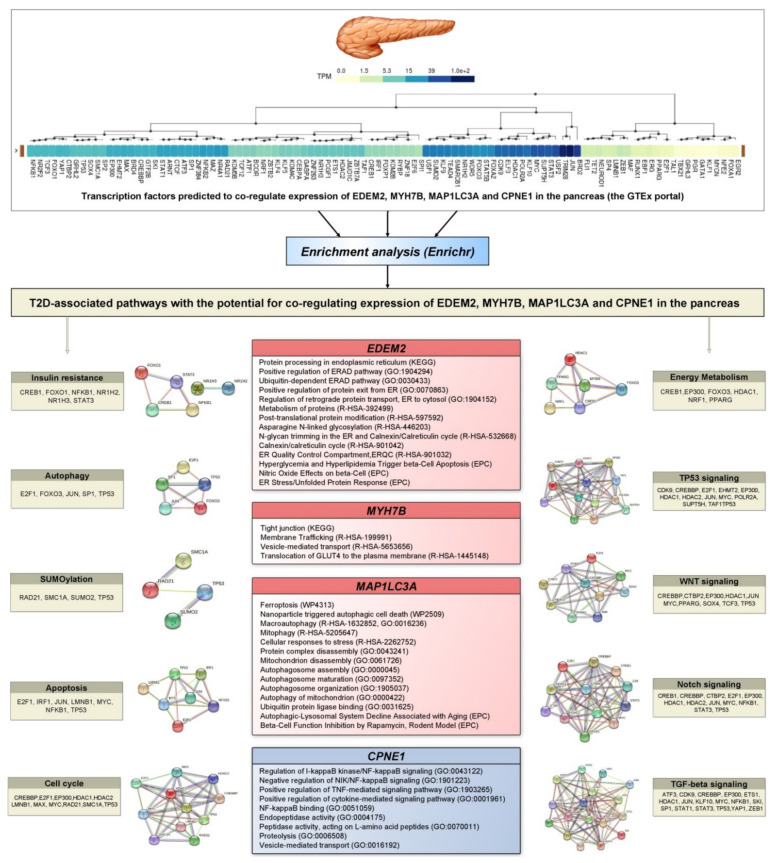
Functional annotation of *EDEM2*, *MYH7B*, *MAP1LC3A* and *CPNE1* genes. The following datasets were utilized to evaluate diabetes-related trait models: (1) type 2 diabetes (T2D) (2012), *n* = 48,761 [59]; (2) type 2 diabetes (T2D) (2018) UKBB *n* = 459,324 [60]; (3) diabetes diagnosed by doctor, UKBB *n* = 336,473; (4) diabetes (self-reported) *n* = 337,159; (5) diabetes (father) UKBB *n* = 293,407; (6) diabetes (mother) UKBB *n* = 309,953; (7) illnesses of siblings: diabetes UKBB *n* = 261,075; (8) HbA1C *n* = 46,368 [61]. The Genotype-Tissue Expression (GTEx) portal, a comprehensive public resource to study tissue-specific gene expression and regulation [51]; Metabolic Syndrome in Men (METSIM), an expression quantitative trait locus (eQTL) study on abdominal subcutaneous adipose tissue [56]; Netherlands Twin Register (NTR), whole-blood eQTL data from a large population-based sample including twins, their parents, siblings, spouses, and their adult offspring [57]; Young Finns Study (YFS), a large longitudinal study of cardiovascular risk from childhood to adulthood [58]. The upper part of the figure depicts clusters of transcription factors (TFs) co-regulating the expression of *EDEM2*, *MYH7B*, *MAP1LC3A*, and *CPNE1* in the pancreas (data obtained from the GTEx portal). The enrichment analysis of the transcription factors is described in Material and Methods. The middle part of the figure includes functional annotations for *EDEM2*, *MYH7B*, *MAP1LC3A*, and *CPNE1* whose expression levels in the pancreas were associated with the risk of type 2 diabetes, as identified by the TWAS analysis (Figure 2). Ten T2D-associated pathways along with their PPI networks are shown around the annotations. The abbreviations used include: KEGG, Kyoto Encyclopedia of Genes and Genomes; GO, Gene Ontology; R-HSA, Reactome Homo Sapiens; WP, WikiPathways; EPC, Elsevier Pathway Collection. Figure was created using the yEd Graph Editor software.

Online resources such as Gene Ontology, KEGG, Reactome, Wikipathways, and Elsevier Pathway Collection (EPC) were used to annotate the biological functions of EDEM2, MYH7B, MAP1LC3A, and CPNE1 (Figure 4). EDEM2 was annotated with multiple terms covering the following biological processes: protein quality control, transport and degradation in the endoplasmic reticulum (R-HSA-392499, R-HSA-901032, R-HSA-901042, GO:1904294, GO:0030433, GO:0070863 and GO:1904152); ER stress/unfolded protein response (R-HSA-901042); and pro-inflammatory (nitric oxide) and pro-apoptotic (hyperglycemia and hyperlipidemia) effects on pancreatic beta-cells. MYH7B was annotated with terms related to the regulation of glucose transport into the cell (KEGG, R-HSA-199991, R-HSA-5653656, and R-HSA-1445148). MAP1LC3A was annotated with terms including autophagy (R-HSA-1632852, GO:0000045, GO:0016236, GO:0097352, GO:1905037, WP2509, R-HSA-5205647), protein/organelle degradation (GO:0031625, GO:0043241, GO:0061726, GO:0000422), apoptosis (WP4313, WP2509), and the age-associated decline of the autophagic–lysosomal system and inhibition of beta-cell function (EPC). CPNE1 was annotated with terms reflecting the repression of NF-kappa-B signaling (GO:0051059, GO:0043122, GO:1901223), the activation of the pro-inflammatory pathway (GO:1903265, GO:0001961), and endopeptidase activity/proteolysis (GO:0004175, GO:0070011, GO:0016192).

## 4. Discussion

### 4.1. Overview of the Main Findings

The present study was designed to test a hypothesis that genetic predisposition to type 2 diabetes is associated with polymorphisms of genes encoding two important enzymes of glutathione metabolism such as glutathione synthetase and gamma-glutamyl transferase 7, and to analyze in silico the potential mechanisms by which these genes contribute to disease pathogenesis. We found that the SNPs rs11546155 and rs6119534 of the *GGT7* are significantly associated with susceptibility to type 2 diabetes regardless of sex, age and body mass index. A polymorphism rs13041792 of the *GSS* gene was also associated with disease risk, but at a borderline significance level. Haplotype analysis revealed that the rs1801310G–rs6088660C–rs13041792A haplotype of *GSS* increased the risk of type 2 diabetes, whereas, on the contrary, two haplotypes such as rs6119534T–rs11546155G and rs6119534C–rs11546155A of *GGT7* decreased disease risk. A sex-specific relationship between the studied gene polymorphisms and biochemical parameters in patients with type 2 diabetics was observed. In particular, SNPs rs13041792 and rs6088660 of *GSS* were associated with increased and decreased levels of fasting blood glucose in diabetic males, respectively. An SNP rs6088660 of *GSS* was associated with decreased levels of hydrogen peroxide in females. An SNP rs1801310 of *GSS* showed association with decreased levels of total glutathione in females. A haplotype rs1801310C–rs6088660T–rs13041792G of *GSS* was associated with increased levels of glutathione in diabetic females. Furthermore, a haplotype rs6119534T–rs11546155G of *GGT7* showed an association with increased levels of reactive oxygen species in both sexes, but more strongly in females than males. In addition, increased levels of total glutathione were associated with haplotypes rs6119534T–rs11546155G and rs6119534C–rs11546155A in females and males, respectively. These correlations were weak and did not survive after adjustment for multiple tests. The MB-MDR method allowed identifying epistatic interactions between the *GSS* and *GGT7* gene polymorphisms contributing to T2D susceptibility, generally between rs13041792, rs1801310, rs6088660 of *GSS*, and rs6119534 of *GGT7*. Eighteen low- and high-risk genotype combinations were found to be significantly associated with type 2 diabetes and two genotype combinations such as *GGT7* rs6119534-C/T × *GSS* rs1801310-A/A *GGT7* and rs6119534-C/T × *GSS* rs13041792-G/G showed the strongest protective effects against disease risk. A replication study in independent populations confirmed an association between SNP rs11546155 of *GGT7* and a decreased risk of type 2 diabetes. In addition, the rest of the SNPs showed associations with T2D-related traits such as body mass index and waist–hip ratio in ethnically distinct populations. A comprehensive bioinformatics analysis was performed to unravel the complex molecular mechanisms by which the glutathione-metabolizing enzymes contribute to the pathogenesis of type 2 diabetes. A tissue-specific eQTL analysis of the GTEx data showed that a carriage for the T2D-associated *GSS* and *GGT7* alleles was correlated with the co-expression of genes encoding *MYH7B*, *PROCR*, *EDEM2*, and some other genes in the pancreas as well as in insulin-sensitive tissues such as skeletal muscle, visceral adipose tissue, and the liver. Transcriptome-wide association study allowed revealing the genetic link between T2D susceptibility and both the increased expression of *EDEM2*, *MYH7B*, *MAP1LC3A* and the decreased expression of *CPNE1* in the pancreas. Importantly, a functional enrichment analysis showed that *EDEM2*, *MYH7B*, *MAP1LC3A*, and *CPNE1* genes are statistically enriched to biological terms including protein quality control, transport, and degradation in the endoplasmic reticulum, endoplasmic reticulum stress/unfolded protein response, autophagy, pro-inflammatory and pro-apoptotic effects on pancreatic beta-cells, the repression of NF-kappa-B signaling, regulation of glucose transport into the cell, protein/organelle degradation, and endopeptidase activity/proteolysis. Numerous transcription factors co-regulating the pancreatic expression of *EDEM2*, *MYH7B*, *MAP1LC3A*, and *CPNE1* genes were predicted in silico. These transcription factors are known to be involved in the pathways playing a crucial role in the pathogenesis of type 2 diabetes such as TGF-beta signaling, notch signaling, Wnt signaling, tp53 signaling, cell cycle, apoptosis, autophagy, SUMOylation, insulin resistance, and energy metabolism.

### 4.2. Genetic and Environmental Factors Responsible for Endogenous Deficiency of Glutathione in Type 2 Diabetes

Several experimental and clinical studies provided a line of evidence for the decreased glutathione levels in various tissues in both animals and patients with type 2 diabetes. In particular, the impaired synthesis and increased loss or degradation of GSH appeared to contribute to β-cell dysfunction and the resulting increase in blood glucose is responsible for further depletion of glutathione and the development of long-term diabetic complications [28,30,65,66,67,68]. In particular, Lutchmansingh with colleagues observed that patients with type 2 diabetes have lower erythrocyte concentrations of the reduced form of glutathione and absolute synthesis rates than healthy controls [30]. It has been observed that the synthesis of glutathione is markedly reduced in uncontrolled type 2 diabetes due to the limited availability of its amino acid precursors, and intracellular GSH levels were found to be restored with cysteine and glycine supplementation [27]. Despite numerous findings of glutathione deficiency in type 2 diabetes, a few studies have been performed to elucidate whether abnormal glutathione metabolism is attributed to decreased activity and/or expression of enzymes catalyzing the synthesis of glutathione. A major portion of biochemical studies [69,70,71,72,73,74] has revealed that patients with type 2 diabetes and prediabetes exhibit the increased blood levels of γ-glutamyltransferase (GGT), and it has been suggested that an increase in GGT concentration represents a sensitive and early biomarker for the development of diabetes. This enzyme cleaves the gamma-glutamyl bond of glutathione and its conjugates, releasing two components for further glutathione synthesis such as glutamate and dipeptide cysteinyl-glycine [75]. However, Mendelian randomization studies did not support a causal relationship between increased blood GGT levels and susceptibility to type 2 diabetes [76,77], suggesting that the relationship could be explained by reverse causality and/or residual confounding. A study of Murakami and coworkers [78] provided evidence that the decreased GSH levels and increased glutathione disulfide (GSSG) levels in the erythrocytes of patients with type 2 diabetics is attributed to the decreased activity of enzymes involved in glutathione metabolism such as glutamate cysteine ligase and glutathione reductase. The authors also observed that diabetics have an approximately 70% decreased rate of outward transport of glutathione disulfide than that of healthy subjects. Similar findings have been obtained by some other studies [79,80,81]. A recent paper by Zhang and colleagues has established that glutathione has the potential to prevent β-cell failure and prediabetes in rats on a long-term high-glucose diet [68]. The depletion of endogenous glutathione was found to be progressively associated with an abnormal glucose tolerance test, which could not be attributed to the impaired secretion of insulin by beta-cells [82]. Our recent study of diabetic patients showed that the plasma levels of GSH are inversely correlated with the content of reactive oxygen species, whereas the GSSG levels directly correlated with fasting blood glucose concentrations, providing additional evidence for the link between glutathione and glucose metabolism [32]. High glucose concentrations were experimentally found to increase levels of intracellular peroxide in human islets and the pancreatic β-cell line and the inhibition of glutamate cysteine ligase augmented the increase in islet peroxide as well as the decrease in insulin mRNA levels, in the secretion in islets and in β-cell line induced by ribose [83]. Thus, many studies stated a decrease in the glutathione levels occurs in type 2 diabetes and its complications, and this condition is linked with disease pathogenesis through increased oxidative stress. However, it is still unclear whether glutathione deficiency is a primary or secondary condition for type 2 diabetes.

The level of glutathione is determined by both the activity and expression of the enzymes involved in the biosynthesis of GSH. As it has been observed by the present study, our previous [31,32,34,35] and some other [36,37,38] studies, the genetic factors contribute to both the levels of glutathione and susceptibility to type 2 diabetes, and single nucleotide polymorphisms at genes encoding enzymes for glutathione metabolism such as glutamate cysteine ligase (catalytic and modifier subunits), gamma-glutamyl cyclotransferase, glutathione S-transferases (mu, pi, and theta classes), gamma-glutamyltransferase 6 and 7 are illustrative examples of that relationship. Although the contribution of each SNP of the *GSS* and *GGT7* genes was low or moderate, the joint effects of the genetic variants on the risk of type 2 diabetes were more pronounced, as has been shown by the analysis of SNP–SNP interactions (Table 4 and Table 5). Further studies will extend the spectrum of functional genetic variants of enzymes and other proteins involved in the regulation of glutathione metabolism that would be attractive objects for investigating the genetic architecture of susceptibility to type 2 diabetes.

Data from the literature indicate that the glutathione synthetic capacity of the pancreas is significantly lower than in other organs and tissues having a high level of metabolic activity such as the liver, skeletal muscles, and kidneys [84,85,86]. According to the GTEx project, the pancreas has a relatively low expression levels of genes encoding enzymes involved in the biosynthesis of glutathione such as *GCLC*, *GCLM*, *GSS*, *GSR*, and *GGCT*, meanwhile the pancreatic expression of genes encoding enzymes catabolizing glutathione such as *GGT1*, *GGT6* and *ANPEP* are substantially higher than those involved in GSH biosynthesis. The liver is the main organ where glutathione is synthesized and transported through the bloodstream to various tissues and organs including the pancreas. This means that the glutathione content in the pancreas depends on the entry of its amino acid precursors into the cells and this process is determined by membrane-associated enzymes such as gamma-glutamyl transferase (GGT1) and aminopeptidase N (ANPEP) which hydrolyze plasma GSH into its constituent amino acids, thereby assisting their transport into the cells [84]. Interestingly, a strong increase in the levels of gamma-glutamyl transferase in plasma [69,76,77] and aminopeptidase mRNA in pancreatic islets [87,88] were found in patients with type 2 diabetes. Working consistently, these membrane enzymes break down extracellular glutathione, making its component amino acids such as cysteine and glycine available to the cells for the de novo synthesis of glutathione [75]. Therefore, we propose that the increased levels of GGT1 and ANPEP may be considered as biomarkers of the intracellular deficiency of glutathione and their adaptive upregulation is aimed to enhance supplementing the cell with GSH precursors.

Although glutathione-metabolizing enzymes have an essential role in determining cellular glutathione content, a very limited number of studies have been undertaken to evaluate the relationship between genetic variation at genes encoding susceptibility to type 2 diabetes [31,32,33,34,35,36,37,38]. No studies have been done to date to investigate the contribution of polymorphisms at the *GSS* and *GGT7* genes to the development of type 2 diabetes. However, at the same time, the DisGeNET database (https://www.disgenet.org, date of access 4 June 2021) comprises research findings for the associations of *GSS* and *GGT7* gene polymorphisms in humans with certain diseases, but not with diabetes. To date, several genetic studies have provided evidence for the association between SNPs of the *GSS* gene and hemolytic anemia [89,90], breast cancer [91], neoplasms [92], bipolar disorder [93], alcohol dependence [94], and plasma levels of protein C [95]. Polymorphisms of the *GGT7* have been associated with coronary artery disease [96], glomerular filtration rate [97], age at menarche [98] and plasma levels of protein C [99].

The present study was the first to assess the contribution of two genes encoding glutathione synthetase and gamma-glutamyl transferase 7 to the development of type 2 diabetes mellitus. Glutathione synthetase is the second enzyme of a two-step pathway, called de novo glutathione biosynthesis, catalyzing the condensation of gamma-glutamylcysteine and glycine, to form reduced glutathione (GSH) [100]. Unlike glutamate cysteine ligase, GSS is not feedback-inhibited by GSH, and its activity is essentially regulated at the level of transcription and substrate availability [101]. Gamma-glutamyl transferase 7 (also known as γ-glutamyltranspeptidase 7) is a membrane-bound extracellular enzyme that cleaves gamma-glutamyl peptide bonds in GSH and transfers the gamma-glutamyl compounds to acceptors [102]. GGT7, similarly to other gamma glutamyl transferases, on the one hand, plays the key role in glutathione homeostasis by providing substrates for glutathione synthesis, especially for those tissues with a low rate of glutathione biosynthesis; on the other hand, the enzyme counteracts oxidative stress by breaking down extracellular glutathione and making its component amino acids available to the cells [75]. This pathway, also called γ-glutamyl cycle, allows for released glutathione to be broken down and its constituent amino acids utilized by cells for de novo synthesis of GSH [103]. Both *GSS* and *GGT7* genes are located at the genomic region 20q11.22. Genes encoding glutathione synthetase and gamma-glutamyl transferase 7 consist of 13 and 17 exons, respectively, which are all differentially expressed in the pancreas (GTEx). It is known that the expression of *GSS* and *GGT7* genes is regulated by transcription factors such as Nrf1 and Nrf2 that are molecular sensors of oxidative stress [104,105].

No functional studies have been conducted to date to analyze the impact of the *GSS* and *GGT7* polymorphisms on the expression or activity of these genes at the transcriptional and/or protein levels, thereby complicating the interpretation of the genotype–phenotype correlations. Therefore, in the present study, we used numerous bioinformatics methods for the functional annotation of SNPs to clarify the phenotypic effects of *GSS* and *GGT7* genes showed significant associations with type 2 diabetes. Surprisingly, none of the studied SNPs were associated with the expression level of *GSS* and *GGT7* genes in the pancreas, as assessed by the public genome–transcriptome databases. Instead, the rs13041792-A allele of *GSS* that increased the T2D risk was found to be associated with an increased expression of *EDEM2*, *MYH7B*, *PROCR* genes in the pancreas as well as *GSS*, *MYH7B*, *PROCR*, and *TRPC4AP* genes in the liver. The TWAS analysis has shown that the pancreatic expression of *EDEM2*, *MYH7B*, *MAP1LC3A*, and *CPNE1* correlated to a carriage of T2D-associated alleles of *GSS* and *GGT7* is associated with the genetic risk of type 2 diabetes. We carried out an in-depth analysis of the literature on the biological functions of genes whose expression is interrelated with polymorphic variants of the *GSS* and *GGT7* genes associated with the development of type 2 diabetes (Supplementary Data S11). An interesting fact is that almost all genes whose expression correlated with the T2D associated variants of *GSS* and *GGT7* represent the pathways involved in the regulation unfolded protein response and proteostasis, as well as in biological processes that play a role in the pathogenesis of type 2 diabetes mellitus.

The absence of significant eQTLs at the *GSS* and *GGT7* genes in the pancreas might be explained by the extremely lower pancreatic expression of these genes (and also other genes encoding glutathione-metabolizing enzymes) in the comparison with other organs and tissues, as assessed by GTEx and Protein Atlas databases. This finding may indicate a limited capacity of the pancreas to produce glutathione at a high level. It is well known that the bulk of plasma glutathione originates from the liver playing a central role in the inter-organ homeostasis of GSH by exporting it into plasma and bile and impacting GSH homeostasis systemically, including the pancreas [103]. Taking into account the importance of these genes for glutathione biosynthesis in the pancreas to ensure the folding of maturating enzymes including insulin, we hypothesize that the absence of genetic variants capable of affecting the pancreatic expression and/or activity of *GSS* and *GGT7* is attributed to the natural selection which recognizes the lower fitness of such variants to intensive protein biosynthesis occurring in this multifunctional organ and, respectively, removes them from the population. Despite the absence of an association of *GSS* and *GGT7* polymorphisms with the level of expression of their own genes, the SNPs were correlated with parameters of redox homeostasis such as glutathione and ROS concentrations, thereby indicating the contribution of these polymorphisms to the regulation of glutathione metabolism. We found that polymorphic variants at *GSS* and *GGT7* genes, alone and in combination, are correlated to the biochemical parameters of redox homeostasis such as ROS and glutathione content as well as to fasting blood glucose and the risk of diabetes. This means that, on the one hand, the studied SNPs are functionally significant variants at *GSS* and *GGT7* genes and may impact on glutathione metabolism; on the other hand, the functional effect of a particular SNP could be explained by linkage disequilibrium with a nearby locus such as *EDEM2*, *MYH7B*, *PROCR* and *MAP1LC3A*. For instance, a polymorphism rs13041792 is located at 1.4kb 5′ of the *GSS* gene and is in a strong linkage disequilibrium (D’ ≥ 0.91) to SNPs situated near or in neighboring genes such as *MYH7B* (rs6120772, rs6120775, rs7261969, rs6088667, rs3746446, rs7268266, rs6120788, rs3746436 and rs3746435), *MIR499A* (rs3746444) and *TRPC4AP* (rs752075), and certain of these SNPs tightly linked to a polymorphism rs13041792 of the *GSS* gene are associated with the risk of several common diseases (Supplementary Data S12). The observed association between T2D and haplotype rs1801310G–rs6088660C–rs13041792A of *GSS* might be interpreted by the presence of an allele rs6088660-C which is associated with the decreased pancreatic expression of *GSS* or by the linkage disequilibrium of rs13041792 to some other SNP with a causative effect on the disease. The pathogenetic relationship between the *GSS* gene polymorphisms and T2D susceptibility was also supported by the findings that the rs6088660-C and rs13041792-A alleles are associated with increased levels of fasting blood glucose. The *GGT7* haplotypes were associated with increased levels of ROS (rs6119534-T–rs11546155-G) and total glutathione (rs6119534-T–rs11546155-G and rs6119534-C–rs11546155-A). These associations may reflect that the increased activity or expression of membrane-associated gamma-glutamyl transferase 7 has a known adaptive role against oxidative stress through providing the cell with amino acid precursors derived from a cleavage of extracellular GSH [75]. Therefore, the protective effects of the *GGT7* haplotypes against the risk of type 2 diabetes may be attributed to the *GGT7*-mediated increase in the de novo biosynthesis of glutathione. Thus, the associations of *GGT7* and *GSS* gene polymorphisms with the levels of glutathione and ROS show that the genes have the potential to impact the glutathione homeostasis and loss-of-function effects of their alleles, may contribute to the reduced synthesis of glutathione in the cell, and may play a role in the pathogenesis of type 2 diabetes. Furthermore, we observed sex-specific effects of the *GSS* alleles on the redox parameters: females with the rs1801310A allele, associated with a decreased *GSS* expression, had a lower plasma glutathione concentration than female carriers for a wild-type allele. Females with the rs6088660T allele (associated with increased levels of *GSS*) had a decreased level of ROS and fasting blood glucose. Meantime, SNP rs13041792 influenced the risk of T2D, and was also associated with increased concentration of fasting blood glucose—but in males. The above findings demonstrate sex dimorphism in the impact of genetic variants of glutathione-metabolizing enzyme both on redox and glucose homeostasis. Notably, human and animal studies have shown that the activity of glutathione-metabolizing enzymes such as glutamate cysteine ligase [106], glutathione reductase [107], glutathione peroxidase [108], glutathione S-transferase [109] is higher in females than males. Thus, these findings were expected since sex-dependent differences were documented in both glutathione metabolism and glutathione-dependent responses, and the sex-specific genotype–phenotype correlations may be attributed to the complex interactions between genetic factors, levels of oxidative stress, and sex hormones [110]. Finally, multiple environmental factors such as poor diet, physical inactivity, life stress, smoking, and chemical agents have been found to deplete the endogenous glutathione pool, and also contribute to the development of type 2 diabetes (Supplementary Data S13).

### 4.3. Glutathione Deficiency as a Cause of Impaired Folding of Proinsulin in Type 2 Diabetes

Glutathione is a tripeptide consisting of three amino acids such as cysteine, glycine, and glutamate. GSH represents the most abundant non-protein thiol in cells, serving as a major reducing agent as well as an antioxidant defense against oxidative damage of cells [111]. Intracellular concentrations of reduced glutathione are maintained at the highest (millimolar) levels suggesting its vital and multifaceted roles, not solely limited by antioxidant defense [20]. Glutathione possesses a plethora of functions essential for whole-body homeostasis including the detoxification of both xenobiotic and endogenous compounds as well as the maintenance of the mitochondrial redox environment, antiviral defense by fine-tuning the innate immune response to antiviral pathways, the regeneration of vitamins C and E and the regulation of cellular proliferation, apoptosis and protein folding [20,111,112,113,114,115,116,117]. Glutathione deficiency is well known for increasing oxidative stress, a pathological condition capable of disturbing the redox state in the endoplasmic reticulum and disrupt disulfide bond formation, adaptively activating ER stress and unfolded protein response [118,119]. In parallel, a loss of glutathione was found to be associated with the impairment of the electron transport chain function in mitochondria and leading to mitochondrial dysfunction [119,120]. Notably, oxidative stress and mitochondrial dysfunction are both involved in T2D pathogenesis.

**Figure 5:**
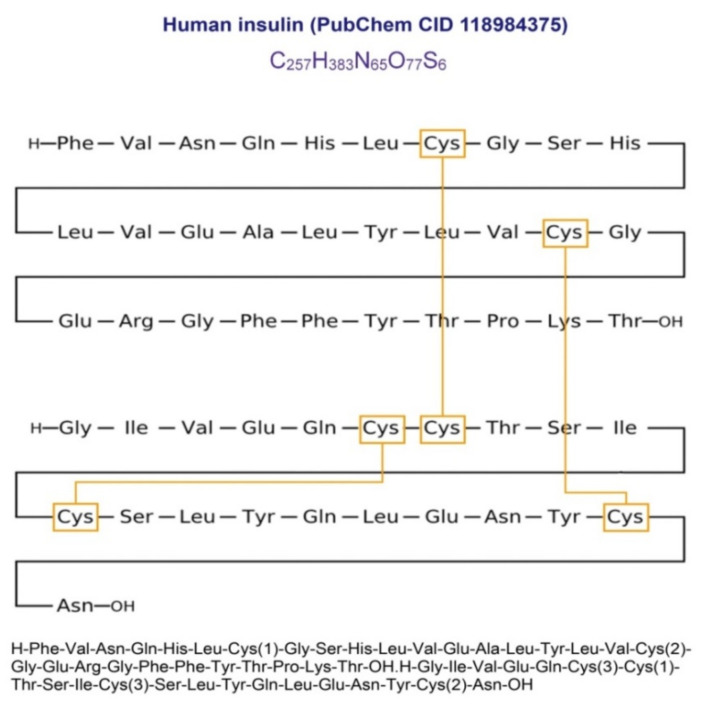
The chemical structure of human insulin. Three disulfide bonds in a molecule of human insulin are indicated by orange lines. The figure was adapted from the Biologic Description of the PubChem database (https://pubchem.ncbi.nlm.nih.gov, PubChem CID 118984375, date of access 14 June 2020).

The formation of native disulfide bonds is an important process underlying the maturation of proteins that enter the secretory pathway [117]. Newly synthesized proteins enter the endoplasmic reticulum where they undergo a series of modifications, among which folding is a critical process required for ultimate protein maturation and subsequent release from the ER [121]. It is important to note that protein folding and disulfide bond formation in maturated proteins occur simultaneously. It is known that protein folding in the ER requires, on the one hand, effective protein thiol oxidation, and on the other hand, reductive processes for editing disulfides when maturated proteins secrete or degrade [122,123]. Notably, both processes are under the control of intracellular glutathione. Glutathione has a strong influence on disulfide pairing in a target protein providing the formation of its native tertiary structure [122,123,124,125,126]. Interestingly, oxidized (GSSG) and reduced (GSH) forms of glutathione play distinct functions in the process of protein folding: GSSG acting as an oxidant provides disulfide bond formation in protein, whereas GSH functions as a reducing agent that cleavages mis-bridged disulfide bonds [127,128]. These properties of glutathione are widely used by in vitro studies investigating the folding reactions of disulfide-containing proteins [129,130]. Proteins containing cysteine residues including insulin (Figure 5) are usually folded into the native conformation via the formation of complex disulfide intermediates requiring a sufficient amount of glutathione molecules [126]. Thus, taking into account the critical role of glutathione in ensuring protein folding, there is a reason to believe that a deficiency of glutathione in the cell, especially in the endoplasmic reticulum, maybe the key factor responsible for ineffective protein folding, ultimately leading to the accumulation of unfolded or misfolded proteins in the ER, protein overload and the subsequent activation of unfolded protein response. It is important to note that proinsulin folding is closely tuned to ER stress and unfolded protein response pathways that may play a role in the damage of pancreatic beta-cells in type 2 diabetes [131,132,133].

Unfolded protein response is an evolutionary conserved cellular stress response that is initiated as a result of the endoplasmic reticulum stress that is activated due to an accumulation of unfolded or misfolded proteins in the ER lumen, aiming to maintain cell viability and function [134]. UPR is activated to adjust and match the endoplasmic reticulum’s protein-folding capacity by inhibiting protein translation that prevents protein overload in the ER, degrading misfolded or unfolded proteins, and activating pathways that increase the production of molecular chaperones aiding protein folding [134,135]. In the beta-cells, the UPR may have a crucial role as a quality control system for proper proinsulin folding by selecting correctly folded insulin molecules, their transport across the cell membrane, and secretion into the bloodstream. Prolonged and/or severe ER stress, as well as the inability of the UPR to restore protein-folding homeostasis, results in the activation of numerous signaling pathways that induce cell apoptosis.

**Figure 6:**
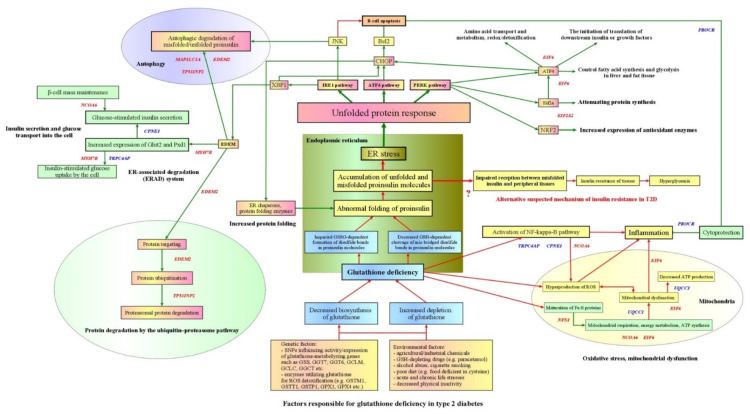
Proposed mechanisms by which glutathione deficiency contributes to the pathogenesis of type 2 diabetes through the activation of UPR. Genes whose expressions change showing an association with type 2 diabetes as a result of TWAS analysis are tied to specific biological and pathological processes that are important for disease development. Blue and red colors depict genes whose tissue expression in diabetics is decreased and increased, respectively. The detailed information on genes present in the figure is summarized in Supplementary Data S11. Figure was created using the yEd Graph Editor software.

The important finding of our study was the relationship between T2D-associated SNPs of *GSS* and *GGT7* genes and multi-tissue expression levels of various genes located at the same chromosome segment 20q11.22. In addition, further TWAS analysis has revealed that the increased pancreatic expression of *EDEM2*, *MYH7B*, *MAP1LC3A* and decreased levels of *CPNE1* are associated with the genetic risk of type 2 diabetes. These genes as well as other genes whose expression level in insulin-sensitive tissues correlated with the T2D-associated SNPs of *GSS* and *GGT7* genes are both linked to the pathogenesis of diabetes and represent the arms of a common metabolic pathway-unfolded protein response. Based on extensive literature data and our findings, we hypothesize that unfolded protein response in type 2 diabetes represents a cellular response to glutathione deficiency which is determined by the joint effects of genes encoding glutathione-metabolizing enzymes and environmental factors that deplete the endogenous GSH pool (Figure 6). This suggestion could be supported by enrichment analysis showing that *EDEM2*, *MYH7B*, *MAP1LC3A*, and *CPNE1* genes are overrepresented with biological terms such as protein quality control, transport, and degradation in the endoplasmic reticulum, endoplasmic reticulum stress/unfolded protein response, autophagy, pro-inflammatory and pro-apoptotic effects on pancreatic beta-cells, repression of NF-kappa-B signaling, regulation of glucose transport into the cell, protein/organelle degradation, and endopeptidase activity/proteolysis. All these pathways are the well-recognized arms of UPR. As can be seen from Figure 6, genes whose expression in the pancreas and insulin-sensitive tissues correlated to T2D-associated SNPs of *GSS* and *GGT7* genes are categorized into several groups concerning their enriched biological functions and possible roles in disease pathogenesis: autophagic degradation of misfolded/unfolded proinsulin (TP53INP2, MAP1LC3A and EDEM2), protein degradation by the ubiquitin–proteasome pathway (EDEM2 and TP53INP2), oxidative stress, inflammation and mitochondrial dysfunction (TRPC4AP, CPNE1, NCOA6, NFS1, UQCC1, PROCR and EIF6), insulin secretion and glucose transport into the cell (NCOA6, MYH7B, TRPC4AP and CPNE1), regulation of translation attenuating protein synthesis, amino acid transport and metabolism as well as redox/detoxification (EIF6 and EIF2S2). In addition, the in silico analysis allowed predicting a variety of transcription factors that have the potential to co-regulate the pancreatic expression of *EDEM2*, *MYH7B*, *MAP1LC3A*, and *CPNE1* genes, and these TFs represent the pathways playing a key role in the development of type 2 diabetes such as TGF-beta signaling, notch signaling, Wnt Signaling, TP53 signaling, cell cycle, apoptosis, autophagy, SUMOylation, insulin resistance and energy metabolism (Figure 4). Genomic region 20q11.22 is a subject of great interest because it comprises, in addition to *GSS* and *GGT7*, genes that are related to unfolded protein response. The UPR pathway integrates many biological processes known to play a role in the pathogenesis of type 2 diabetes such as the transport of glucose across the cell membrane, insulin-stimulated glucose uptake by the cell, glucose and energy metabolism, inflammation, autophagy, apoptosis, ER-associated ubiquitin- and proteasome protein degradation, glutathione and sulfur amino acid metabolism. Interestingly, genes such as *GDF5*, *CEP250*, *ERGIC3*, *FER1L4*, *SPAG4*, *CPNE1*, and *RBM12* at 20q11.22 were enriched with a term BMI-adjusted waist-to-hip ratio (GWAS catalog), a trait which is known to be associated with diabetes risk [136] as well as polymorphisms rs143384 and rs224333 of the *GDF5* gene, as established by genome-wide association studies performed on type 2 diabetes [137,138]. Three genes also located in the genomic region 20q11–22at such as *PROCR*, *PIGU*, *ITCH*, and *ACSS2* were also correlated with the disease-associated alleles of *GSS* and *GGT7* genes. PROCR (previously denoted by the symbol EPCR), endothelial protein C receptor, represents a type 1 transmembrane glycoprotein receptor required for the activation of protein C, known as an anti-coagulant serine protease which is involved in the blood coagulation pathway [139]. Moreover, activated protein C (APC) possesses a variety of endothelium-protective functions including cytoprotective and anti-inflammatory effects, maintaining the integrity of the endothelial barrier, and preventing endothelial cell apoptosis [140]. Some studies provided evidence for the protective effect of APC in hyperglycemic conditions. In particular, an experimental study in mice showed that PROCR and protein C were found to ameliorate diabetic nephropathy by inhibiting hyperglycemia-induced endothelial and glomerular apoptosis [141]. The cytoprotective and anti-inflammatory functions of APC have been demonstrated by Contreras and co-workers who reported that the administration of APC significantly reduced a loss of functional islet mass after intraportal transplantation in diabetic mice [142]. In addition, APC-administrated animals exhibited better glucose control, higher glucose disposal rates, and higher acute insulin release. Therefore, it can be assumed that the deficiency of PROCR may contribute to apoptosis-mediated beta-cell loss. Phosphatidylinositol glycan anchor biosynthesis class U (PIGU) is a component of the glycosylphosphatidylinositol (GPI) transamidase complex that attaches certain membrane proteins to the lipid bilayer of the cell membrane [143]. Although the exact functions of PIGU in the GPI transamidase complex need to be elucidated, nevertheless, several studies discussed above showed that PIGU may influence the activity of the complex towards the quality control of unfolded or misfolded proteins designed for ERAD degradation. ITCH is an itchy E3 ubiquitin-protein ligase, accepting ubiquitin from an E3 ubiquitin-conjugating enzyme to transfer it to targeted substrates thereby playing a role in ubiquitination and proteasomal degradation of proteins [144]. ITCH is involved in the control of a wide range of inflammatory pathways including NF-kappaB signaling [145]. ITCH is capable of regulating apoptosis and the levels of reactive oxygen species through the ubiquitination and proteasomal degradation of the thioredoxin interacting protein (TXNIP), which is known to inhibit the antioxidative function of thioredoxin resulting in ROS accumulation and oxidative stress [146]. Acyl-CoA synthetase short chain family member 2 (ACSS2) constitutes a cytosolic enzyme catalyzing the ATP-dependent synthesis of acetyl coenzyme A (acetyl-CoA), a molecule that participates in the metabolism of proteins, lipid and carbohydrates. The main function of acetyl-CoA is supplying the Krebs cycle with the acetyl group to be oxidized for energy production and the abundance of acetyl-CoA in distinct subcellular compartments is considered as a marker for the general energetic state of the cell [147]. Martínez-Micaelo with co-workers [148] has identified that ACSS2 represents a part of the nutrient-sensing system that influences inflammatory responses. In addition, ACSS2 has been proven to affect autophagy through a translocation into the nucleus and the upregulation of the promoter regions of lysosomal and autophagy genes [149]. The depletion of acetyl-CoA was found to induce autophagy whereas an excess of acetyl-CoA inhibits autophagy [150]. Thus, altogether, the study findings clearly show that the gene cluster spanning 20q11.22 is closely related to the development of type 2 diabetes and represents a tandem of genes whose coordinated expression is most likely necessary for the control and regulation of the adaptive cellular response to endoplasmic reticulum stress caused by the accumulation of unfolded and misfolded proteins in the ER lumen. The coordinated expression of these genes is very important for the pancreas, an organ with exocrine and endocrine functions, and with the highest rate of protein synthesis (enzymes and hormones) that requires the organization of a reliable and evolutionally conserved system that provides post-translational protein modifications including disulfide bridge formation, the isomerization of peptide bonds, the hydrolysis of polypeptides, and the phosphorylation, ubiquitination, sumoylation and other biological processes aiming to acquire protein’s proper structure and function [151,152]. The low pancreatic expression of *GSS*, *GGT7*, and other genes encoding glutathione-metabolizing enzymes (GTEx portal), the absence of evolutionarily conserved alleles affecting the expression and/or activity of these genes in the population, as well as the correlation of the *GSS* and *GGT7* alleles with the expression of genes that are targeted by unfolded protein response all clearly indicate that pancreatic cells are very sensitive to changes in GSH content and appear to have a limited capacity to restore native S–S bonds in proteins under the condition of a deficiency of glutathione, which is an essential substrate for the formation of disulfide bridges and their correction in the case of their mis-bridged state [117,127,128]. In this context, it can be assumed that the adaptive response of pancreatic cells to impaired protein folding, the accumulation of unfolded and misfolded proteins, and the resulting ER stress is mediated through the activation of UPR aimed at restoring protein folding by the mechanisms including chaperone-assisted protein folding, a reduction in protein synthesis, and enhancing ER-associated protein degradation. If intracellular glutathione content is not restored to the levels necessary for the effective formation of disulfide bonds in proteins and unfolded or misfolded proteins continue to be accumulated in the ER, the UPR pathway may induce ER stress-associated programmed cell death.

### 4.4. Study Limitations

Our study has some limitations. First, the parameters of redox homeostasis including ROS (hydrogen peroxide) and total glutathione levels were measured in plasma and only in a subgroup of patients and controls (in total, 578 subjects), and this was the reason why we did not obtain more reliable correlations between the genotypes and biochemical characteristics in diabetics. Second, the eQTL and TWAS analyses were performed using the transcriptomic data obtained from the whole pancreas, not from beta-cells or even pancreatic islets. Therefore, the observed genotype–phenotype correlations associated with T2D susceptibility should be interpreted with caution and warrant independent confirmation by transcriptomic studies on pancreatic islets or beta-cells obtained from diabetics. Finally, we did not investigate SNP–environment interactions to evaluate what kind of known environmental risk factors for T2D (for instance, physical inactivity and poor nutrition) have synergic effects on disease development in individuals with disease-related *GSS* and *GGT7* genotypes. Undoubtedly, our hypothesis that glutathione deficiency is a trigger for type 2 diabetes needs experimental validation. It is unknown whether three evolutionarily conserved disulfide bridges in a molecule of human insulin are critical for its proper folding and if so, glutathione deficiency in the ER might be an essential factor in disrupting the formation of disulfide bonds in proinsulin that enters intermolecular disulfide-linked complexes The question remains of whether unfolded or misfolded insulin can pass the quality control system of protein folding and be secreted into the bloodstream—and if so, whether the insulin resistance of tissues in type 2 diabetes is attributed to the inability of unfolded/misfolded insulin to interact with insulin receptors in peripheral tissues? We believe that many of these important questions will be answered in further experimental and clinical studies.

## 5. Conclusions and Perspectives

The present study demonstrated for the first time that genetic variants in glutathione synthetase and gamma-glutamyl transferase 7 genes represent novel susceptibility markers for type 2 diabetes with the potential to influence disease risk through an impairment of glutathione metabolism. A comprehensive bioinformatics analysis allowed suggesting that *GSS* and *GGT7* genes contribute to disease pathogenesis through intracellular glutathione deficiency, a condition that we believe is a primary factor responsible for the activation of unfolding protein response and subsequent cell death pathways as a result of the accumulation of unfolded and/or misfolded proinsulin molecules in the endoplasmic reticulum of pancreatic beta-cells. The results of the present study and our previous studies suggest that the endogenous deficiency of glutathione could be the key etiological factor underlying type 2 diabetes and polymorphisms in genes encoding enzymes involved in the metabolism of glutathione, such as glutathione synthetase, gamma-glutamyltransferase-7 and other glutathione-metabolizing enzymes may be considered as important modifiers of the genetic susceptibility to this disease. Glutathione deficiency in diabetics seems to occur in many tissues, however, the degree of glutathione deficiency and cellular response to this condition can vary from tissue to tissue depending on the tissue-specific levels of expression of genes encoding glutathione-metabolizing enzymes. Importantly, the risk alleles at *GSS* and *GGT7* are correlated with the tissue-specific expression of genes involved in the unfolded protein response pathway and regulation of proteostasis, suggesting that glutathione deficiency in pancreatic beta-cells might be an essential triggering factor responsible for an impaired folding of proinsulin, a condition that has been documented in type 2 diabetes by several studies [153,154,155,156,157]. This assumption is supported by the accumulation of unfolded protein masses and amyloids that are known to be diabetogenic factors responsible for both the induction of apoptosis and the progressive functional incompetence of beta-cells [158]. In addition, in beta-cells undergoing ER stress and accumulating toxic oligomers of the unfolded protein, glucose metabolism is remodeled under conditions of hyperglycemia [159]. It is noteworthy that glutathione has also been shown to prevent beta-cell dedifferentiation and failure caused by chronic oscillating glucose intake [68].

Certain clinical and experimental studies have already established that replenishing the endogenous deficiency of glutathione is a promising strategy in the treatment and prevention of type 2 diabetes and its complications [66,68,160,161]. We believe that polymorphisms of genes encoding glutathione-metabolizing enzymes represent attractive biomarkers for the predictive genetic testing of impaired glutathione metabolism and could be used for assessing the susceptibility of an individual to numerous pathological conditions associated with glutathione deficiency such as diabetes [27,30], cardiovascular diseases [162,163], cancer [164], liver diseases [165], neurodegenerative disorders [166] as well as COVID-19 [167,168]. Genetic polymorphisms of *GSS* and *GGT7* and other glutathione-metabolizing enzymes may become attractive markers for pharmacogenetic studies of type 2 diabetes and its complications [169,170] and further studies are required to focus on a comprehensive evaluation of genes affecting glutathione metabolism for their joint effects on glutathione metabolism and their contribution to the development and progression of type 2 diabetes.

## Figures and Tables

**Table 1:** Demographic and clinical characteristics of the study participants.

Baseline Characteristics		Healthy Controls, *n* = 1626	T2D Patients, *n* = 1572	*p*-Value
Age, mean ± standard deviation		60.8 ± 6.4	61.3 ± 10.4	0.34
Sex	Males, *n* (%)	601 (37.0)	584 (37.2)	0.89
	Females, *n* (%)	1024 (63.0)	988 (62.8)	
Body mass index (kg/m^2^), mean ± standard deviation		27.21 ± 3.55	31.92 ± 6.65	**0.001**
Duration of diabetes, median (Q1; Q3)		-	9.0 (3.0; 15.0)	-
Hypertension, *n* (%)		24 (1.5)	1340 (85.2)	**<0.0001**
CAD, *n* (%)		-	511 (32.5)	**<0.0001**
Smokers (ever/never), *n* (%)		501 (31.0%)	412 (26.1%)	**0.004**
Positive family history of diabetes, *n* (%)		33 (2.1%)	602 (38.1%)	**<0.0001**
HbA_1C_ (%), Me (Q1; Q3)		4.58 (4.11; 4.87)	9.02 (7.70; 10.80)	**<0.0001**
FBG (mmol/L), Me (Q1; Q3)		4.71 (4.39; 4.84)	12.20 (9.70; 15.20)	**<0.0001**
Total cholesterol (mmol/L), Me (Q1; Q3)		3.06 (2.86; 3.12)	5.10 (4.27; 6.09)	**<0.0001**
LDL (mmol/L), Me (Q1; Q3)		1.74 (1.60; 1.79)	3.03 (2.40; 4.05)	**<0.0001**
HDL (mmol/L), Me (Q1; Q3)		1.47 (1.36; 1.62)	0.85 (0.74; 1.07)	**<0.0001**
Triacylglycerides (mmol/L), Me (Q1; Q3)		1.15 (0.98; 1.23)	2.20 (1.55; 3.00)	**<0.0001**
H_2_O_2_ (mmol/L), Me(Q1;Q3)		2.81 (2.18; 3.63)	3.70 (2.65; 4.97)	**<0.0001**
GSSG/GSH (mmol/L), Me(Q1;Q3)		1.91 (0.83; 5.41)	1.63 (0.56; 3.79)	**0.037**

CAD, coronary artery disease; HbA_1C_, glycated hemoglobin; FBP, fasting blood glucose; LDL, low-density lipoproteins; HDL, high-density lipoproteins; H_2_O_2_, hydrogen peroxide; GSSG/GSH, total glutathione. Bold indicates statistically significant *p*-values.

**Table 2:** Genotype and allele frequencies for the studied SNPs among T2D patients and healthy controls.

Gene, SNP	Genotype, Allele	Healthy Controls, *n* = 1626 *n* (%) ^1^	T2D Patients, *n* = 1572 *n* (%) ^1^	OR (95% CI) ^2^	*p*-Value ^2^	_adj_OR (95% CI) ^3^	*p*-Value ^3^
*GSS* rs13041792 G > A	G/G	1022 (66)	957 (61.9)	1.00	**0.046**	1.00	**0.027**
	G/A	460 (29.7)	523 (33.8)	1.21 (1.04–1.42)		1.24 (1.06–1.44)	
	A/A	66 (4.3)	66 (4.3)	1.07 (0.75–1.52)		1.07 (0.75–1.53)	
	A	0.191	0.212	1.14 (1.01–1.29)	**0.043**	-	**0.05**
*GSS* rs1801310 G > A	G/G	590 (36.4)	581 (36.8)	1.00	0.16	1.00	0.18
	G/A	761 (47)	773 (49)	1.03 (0.89–1.20)		1.04 (0.89–1.21)	
	A/A	270 (16.7)	225 (14.2)	0.85 (0.69–1.04)		0.85 (0.69–1.06)	
	A	0.401	0.387	0.94 (0.85–1.04)	0.25	-	0.38
*GSS* rs6088660 C > T	C/C	792 (50.5)	815 (52.4)	1.00	0.44	1.00	0.43
	C/T	647 (41.3)	628 (40.4)	0.94 (0.81–1.09)		0.94 (0.81–1.09)	
	T/T	128 (8.2)	112 (7.2)	0.85 (0.65–1.12)		0.85 (0.64–1.12)	
	T	0.288	0.274	0.93 (0.83–1.04)	0.21	-	0.26
*GGT7* rs11546155 G > A	G/G	1297 (80.5)	1283 (81.3)	1.00	**0.013**	1.00	**0.022**
	G/A	282 (17.5)	282 (17.9)	1.01 (0.84–1.21)		1.02 (0.85–1.22)	
	A/A	33 (2)	13 (0.8)	0.40 (0.21–0.76)		0.42 (0.22–0.80)	
	A	0.108	0.098	0.89 (0.76–1.05)	0.17		0.21
*GGT7* rs6119534 C > T	C/C	241 (19.8)	352 (27.1)	1.00	**0.0001**	1.00	**0.0003**
	C/T	864 (71)	832 (64.2)	0.63 (0.51–0.79)		0.67 (0.56–0.82)	
	T/T	111 (9.1)	113 (8.7)	0.62 (0.43–0.88)		0.73 (0.53–1.00)	
	T	0.447	0.408	0.85 (0.76–0.95)	**0.006**	-	**0.012**

^1^ Absolute number and percentage of individuals/chromosomes with a particular genotype/allele. ^2^ Odds ratio with 95% confidence intervals (crude analysis) with one degree of freedom. ^3^ Odds ratio with 95% confidence intervals adjusted for age, sex and BMI with one degree of freedom. Bold indicates statistically significant *p*-values/ORs (95%CI).

**Table 3:** Estimated common haplotype frequencies of *GSS* and *GGT7* genes in T2D patients and controls.

**Haplotypes of the *GSS* Gene (Global Haplotype Association *p*-Value 0.039)**							
**rs1801310**	**rs6088660**		**rs13041792**	**Healthy Controls** **(*n* = 1626)**	**T2D Patients** **(*n* = 1572)**	**OR** (95% CI) *	***p*-Value** **(Q-Value)**
A	C		G	0.3915	0.3782	1.00	-
G	T		G	0.2823	0.2713	1.02 (0.89–1.17)	0.78 (0.91)
G	C		A	0.1821	0.203	**1.26 (1.08–1.47)**	**0.003 (0.011)**
G	C		G	0.1334	0.1373	0.99 (0.83–1.18)	0.92 (0.92)
Haplotypes of the *GGT7* Gene (Global Haplotype Association *p*-Value 0.0018)							
rs6119534		rs11546155		Healthy Controls (*n* = 1626)	T2D patients (*n* = 1572)	OR (95% CI) *	*p*-value (Q-value)
C		G		0.4570	0.4992	1.00	-
T		G		0.4350	0.4032	**0.80 (0.68–0.95)**	**0.009 (0.026)**
C		A		0.0971	0.0931	**0.76 (0.61–0.94)**	**0.011 (0.026)**

* Odds ratio with 95% confidence intervals adjusted for age, sex and BMI. Rare haplotypes (frequency < 1%) are not shown. T2D, type 2 diabetes; OR, odds ratio; CI, confidence interval. Bold is statistically significant *p*- and Q-values.

**Table 4:** Two-order SNP–SNP interaction mbmdr-models associated with the risk of type 2 diabetes.

SNP–SNP Interaction *mbmdr*-Models		NH	*β* H	WH	NL	*β* L	WL	*p*_perm_
1	*GGT7* rs6119534 × *GSS* rs13041792	2	0.111	30.53	1	−0.118	32.37	**<0.001**
2	*GGT7* rs6119534 × *GSS* rs1801310	2	0.104	17.66	1	−0.256	22.74	**<0.001**
3	*GGT7* rs11546155 × *GGT7* rs6119534	2	0.109	21.07	3	−0.079	13.93	**<0.001**
4	*GGT7* rs6119534 × *GSS* rs6088660	2	0.107	20.08	2	−0.060	8.36	**<0.001**
5	*GGT7* rs11546155 × *GSS* rs6088660	0	NA	NA	1	−0.319	8.93	**0.034**
6	*GGT7* rs11546155 × *GSS* rs1801310	0	NA	NA	1	−0.223	7.89	**0.046**
7	*GGT7* rs11546155 × *GSS* rs13041792	1	0.040	4.05	1	−0.208	7.52	**0.046**

Models are obtained using the model-based multifactor dimensionality reduction method (MB-MDR package for R). *β* H, regression coefficient for high-risk exposition in the step 2 analysis; *β* L, regression coefficient for low-risk exposition in the step 2 analysis; NH, number of significant high-risk genotypes in the interaction; NL, number of significant low-risk genotypes in the interaction; *p*_perm_, permutation *p*-value for the interaction model. The models were adjusted for age, sex and BMI; WH, Wald statistic for the high-risk category; WL, Wald statistic for the low-risk category.

**Table 5:** Association analysis of genotype combinations with the risk of type 2 diabetes.

No.	Genotype Combination	T2D Patients		Healthy Controls		OR (95% CI) ^1^	*p* ^2^	*Q* ^3^
		N	%	N	%			
G1	*GGT7* rs6119534-C/C × *GSS* rs13041792-G/G	333	26.1	223	18.9	1.52 (1.25–1.84)	**0.00002**	**0.0001**
G2	*GGT7* rs6119534-C/T × *GSS* rs13041792-G/G	409	32.1	509	43.1	0.62 (0.53–0.73)	**1.6 × 10^−8^**	**2.9 × 10^−7^**
G3	*GGT7* rs6119534-C/T × *GSS* rs13041792-G/A	379	29.7	305	25.8	1.21 (1.02–1.45)	**0.03**	**0.04**
G4	*GGT7* rs6119534-C/C × *GSS* rs1801310-G/A	168	13.0	109	9.0	1.51 (1.17–1.94)	**0.002**	**0.01**
G5	*GGT7* rs6119534-C/C × *GSS* rs1801310-A/A	135	10.4	93	7.7	1.40 (1.06–1.84)	**0.017**	**0.03**
G6	*GGT7* rs6119534-C/T × *GSS* rs1801310-A/A	24	1.9	65	5.4	0.33 (0.21–0.54)	**2.0 × 10^−6^**	**1.8 × 10^−5^**
G7	*GGT7* rs6119534-C/C × *GGT7* rs11546155-G/G	247	19.0	163	13.4	1.52 (1.22–1.88)	**0.0001**	**0.0005**
G8	*GGT7* rs6119534-C/C × *GGT7* rs11546155-G/A	98	7.6	67	5.5	1.40 (1.02–1.93)	**0.04**	**0.042**
G9	*GGT7* rs6119534-C/T × *GGT7* rs11546155-G/G	696	53.7	703	57.9	0.84 (0.72–0.99)	**0.03**	**0.04**
G10	*GGT7* rs6119534-C/T × *GGT7* rs11546155-A/A	1	0.1	9	0.7	0.15 (0.03–0.82)	**0.02**	**0.03**
G11	*GGT7* rs6119534-C/C × *GSS* rs6088660-C/C	236	18.3	156	13.0	1.50 (1.21–1.87)	**0.0003**	**0.001**
G12	*GGT7* rs6119534-C/C × *GSS* rs6088660-C/T	103	8.0	70	5.8	1.40 (1.03–1.92)	**0.03**	**0.04**
G13	*GGT7* rs6119534-C/T × *GSS* rs6088660-C/T	367	28.5	388	32.3	0.83 (0.70–0.99)	**0.04**	**0.042**
G14	*GGT7* rs6119534-C/T × *GSS* rs6088660-T/T	56	4.3	73	6.1	0.70 (0.49–1.00)	**0.05**	**0.05**
G15	*GGT7* rs11546155-A/A × *GSS* rs6088660-C/T	4	0.3	18	1.2	0.24 (0.09–0.68)	**0.01**	**0.02**
G16	*GGT7* rs11546155-A/A × *GSS* rs1801310-G/G	11	0.7	29	1.8	0.38 (0.19–0.77)	**0.005**	**0.01**
G17	*GGT7* rs11546155-G/G × *GSS* rs13041792-G/A	457	29.6	406	26.3	1.18 (1.00–1.38)	**0.04**	**0.042**
G18	*GGT7* rs11546155-A/A × *GSS* rs13041792-G/G	13	0.8	31	2.0	0.41 (0.22–0.79)	**0.006**	**0.01**

^1^ Odds ratio with 95% confidence intervals for a particular genotype combination (crude analysis). ^2^ *p*-values for association of particular genotype combination with T2D (Pearson’s chi-square test). ^3^ *Q*-values, adjusted *p*-values for multiple tests using false discovery rate (FDR). Bold indicates statistically significant *p*- and Q-values.

**Table 6:** Associations of the studied polymorphisms with biochemical parameters in diabetics.

Gene, SNP	Genotype	Entire Group		Males		Females	
		T2D Patients (*n* = 489)	*p*-Value * (*Q*-Value)	T2D Patients (*n* = 145)	*p*-Value * (*Q*-Value)	T2D Patients (*n* = 344)	*p*-Value * (*Q*-Value)
H_2_O_2_, µmol/L							
*GSS* rs13041792 G > A	G/G	3.67 (2.66; 4.96)	0.54 (0.72)	3.55 (2.44; 4.81)	0.17 (0.31)	3.79 (2.73; 5.02)	0.56 (0.72)
	G/A	3.77 (2.65; 5.09)		3.46 (2.39; 4.47)		3.80 (2.74; 5.10)	
	A/A	3.88 (2.76; 5.44)		3.67 (2.61; 6.94)		3.88 (2.76; 5.38)	
*GSS* rs1801310 G > A	G/G	3.56 (2.64; 4.91)	0.56 (0.72)	3.26 (2.29; 4.07)	0.26 (0.42)	3.75 (2.70; 5.04)	0.88 (0.90)
	G/A	3.80 (2.72; 5.14)		3.59 (2.42; 4.81)		3.88 (2.90; 5.17)	
	A/A	3.68 (2.46; 4.61)		4.37 (2.46; 5.33)		3.63 (2.49; 4.22)	
*GSS* rs6088660 C > T	C/C	3.77 (2.54; 4.96)	0.056 (0.18)	3.66 (2.30; 5.13)	0.73 (0.78)	3.81 (2.70; 4.94)	**0.039** (0.18)
	C/T	3.79 (2.83; 5.21)		3.76 (2.60; 5.18)		3.79 (2.90; 5.23)	
	T/T	3.37 (2.64; 4.07)		3.21 (2.41; 3.56)		3.43 (2.64; 5.87)	
*GGT7* rs11546155 G > A	G/G	3.67 (2.63; 4.96)	0.62 (0.73)	3.55 (2.42; 4.81)	0.30 (0.45)	3.72 (2.66; 4.96)	**0.046** (0.18)
	G/A	3.95 (2.74; 5.12)		3.01 (2.20; 4.24)		4.48 (3.23; 5.35)	
	A/A	3.67 (2.64; 5.04)		2.08 (2.08; 2.08)		4.15 (3.19; 5.87)	
*GGT7*rs6119534 C > T	C/C	3.33 (2.20; 4.42)	**0.0002** **(0.009)**	3.10 (2.13; 4.24)	0.05 (0.18)	3.39 (2.22; 4.82)	**0.0009** **(0.02)**
	C/T	3.74 (2.76; 5.05)		3.60 (2.42; 4.79)		3.79 (2.90; 5.09)	
	T/T	3.97 (3.05; 7.93)		3.61 (3.08; 7.93)		4.63 (2.76; 7.74)	
Total glutathione, µmol/L							
*GSS* rs13041792 G > A	G/G	1.41 (0.60; 3.67)	0.52 (0.72)	2.11 (0.91; 3.86)	0.60 (0.73)	1.34 (0.56; 3.53)	0.26 (0.42)
	G/A	2.31 (0.46; 4.01)		1.40 (0.67; 3.82)		2.42 (0.44; 4.03)	
	A/A	0.84 (0.27; 3.65)		2.56 (1.02; 4.23)		0.64 (0.27; 1.35)	
*GSS* rs1801310 G > A	G/G	2.43 (0.54; 3.92)	0.26 (0.42)	2.16 (0.77; 3.84)	0.67 (0.75)	2.44 (0.46; 4.07)	**0.039** (0.18)
	G/A	1.40 (0.58; 3.68)		1.64 (0.74; 3.75)		1.28 (0.48; 3.53)	
	A/A	1.30 (0.56; 3.18)		1.78 (0.60; 3.94)		1.29 (0.56; 2.70)	
*GSS* rs6088660 C > T	C/C	1.35 (0.56; 3.65)	0.16 (0.30)	2.20 (0.88; 3.92)	0.70 (0.77)	1.30 (0.49; 3.35)	0.086 (0.19)
	C/T	2.01 (0.53; 3.88)		1.31 (0.50; 3.67)		2.28 (0.54; 3.89)	
	T/T	2.11 (0.77; 4.21)		2.12 (1.23; 3.86)		2.93 (0.40; 4.36)	
*GGT7* rs11546155 G > A	G/G	1.47 (0.54; 3.68)	0.077 (0.18)	1.47 (0.61; 3.75)	**0.023** (0.18)	1.46 (0.51; 3.68)	0.66 (0.75)
	G/A	2.01 (0.65; 4.21)		3.55 (1.05; 4.42)		1.49 (0.39; 4.19)	
	A/A	3.11 (1.42; 4.09)		3.11 (2.45; 3.77)		2.39 (0.38; 4.40)	
*GGT7* rs6119534 C > T	C/C	1.36 (0.51; 3.34)	0.13 (0.25)	2.57 (0.88; 3.93)	0.053 (0.18)	1.28 (0.49; 2.90)	0.063 (0.18)
	C/T	1.70 (0.51; 3.89)		1.55 (0.56; 3.82)		1.93 (0.46; 3.97)	
	T/T	3.91 (0.91; 4.53)		4.70 (4.59; 3.81)		3.57 (0.59; 4.00)	
Fasting blood glucose, mmol/L							
*GSS* rs13041792 G > A	G/G	12.00 (9.40; 15.00)	**0.032** (0.18)	12.20 (9.20; 15.00)	**0.026** (0.18)	11.90 (9.40; 15.00)	0.29 (0.45)
	G/A	12.50 (10.00; 15.40)		12.50 (10.10; 15.40)		12.45 (9.92;15.35)	
	A/A	13.00 (10.20; 16.30)		13.50 (11.90; 17.60)		12.20 (9.06;16.20)	
*GSS* rs1801310 G > A	G/G	12.40 (9.50; 15.60)	0.47 (0.68)	12.50 (9.16; 15.20)	0.10 (0.20)	12.40 (9.57;16.00)	0.60 (0.73)
	G/A	12.00 (9.80; 15.00)		12.60 (10.00; 15.20)		12.00 (9.63; 14.90)	
	A/A	12.20 (9.40; 15.40)		12.50 (9.00; 15.20)		12.00 (9.45; 15.49)	
*GSS* rs6088660 C > T	C/C	12.00 (9.70; 15.20)	0.10 (0.20)	12.50 (10.10; 15.15)	**0.007** (0.11)	12.00 (9.51; 15.20)	0.78 (0.82)
	C/T	12.20 (9.60; 15.00)		12.62 (9.60; 15.40)		12.00 (9.70; 15.00)	
	T/T	11.90 (8.30; 15.50)		10.90 (7.30; 15.00)		12.60 (9.50; 16.20)	
*GGT7* rs11546155 G > A	G/G	12.20 (9.70; 15.30)	0.053 (0.18)	12.50 (9.70; 15.20)	0.06 (0.18)	12.10 (9.70; 15.30)	0.069 (0.18)
	G/A	11.60 (9.25; 14.80)		12.70 (9.20; 15.00)		11.45 (9.26; 14.60)	
	A/A	15.00 (7.50; 16.30)		13.85 (10.05; 24.95)		15.00 (7.43; 16.30)	
*GGT7* rs6119534 C > T	C/C	11.60 (9.18; 14.75)	0.07 (0.18)	12.15 (10.00; 14.65)	0.96 (0.96)	11.30 (8.83; 14.84)	**0.035** (0.18)
	C/T	12.17 (9.71; 15.00)		12.60 (9.52; 15.40)		12.00 (9.80; 15.00)	
	T/T	12.70 (10.00; 15.92)		13.10 (10.20; 15.20)		12.60 (9.60; 16.00)	

* *p*-values for the Kruskal–Wallis one-way analysis of variance. Bold indicates statistically significant *p*- and Q-values.

**Table 7:** Replication analysis for associations of *GSS* and *GGT7* gene polymorphisms with type 2 diabetes and diabetes-related phenotypes.

Gene, SNP (Effective Allele)	Phenotype	*p*-Value	Beta/Odds Ratio	Sample Size
*GSS* rs13041792 (A)	Waist–hip ratio adj BMI	0.00006	▼−0.0103	1,542,860
	BMI	0.0005	▲0.0071	3,167,810
	Non-proliferative diabetic retinopathy	0.018	▼0.9042	3045
	Waist–hip ratio	0.033	▼−0.0056	2,046,830
*GSS* rs1801310 (G)	Insulin sensitivity	0.007	▼−0.0738	2765
	Insulin sensitivity adj BMI	0.013	▼−0.0690	2764
	Diabetic retinopathy	0.014	▼0.9239	3959
	Non-proliferative diabetic retinopathy	0.018	▼0.9508	3045
	Acute insulin response adj BMI–SI	0.023	▼−0.0525	4385
	Peak insulin response adj BMI–SI	0.033	▼−0.0491	4389
	Fasting plasma glucose in diabetics and non-diabetics	0.037	▼−0.0191	16,076
	Acute insulin response adj SI	0.047	▼−0.0457	4386
*GSS* rs6088660 (T)	Obesity	0.0009	▲1.1090	11,743
	Waist circumference adj BMI–smoking status	0.02	▲0.0102	282,202
	Fasting proinsulin	0.04	▼−0.0150	10,701
*GGT7* rs11546155 (A)	Type 2 diabetes	0.018	▼0.9835	1,277,880
	Waist–hip ratio adj BMI	0.0009	▲0.0077	1,834,510
	Chronic kidney disease in type 2 diabetics	0.004	▲1.2590	300
	Fasting plasma glucose in diabetics and non-diabetics	0.016	▼−0.0275	16,076
	Neuropathy in type 2 diabetics	0.025	▲1.2462	2344
	BMI	0.027	▼−0.0062	2,814,100
	HbA1c adj BMI	0.05	▲0.0496	7267
*GGT7* rs6119534 (T)	BMI	0.0002	▲0.0069	1,335,110
	Waist–hip ratio adj BMI	0.001	▼−0.0063	971,589

Data obtained at the T2D Knowledge Portal (https://t2d.hugeamp.org), date of access, 31 January 2021; SI, insulin sensitivity; BMI, body mass index; HbA1c, glycated hemoglobin.

## Data Availability

Data supporting reported results are available upon request.

## Supplementary Materials

The following are available online at www.mdpi.com/article/10.3390/life11090886/s1. Table S1: Linkage disequilibrium (D’) between SNPs in the Russian population; Table S2: Linkage disequilibrium values (D’) between pairs of the studied SNPs across populations from the 1000 Genomes Project; Table S3: A replication analysis for SNPs of *GGT7* and *GSS* genes with significant eQTLs (skeletal muscle, GTEx portal) with the risk of T2D in the UK cohort; Table S4: Tissue-specific eQTL analysis for the polymorphisms of GSS and GGT7 genes; Table S5: QTL analysis using GTEx; Table S6: QTL analysis using QTLbase; Table S7: Pancreatic eQTLs for SNPs that are in strong linkage disequilibrium (r² > 0.8) with polymorphisms rs11546155 and rs6119534 of *GGT7* and rs13041792 of *GSS*; Table S8: Enrichment analysis for genes located at genomic cluster 20q11.22; Table S9: Predicted transcription factors co-regulating expression of *MAP1LC3A*, *MYH7B*, *CPNE1* and *EDEM2* genes; Table S10: Functional terms enriched with a set of transcription factors that have been predicted by hTFtarget as co-regulators of *EDEM2*, *MYH7B*, *MAP1LC3A* and *CPNE1* expression in the pancreas. Data S11: Tissue-specific gene expression correlated with T2D-associated alleles of GSS and GGT7 genes and disease pathogenesis. Data S12: SNPs linked to *GSS* and *GGT7* gene polymorphisms. Data S13: Environmental factors responsible for glutathione deficiency.
