# Supplementary material for: The Link between Type 2 Diabetes Mellitus and the Polymorphisms of Glutathione-Metabolizing Genes Suggests a New Hypothesis Explaining Disease Initiation and Progression
Source: Life (Basel). 2021 Aug 28;11(9):886. doi: 10.3390/life11090886 (PMC8466482; doi:10.3390/life11090886)
Supplement: Supplementary file 1 [file life-11-00886-s001.zip › Suppl Table S4.pdf]

life

MDPI

Supplementary Table S4 Tissue-specific eQTL analysis for the polymorphisms of GSS and GGT7 genes

Gene	Effect allele	SNP ID	Whole blood[1]		Pancreas[2]		Adipose tissue (visceral)[2]		Skeletal muscle[2]		Liver[2]	
			Q	*Z*	*P*	*NES*	*P*	*NES*	*P*	*NES*	*P*	*NES*
GSS	A	rs13041792	-	-	-	-	-	-	-	-	**0.009***	0.87^{β}
TRPC4AP	A	rs13041792	**<0.0001**	-58.8	-	-	**0.00032**	-0.13	-	-	**1.1×10^{-5}**	-0.30
EDEM2	A	rs13041792	**<0.0001**	-36.9	**1.3×10^{-18}**	0.48	**6×10^{-6}**	-0.16	**1.2×10^{-14}**	-0.19	-	-
MAP1LC3A	A	rs13041792	**<0.0001**	13.0	-	-	**9.9×10^{-14}**	0.25	-	-	-	-
MYH7B	A	rs13041792	**<0.0001**	12.7	**4.4×10^{-8}**	0.31	**6.3×10^{-8}**	0.21	-	-	**4.5×10^{-5}**	0.17
UQCC1	A	rs13041792	**<0.0001**	-12.2	-	-	-	-	-	-	-	-
CPNE1	A	rs13041792	**<0.0001**	-11.6	-	-	-	-	-	-	-	-
GGT7	A	rs13041792	**<0.0001**	-10.7	-	-	**0.006***	-5.16^{Z}	**7.5×10^{-10}**	0.23	-	-
EIF2S2	A	rs13041792	**<0.0001**	9.8	-	-	-	-	-	-	-	-
ITCH	A	rs13041792	**<0.0001**	9.3	-	-	-	-	-	-	-	-
PROCR	A	rs13041792	-	-	**2.8×10^{-10}**	0.61	-	-	**2.5×10^{-12}**	-0.27	**1.1×10^{-6}**	0.36
NFS1	A	rs13041792	-	-	-	-	-	-	**0.0003**	0.11	-	-
GSS	A	rs1801310	-	-	-	-	-	-	**5.6×10^{-11}**	0.20	-	-
GGT7	A	rs1801310	**<0.0001**	24.9	-	-	**0.00025**	0.092	**2.2×10^{-20}**	-0.28	-	-
TRPC4AP	A	rs1801310	**<0.0001**	-24.9	-	-	-	-	-	-	-	-
UQCC1	A	rs1801310	**<0.0001**	19.7	-	-	-	-	-	-	-	-
EIF6	A	rs1801310	**<0.0001**	-12.5	-	-	-	-	-	-	-	-
MAP1LC3A	A	rs1801310	**<0.0001**	12.3	-	-	**3.3×10^{-8}**	-0.16	**1.8×10^{-7}**	-0.15	-	-
PROCR	A	rs1801310	**<0.0001**	11.6	**2.4×10^{-6}**	-0.37	-	-	-	-	-	-

NCOA6	A	rs1801310	**<0.0001**	-11.0	-	-	-	-	**5.1×10^{-5}**	-0.096	**0.0001***	-0.16^{β}
ITCH	A	rs1801310	**<0.0001**	-10.8	-	-	-	-	-	-	-	-
MYH7B	A	rs1801310	**<0.0001**	-10.0	-	-	-	-	-	-	-	-
ACSS2	A	rs1801310	-	-	-	-	**0.0007***	3.97^{Z}	**1.2×10^{-6}**	-0.12	-	-
PIGU	A	rs1801310	-	-	-	-	-	-	**5.9×10^{-5}**	-0.1	-	-
NFS1	A	rs1801310	-	-	-	-	-	-	**0.00011**	-0.092	-	-
EDEM2	A	rs1801310	-	-	**1.5×10^{-6}**	-0.22	-	-	-	-	-	-
MMP24-AS1	A	rs1801310	-	-	**9.7×10^{-5}**	-0.21	-	-	-	-	-	-
GSS	T	rs6088660	**0.002**	5.0	-	-	-	-	**1.4×10^{-10}**	0.21	-	-
TRPC4AP	T	rs6088660	**<0.0001**	64.2	-	-	-	-	-	-	-	-
MAP1LC3A	T	rs6088660	**<0.0001**	-27.4	-	-	-	-	-	-	-	-
EDEM2	T	rs6088660	**<0.0001**	18.7	-	-	**2.6×10^{-5}**	0.13	-	-	-	-
NCOA6	T	rs6088660	**<0.0001**	12.8	-	-	-	-	**0.00022**	0.091	-	-
EIF6	T	rs6088660	**<0.0001**	-12.2	-	-	-	-	-	-	-	-
TP53INP2	T	rs6088660	**<0.0001**	-10.7	-	-	-	-	-	-	-	-
GGT7	T	rs6088660	**<0.0001**	-10.6	-	-	-	-	**1.2×10^{-6}**	0.15	-	-
MYH7B	T	rs6088660	**<0.0001**	-6.4	-	-	-	-	-	-	-	-
ACSS2	T	rs6088660	-	-	-	-	-	-	-	-	**2.5×10^{-6}***	-4.71^{Z}
GGT7	T	rs6119534	**<0.0001**	-17.7	-	-	**7.3×10^{-6}***	-4.48^{Z}	**8.7×10^{-38}**	0.37	**3.2×10^{-39}**	-13.10^{Z}
MAP1LC3A	T	rs6119534	**<0.0001**	21.6	-	-	**1.7×10^{-24}**	0.27	**1.5×10^{-7}**	0.15	**9.2×10^{-7}**	0.21
EDEM2	T	rs6119534	**<0.0001**	-18.9	**6.1×10^{-8}**	0.25	-	-	**2.3×10^{-5}**	-0.085	-	-
ITCH	T	rs6119534	**<0.0001**	14.7	-	-	-	-	-	-	-	-
EIF2S2	T	rs6119534	**<0.0001**	13.6	-	-	-	-	-	-	-	-
UQCC1	T	rs6119534	**<0.0001**	-10.5	-	-	-	-	-	-	-	-

PROCR	T	rs6119534	**<0.0001**	-9.1	**2.7×10^{-5}**	0.34	**-**	-	**1.5×10^{-5}**	-0.14	**-**	-
MYH7B	T	rs6119534	**<0.0001**	7.8	**4.3×10^{-5}**	0.19	**-**	-	**-**	-	**-**	-
ACSS2	T	rs6119534	-	-	**-**	-	**-**	-	**1.5×10^{-8}**	0.14	**6.0×10^{-8}***	-5.41^{Z}
GSS	T	rs6119534	-	-	**-**	-	**-**	-	**0.0002**	0.12	**-**	-
TRPC4AP	T	rs6119534	-	-	**-**	-	**-**	-			**3.4×10^{-7}***	-0.33^{β}
GGT7	A	rs11546155	-	-	**-**	-	**0.0001**	-0.13	**-**	-	**1.2×10^{-6}***	4.85^{Z}
MAP1LC3A	A	rs11546155	**<0.0001**	-47.7	**-**	-	**1.6×10^{-5}**	-0.16	**-**	-	**-**	-
TRPC4AP	A	rs11546155	**<0.0001**	22.2	**-**	-			**-**	-	**-**	-
NCOA6	A	rs11546155	**<0.0001**	16.9	**-**	-	**3.9×10^{-8}**	0.26	**-**	-	**-**	-
TP53INP2	A	rs11546155	**<0.0001**	-15.0	**-**	-	**-**	-	**-**	-	**-**	-
EDEM2	A	rs11546155	**<0.0001**	10.6	**-**	-	**-**	-	**-**	-	**-**	-
EIF6	A	rs11546155	**<0.0001**	-8.6	**-**	-	**-**	-	**-**	-	**-**	-
ITCH	A	rs11546155	**<0.0001**	-7.8	**-**	-	**-**	-	**-**	-	**-**	-
GSS	A	rs11546155	**0.0007**	5.2	**-**	-	**-**	-	**0.00001**	0.16	**-**	-
PIGU	A	rs11546155	-	-	**-**	-	**-**	-	**0.00001**	0.16	**-**	-

[1] Q-value (false discovery rate) and Z-score statistics for SNP's eQTLs in whole blood obtained from the eQTLGen database (https://www.eqtlgen.org).

[2] *P*-value and NES (normalized effect size) statistics for SNP's eQTLs in T2D-related tissues obtained from the GTEx portal (https://gtexportal.org).

[*] Statistics for SNP's eQTLs obtained from the QTLbase database (http://mulinlab.tmu.edu.cn/qtlbase/index.html): *P*-value and Z-score or *beta* estimate.
