# Supplementary material for: The Link between Type 2 Diabetes Mellitus and the Polymorphisms of Glutathione-Metabolizing Genes Suggests a New Hypothesis Explaining Disease Initiation and Progression
Source: Life (Basel). 2021 Aug 28;11(9):886. doi: 10.3390/life11090886 (PMC8466482; doi:10.3390/life11090886)
Supplement: Supplementary file 1 [file life-11-00886-s001.zip › Suppl Table S8.pdf]

life

MDPI

Supplementary Table S8. Enrichment analysis for genes located at genomic cluster 20q11.22.

Genes on 20q11.22 (5′→3′)	Full gene name (HGNC)	Enriched terms (Biological Process/Molecular Function/Pathways), associated adjusted P-values*
NECAB3	N-terminal EF-hand calcium binding protein 3	regulation of amyloid precursor protein biosynthetic process (GO:0042984), P=0.003<br>regulation of glycoprotein biosynthetic process (GO:0010559), P=0.003
ACTL10	actin like 10	Salivirus NG-J1 3D, Host PPI P-HIPSTer 2020, P=0.008<br>Ectromelia virus ERPV profilin-like protein, Host PPI P-HIPSTer 2020, P=0.008<br>Variola virus hypothetical protein (gene: A47R), Host PPI P-HIPSTer 2020, P=0.008<br>Horsepox virus HSPV164 (Profilin), Host PPI P-HIPSTer 2020, P=0.008<br>Monkeypox virus profilin-like protein, Host PPI P-HIPSTer 2020, P=0.008<br>Monkeypox virus Zaire-96-I-16 A42R (gene: A42R), Host PPI P-HIPSTer 2020, P=0.008<br>Cowpox virus CPXV179 protein (gene: CPXV179 CDS) , Host PPI P-HIPSTer 2020, P=0.008<br>Vaccinia virus profilin-like protein (gene: A42R), Host PPI P-HIPSTer 2020, P=0.008<br>Vaccinia virus Ankara Profilin-1-like, Host PPI P-HIPSTer 2020, P=0.008
E2F1	E2F transcription factor 1	regulation of fat cell proliferation (GO:0070344), P=0.002<br>RNA stabilization (GO:0043489), P=0.006<br>regulation of cell cycle G1/S phase transition (GO:1902806), P=0.008<br>regulation of transcription involved in G1/S transition of mitotic cell cycle (GO:0000083), P=0.009<br>positive regulation of fibroblast proliferation (GO:0048146), P=0.010<br>positive regulation of protein insertion into mitochondrial membrane involved in apoptotic signaling pathway (GO:1900740), P=0.010<br>regulation of protein insertion into mitochondrial membrane involved in apoptotic signaling pathway (GO:1900739), P=0.010<br>negative regulation of nucleic acid-templated transcription (GO:1903507), P=0.010<br>negative regulation of fat cell differentiation (GO:0045599), P=0.011<br>mRNA stabilization (GO:0048255), P=0.011<br>negative regulation of DNA binding (GO:0043392), P=0.012<br>positive regulation of mitochondrial outer membrane permeabilization involved in apoptotic signaling pathway (GO:1901030), P=0.013<br>negative regulation of G0 to G1 transition (GO:0070317), P=0.013<br>DNA damage checkpoint (GO:0000077), P=0.013<br>negative regulation of cellular macromolecule biosynthetic process (GO:2000113), P=0.013<br>regulation of G0 to G1 transition (GO:0070316), P=0.014<br>regulation of transcription, DNA-templated (GO:0006355), P=0.014<br>regulation of fibroblast proliferation (GO:0048145), P=0.016<br>regulation of G1/S transition of mitotic cell cycle (GO:2000045), P=0.016<br>intrinsic apoptotic signaling pathway in response to DNA damage (GO:0008630), P=0.017<br>regulation of nucleic acid-templated transcription (GO:1903506), P=0.017<br>regulation of DNA binding (GO:0051101), P=0.018<br>negative regulation of gene expression (GO:0010629), P=0.018<br>regulation of cellular macromolecule biosynthetic process (GO:2000112), P=0.019<br>diterpenoid metabolic process (GO:0016101), P=0.022<br>DNA damage response, signal transduction by p53 class mediator resulting in cell cycle arrest (GO:0006977), P= 0.022<br>signal transduction involved in mitotic G1 DNA damage checkpoint (GO:0072431), P=0.022<br>regulation of fat cell differentiation (GO:0045598), P=0.025<br>retinoid metabolic process (GO:0001523), P=0.026<br>nucleic acid-templated transcription (GO:0097659), P=0.026<br>negative regulation of cell cycle process (GO:0010948), P=0.028<br>positive regulation of cell cycle arrest (GO:0071158), P= 0.028<br>negative regulation of binding (GO:0051100), P=0.028<br>DNA damage response, signal transduction by p53 class mediator (GO:0030330), P=0.028<br>negative regulation of transcription, DNA-templated (GO:0045892), P=0.030<br>positive regulation of protein localization to membrane (GO:1905477), P=0.033<br>intrinsic apoptotic signaling pathway (GO:0097193), P=0.035<br>G1/S transition of mitotic cell cycle (GO:0000082), P=0.036<br>positive regulation of establishment of protein localization to mitochondrion (GO:1903749), P=0.038<br>regulation of mRNA stability (GO:0043488), P=0.044<br>regulation of gene expression (GO:0010468), P=0.047<br>retinol binding (GO:0019841), P=0.002<br>retinoid binding (GO:0005501), P=0.008

		core promoter binding (GO:0001047), P=0.033<br>DNA binding (GO:0003677), P=0.036
PXMP4	peroxisomal membrane protein 4	No enriched terms
ZNF341	zinc finger protein 341	regulation of transcription, DNA-templated (GO:0006355), P=0.014<br>regulation of nucleic acid-templated transcription (GO:1903506), P=0.017<br>regulation of cellular macromolecule biosynthetic process (GO:2000112), P=0.019<br>regulation of gene expression (GO:0010468), P=0.047<br>DNA binding (GO:0003677), P=0.036
ZNF341-AS1	ZNF341 antisense RNA 1	No enriched terms
CHMP4B	charged multivesicular body protein 4B	posttranslational protein targeting to endoplasmic reticulum membrane (GO:0006620), P=0.002<br>negative regulation of autophagosome assembly (GO:1902902), P=0.002<br>regulation of symbiosis, encompassing mutualism through parasitism (GO:0043903), P=0.003<br>exit from mitosis (GO:0010458), P=0.003<br>positive regulation of viral release from host cell (GO:1902188), P=0.004<br>virion assembly (GO:0019068), P=0.004<br>nuclear envelope reassembly (GO:0031468), P=0.004<br>regulation of viral process (GO:0050792), P=0.004<br>regulation of mitotic spindle assembly (GO:1901673), P=0.005<br>viral budding via host ESCRT complex (GO:0039702), P=0.005<br>negative regulation of macroautophagy (GO:0016242), P=0.005<br>viral budding (GO:0046755), P=0.006<br>regulation of centrosome duplication (GO:0010824), P=0.006<br>regulation of spindle assembly (GO:0090169), P=0.006<br>regulation of viral release from host cell (GO:1902186), P=0.006<br>regulation of centrosome cycle (GO:0046605), P=0.006<br>negative regulation of organelle assembly (GO:1902116), P=0.007<br>multivesicular body assembly (GO:0036258), P=0.007<br>nuclear envelope organization (GO:0006998), P=0.007<br>multivesicular body organization (GO:0036257), P=0.008<br>regulation of autophagosome assembly (GO:2000785), P=0.008<br>positive regulation of locomotion (GO:0040017), P=0.008<br>regulation of mitotic spindle organization (GO:0060236), P=0.009<br>mitotic metaphase plate congression (GO:0007080), P=0.011<br>metaphase plate congression (GO:0051310), P=0.011<br>positive regulation of viral life cycle (GO:1903902), P=0.011<br>nucleus organization (GO:0006997), P=0.011<br>mitotic cytokinesis (GO:0000281), P=0.015<br>protein targeting to membrane (GO:0006612), P=0.015<br>cytoskeleton-dependent cytokinesis (GO:0061640), P=0.017<br>regulation of neuron death (GO:1901214), P=0.018<br>mitotic nuclear division (GO:0140014), P=0.018<br>negative regulation of cell death (GO:0060548), P=0.020<br>mitotic sister chromatid segregation (GO:0000070), P=0.020<br>negative regulation of neuron death (GO:1901215), P=0.021<br>regulation of mitotic nuclear division (GO:0007088), P=0.021<br>proteolysis involved in cellular protein catabolic process (GO:0051603), P=0.023<br>regulation of cell death (GO:0010941), P=0.024<br>protein targeting to ER (GO:0045047), P=0.024<br>viral life cycle (GO:0019058), P=0.026<br>macroautophagy (GO:0016236), P=0.027<br>protein homooligomerization (GO:0051260), P=0.047<br>Budding and maturation of HIV virion Homo sapiens R-HSA-162588, P=(Reactome) 0.006<br>Endosomal Sorting Complex Required For Transport (ESCRT) Homo sapiens R-HSA-917729 (Reactome), P=0.007<br>Macroautophagy Homo sapiens R-HSA-1632852 (Reactome) , P=0.017<br>Late Phase of HIV Life Cycle Homo sapiens R-HSA-162599, P= (Reactome) 0.032<br>HIV Life Cycle Homo sapiens R-HSA-162587 (Reactome), P=0.035
RALY	RALY heterogeneous nuclear ribonucleoprotein	regulation of RNA biosynthetic process (GO:2001141), P=0.002<br>regulation of cholesterol homeostasis (GO:2000188), P= 0.003<br>RNA binding (GO:0003723), P=0.042
RALY-AS1	RALY antisense RNA 1	No enriched terms

MIR4755	microRNA 4755	No enriched terms
EIF2S2	eukaryotic translation initiation factor 2 subunit beta	translation initiation factor activity (GO:0003743), P=0.009<br>translation factor activity, RNA binding (GO:0008135), P=0.017<br>RNA binding (GO:0003723), P=0.042<br>Regulation of eIF2 Homo sapiens h eif2Pathway (BioCarta), P=0.003<br>Double Stranded RNA Induced Gene Expression Homo sapiens h rnaPathway (BioCarta), P=0.003<br>Eukaryotic protein translation Homo sapiens h eifPathway (BioCarta), P=0.003<br>Skeletal muscle hypertrophy is regulated via AKT/mTOR pathway Homo sapiens h igf1mtorpathway (BioCarta), P=0.006<br>Recycling of eIF2:GDP Homo sapiens R-HSA-72731 (Reactome), P=0.002<br>ABC-family proteins mediated transport Homo sapiens R-HSA-382556 (Reactome), P=0.011<br>Formation of the ternary complex, and subsequently, the 43S complex Homo sapiens R-HSA-72695 (Reactome), P=0.012<br>Ribosomal scanning and start codon recognition Homo sapiens R-HSA-72702 (Reactome), P=0.014<br>Translation initiation complex formation Homo sapiens R-HSA-72649 (Reactome), P=0.014<br>Activation of the mRNA upon binding of the cap-binding complex and eIFs, and subsequent binding to 43S Homo sapiens R-HSA-72662 (Reactome), P=0.014<br>L13a-mediated translational silencing of Ceruloplasmin expression Homo sapiens R-HSA-156827 (Reactome), P=0.026<br>3' -UTR-mediated translational regulation Homo sapiens R-HSA-157279 (Reactome), P=0.026<br>GTP hydrolysis and joining of the 60S ribosomal subunit Homo sapiens R-HSA-72706 (Reactome), P=0.026<br>Cap-dependent Translation Initiation Homo sapiens R-HSA-72737 (Reactome), P=0.028<br>Eukaryotic Translation Initiation Homo sapiens R-HSA-72613 (Reactome), P=0.028<br>Translation Homo sapiens R-HSA-72766 (Reactome), P=0.037
ASIP	agouti signaling protein	Adipogenesis (WP236), P=0.045<br>Proteins Involved in Diabetes Mellitus Type 2 (EPC), P=0.040<br>Insulin regulation of blood glucose (EPC), P=0.009<br>Melanogenesis, P= (BP), P=0.035<br>Adipogenesis, P= (BP), P=0.046<br>Melanogenesis (KEGG), P=0.035<br>Insulin-mediated glucose transport Homo sapiens 145e3376-6194-11e5-8ac5-06603eb7f303 (NCI-Nature), P=0.009
AHCY	adenosylhomocysteinase	L-cysteine metabolic process (GO:0046439), P=0.002<br>methionine metabolic process (GO:0006555), P=0.004<br>sulfur amino acid metabolic process (GO:0000096), P= 0.008<br>Trans-sulfuration pathway WP2333, P=0.003<br>Methionine metabolism leading to Sulphur Amino Acids and related disorders (WP4292), P=0.004<br>Methionine De Novo and Salvage Pathway (WP3580), P=0.008<br>One Carbon Metabolism (WP241), P=0.010<br>Trans-sulfuration and one carbon metabolism (WP2525), P=0.011<br>Ethanol effects on histone modifications (WP3996), P=0.011<br>Sleep regulation (WP3591) P=0.013<br>Folate Metabolism (WP176), P=0.023<br>Methylation (BP), P= 0.004<br>Sulfur amino acid metabolism (BP), P=0.008<br>Selenoamino acid metabolism (BP), P=0.009<br>One-carbon metabolism (BP), P=0.011<br>Cysteine and methionine metabolism (BP), P=0.012<br>Folate metabolism (BP), P=0.022<br>Phase II of biological oxidations: conjugation (BP), P=0.025<br>Biological oxidations (BP), P=0.048<br>Cysteine and methionine metabolism (KEGG), P=0.016<br>cysteine biosynthesis Homo sapiens PWY-6292 (HumanCyc) , P=0.002<br>superpathway of methionine degradation Homo sapiens PWY-5328 (HumanCyc), P=0.006<br>Metabolism of ingested SeMet, Sec, MeSec into H2Se Homo sapiens R-HSA-2408508 (Reactome), P=0.003<br>Methylation Homo sapiens R-HSA-156581 (Reactome), P=0.005<br>Sulfur amino acid metabolism Homo sapiens R-HSA-1614635 (Reactome), P=0.009<br>Phase II conjugation Homo sapiens R-HSA-156580 (Reactome), P=0.034<br>Selenoamino acid metabolism Homo sapiens R-HSA-2408522 (Reactome), P=0.038
ITCH	itchy E3 ubiquitin protein ligase	regulation of protein deubiquitination (GO:0090085), P=0.002<br>negative regulation of defense response to virus (GO:0050687), P=0.004<br>regulation of defense response to virus (GO:0050688), P=0.005<br>regulation of protein modification by small protein conjugation or removal (GO:1903320), P=0.005<br>negative regulation of immune effector process (GO:0002698), P=0.006

viral entry into host cell (GO:0046718), P=0.006
negative regulation of response to biotic stimulus (GO:0002832), P=0.007
cytoplasmic pattern recognition receptor signaling pathway (GO:0002753), P=0.008
nucleotide-binding domain, leucine rich repeat containing receptor signaling pathway (GO:0035872), P=0.009
negative regulation of JNK cascade (GO:0046329), P=0.010
nucleotide-binding oligomerization domain containing signaling pathway (GO:0070423), P=0.010
negative regulation of stress-activated MAPK cascade (GO:0032873), P=0.010
negative regulation of multi-organism process (GO:0043901), P=0.011
entry into host cell (GO:0030260), P=0.011
protein K63-linked ubiquitination (GO:0070534), P=0.013
negative regulation of type I interferon production (GO:0032480), P=0.015
protein K48-linked ubiquitination (GO:0070936), P=0.017
protein autoubiquitination (GO:0051865), P=0.017
Notch signaling pathway (GO:0007219), P=0.019
positive regulation of catabolic process (GO:0009896), P=0.022
regulation of proteolysis (GO:0030162), P=0.022
protein ubiquitination involved in ubiquitin-dependent protein catabolic process (GO:0042787), P=0.024
regulation of hematopoietic stem cell differentiation (GO:1902036), P=0.026
regulation of hematopoietic progenitor cell differentiation (GO:1901532), P=0.027
negative regulation of defense response (GO:0031348), P=0.028
positive regulation of protein catabolic process (GO:0045732), P=0.028
negative regulation of NF-kappaB transcription factor activity (GO:0032088), P=0.028
regulation of type I interferon production (GO:0032479), P=0.029
regulation of stem cell differentiation (GO:2000736), P=0.030
regulation of JNK cascade (GO:0046328), P=0.031
regulation of protein catabolic process (GO:0042176), P=0.031
positive regulation of protein metabolic process (GO:0051247), P=0.033
positive regulation of cellular catabolic process (GO:0031331), P=0.037
negative regulation of response to external stimulus (GO:0032102), P=0.037
viral life cycle (GO:0019058), P=0.037
negative regulation of cytokine production (GO:0001818), P=0.038
regulation of cellular component organization (GO:0051128), P=0.048
negative regulation of sequence-specific DNA binding transcription factor activity (GO:0043433), P= 0.048
modification-dependent protein catabolic process (GO:0019941), P=0.048
Notch Signaling Pathway (WP61), P=0.021
TGF-beta Signaling Pathway (WP366), P=0.045
Hippo/YAP1 Signaling (EPC), P=0.011
Downregulation of ERBB4 signaling (BP), P=0.003
Activated NOTCH1 signaling in the nucleus (BP), P=0.011
Negative regulators of RIG-I/MDA5 signaling (BP), P=0.011
ERBB4 signaling events (BP), P=0.013
Nucleotide-binding domain, leucine rich repeat containing receptor (NLR) signaling pathways (BP), P=0.016
Regulation of NFAT transcription factors (BP), P=0.016
Delta Np63 pathway (BP), P=0.016
TAp63 pathway, P=(BP), P=0.019
Signaling by NOTCH1 (BP), P=0.025
RIG-I/MDA5-mediated induction of interferon-alpha/beta pathways (BP), P=0.026
p73 transcription factor network (BP), P=0.027
NOD signaling pathway (BP), P=0.029
Signaling by ERBB4 (BP), P=0.032
CXCR4 signaling pathway (BP), P=0.040
Signaling by NOTCH (BP), P=0.041
Notch signaling pathway, P=(BP), P=0.042
Ubiquitin-mediated proteolysis (BP), P=0.047
EGF/EGFR signaling pathway (BP), P=0.048
TGF-beta signaling pathway (BP), P=0.002
TNF signaling pathway (KEGG), P=0.038
Ubiquitin mediated proteolysis (KEGG), P=0.047
TGF-beta receptor signaling Homo sapiens 1f188fcc-6196-11e5-8ac5-06603eb7f303 (NCI-Nature), P=0.0001
ErbB4 signaling events Homo sapiens 6104ebb2-6192-11e5-8ac5-06603eb7f303 (NCI-Nature), P=0.013
Validated transcriptional targets of deltaNp63 isoforms Homo sapiens 7d7360a8-6196-11e5-8ac5-06603eb7f303, P=(NCI-Nature) 0.016
Calcineurin-regulated NFAT-dependent transcription in lymphocytes Homo sapiens 0439e9da-618f-11e5-8ac5-06603eb7f303 (NCI-Nature), P=0.016

		Validated transcriptional targets of TAp63 isoforms Homo sapiens 82e8ee19-6196-11e5-8ac5-06603eb7f303 (NCI-Nature), P=0.018<br>Notch signaling pathway Homo sapiens 88f83518-6194-11e5-8ac5-06603eb7f303 (NCI-Nature), P=0.020<br>p73 transcription factor network Homo sapiens a88c505e-6194-11e5-8ac5-06603eb7f303 (NCI-Nature), P=0.027<br>CXCR4-mediated signaling events Homo sapiens 46a5529b-6191-11e5-8ac5-06603eb7f303 (NCI-Nature), P=0.034
MIR644A	microRNA 644a	No enriched terms
DYNLRB1	dynein light chain roadblock-type 1	TGF-beta receptor signaling Homo sapiens 1f188fcc-6196-11e5-8ac5-06603eb7f303 (NCI-Nature), P=0.0001<br>intraciliary transport involved in cilium assembly (GO:0035735), P=0.014<br>intraciliary transport (GO:0042073), P=0.016<br>Intraflagellar transport proteins binding to dynein (WP4532), P=0.009<br>TGF-beta signaling pathway (BP), P=0.002<br>Intraflagellar transport Homo sapiens R-HSA-5620924 (Reactome), P=0.014
MAP1LC3A	microtubule associated protein 1 light chain 3 alpha	cellular response to nitrogen levels (GO:0043562), P=0.005<br>cellular response to nitrogen starvation (GO:0006995), P=0.005<br>autophagosome maturation (GO:0097352), P=0.008<br>mitochondrion disassembly (GO:0061726), P=0.013<br>autophagy of mitochondrion (GO:0000422), P=0.014<br>protein complex disassembly (GO:0043241), P=0.016<br>cellular response to nutrient levels (GO:0031669), P=0.019<br>autophagosome organization (GO:1905037), P=0.019<br>autophagosome assembly (GO:0000045), P=0.020<br>cellular response to starvation (GO:0009267), P=0.037<br>macroautophagy (GO:0016236), P=0.038<br>Nanoparticle triggered autophagic cell death (WP2509), P=0.008<br>Factors and pathways affecting insulin-like growth factor (IGF1)-Akt signaling (WP3850), P=0.011<br>Ferroptosis (WP4313), P=0.014<br>Senescence and Autophagy in Cancer (WP615), P=0.036<br>beta-Cell Function Inhibition by Rapamycin, Rodent Model, P=(EPC) 0.003<br>OPTN Associated Glaucoma (EPC), P=0.008<br>Autophagic-Lysosomal System Decline Associated with Aging (EPC), P=0.010<br>Senescence and autophagy (BP), P=0.034<br>Ferroptosis (KEGG), P=0.014<br>Downregulation of ERBB4 signaling Homo sapiens R-HSA-1253288 (Reactome), P= 0.003<br>Activated NOTCH1 Transmits Signal to the Nucleus Homo sapiens R-HSA-2122948 (Reactome), P=0.010<br>NOD1/2 Signaling Pathway Homo sapiens R-HSA-168638 (Reactome), P=0.011<br>Negative regulators of RIG-I/MDA5 signaling Homo sapiens R-HSA-936440, P=(Reactome) 0.012<br>Nucleotide-binding domain, leucine rich repeat containing receptor (NLR) signaling pathways Homo sapiens R-HSA-168643 (Reactome), P=0.016<br>Degradation of GLI1 by the proteasome Homo sapiens R-HSA-5610780 (Reactome), P=0.020<br>Signaling by NOTCH1 Homo sapiens R-HSA-1980143 (Reactome), P=0.025<br>RIG-I/MDA5 mediated induction of IFN-alpha/beta pathways Homo sapiens R-HSA-168928 (Reactome), P=0.027<br>Hedgehog 'on' state Homo sapiens R-HSA-5632684 (Reactome), P= 0.029<br>Hedgehog 'off' state Homo sapiens R-HSA-5610787 (Reactome), P= 0.034<br>Signaling by NOTCH Homo sapiens R-HSA-157118 (Reactome), P= 0.042<br>Signaling by Hedgehog Homo sapiens R-HSA-5358351 (Reactome), P= 0.047
PIGU	phosphatidylinositol glycan anchor biosynthesis class U	attachment of GPI anchor to protein (GO:0016255), P=0.002<br>protein localization to cell surface (GO:0034394), P=0.008<br>GPI anchor metabolic process (GO:0006505), P=0.009<br>GPI anchor biosynthetic process (GO:0006506), P=0.011<br>glycolipid biosynthetic process (GO:0009247), P=0.015<br>regulation of JAK-STAT cascade (GO:0046425), P=0.023<br>phosphatidylinositol biosynthetic process (GO:0006661), P=0.034<br>protein lipidation (GO:0006497), P=0.044<br>Attachment of GPI anchor to uPAR (BP), P=0.002<br>Post-translational modification: biosynthesis of GPI-anchored proteins , P=(BP), P=0.010<br>Glycosylphosphatidylinositol (GPI)-anchor biosynthesis (KEGG), P=0.009<br>Attachment of GPI anchor to uPAR Homo sapiens R-HSA-162791 (Reactome), P= 0.002<br>Post-translational modification: synthesis of GPI-anchored proteins Homo sapiens R-HSA-163125 (Reactome), P=0.009
TP53INP2	tumor protein p53 inducible nuclear protein 2	positive regulation of nucleic acid-templated transcription (GO:1903508) , P=0.025<br>autophagosome organization (GO:1905037), P= 0.028<br>autophagosome assembly (GO:0000045), P=0.029

NCOA6	nuclear receptor coactivator 6	glucocorticoid receptor signaling pathway (GO:0042921), P=0.003<br>corticosteroid receptor signaling pathway (GO:0031958), P=0.003<br>intracellular estrogen receptor signaling pathway (GO:0030520), P=0.009<br>RNA biosynthetic process (GO:0032774), P=0.013<br>myeloid cell differentiation (GO:0030099), P=0.022<br>DNA recombination (GO:0006310), P=0.027<br>positive regulation of nucleic acid-templated transcription (GO:1903508) , P=0.025<br>intracellular steroid hormone receptor signaling pathway (GO:0030518), P=0.033<br>hemopoiesis (GO:0030097), P=0.037<br>regulation of lipid metabolic process (GO:0019216), P=0.049<br>retinoid X receptor binding (GO:0046965), P=0.004<br>retinoic acid receptor binding (GO:0042974), P=0.008<br>thyroid hormone receptor binding (GO:0046966), P=0.013<br>estrogen receptor binding (GO:0030331), P=0.016<br>hormone receptor binding (GO:0051427), P=0.020<br>nuclear hormone receptor binding (GO:0035257), P=0.028<br>ligand-dependent nuclear receptor transcription coactivator activity (GO:0030374), P=0.032<br>steroid hormone receptor binding (GO:0035258), P=0.037<br>Constitutive Androstane Receptor Pathway (WP2875), P=0.016<br>Metabolism Homo sapiens R-HSA-1430728 (Reactome), P=0.011<br>RORA activates gene expression Homo sapiens R-HSA-1368082 (Reactome), P=0.013<br>YAP1- and WWTR1 (TAZ)-stimulated gene expression Homo sapiens R-HSA-2032785 (Reactome), P=0.014<br>Transcriptional activation of mitochondrial biogenesis Homo sapiens R-HSA-2151201 (Reactome), P=0.020<br>BMAL1:CLOCK,NPAS2 activates circadian gene expression Homo sapiens R-HSA-1368108 (Reactome), P=0.021<br>Activation of gene expression by SREBF (SREBP) Homo sapiens R-HSA-2426168 (Reactome), P=0.021<br>Mitochondrial biogenesis Homo sapiens R-HSA-1592230 (Reactome), P=0.024<br>Regulation of cholesterol biosynthesis by SREBP (SREBF) Homo sapiens R-HSA-1655829 (Reactome), P=0.027<br>Circadian Clock Homo sapiens R-HSA-400253 (Reactome), P=0.031<br>Transcriptional regulation of white adipocyte differentiation Homo sapiens R-HSA-381340 (Reactome), P=0.039<br>Activation of HOX genes during differentiation Homo sapiens R-HSA-5619507 (Reactome), P=0.044<br>Activation of anterior HOX genes in hindbrain development during early embryogenesis Homo sapiens R-HSA-5617472 (Reactome), P=0.044
HMGB3P1	high mobility group box 3 pseudogene 1	No enriched terms
GGT7	gamma-glutamyltransferase 7	sulfur compound biosynthetic process (GO:0044272), P=0.002<br>regulation of response to oxidative stress (GO:1902882), P=0.004<br>leukotriene D4 biosynthetic process (GO:1901750), P= 0.005<br>leukotriene D4 metabolic process (GO:1901748), P=0.005<br>leukotriene biosynthetic process (GO:0019370), P=0.009<br>Metabolism Homo sapiens R-HSA-1430728 (Reactome), P=0.011<br>Ethanol oxidation Homo sapiens R-HSA-71384 (Reactome), P=0.006<br>Aflatoxin activation and detoxification Homo sapiens R-HSA-5423646 (Reactome), P=0.010<br>Glutathione synthesis and recycling Homo sapiens R-HSA-174403 (Reactome), P=0.00002<br>Glutathione conjugation Homo sapiens R-HSA-156590 (Reactome), P=0.0002<br>Phase II conjugation Homo sapiens R-HSA-156580 (Reactome), P=0.001
ACSS2	acyl-CoA synthetase short chain family member 2	CoA-ligase activity (GO:0016405), P=0.005<br>acid-thiol ligase activity (GO:0016878), P=0.008<br>adenyl ribonucleotide binding (GO:0032559), P=0.008<br>lipid biosynthetic process (GO:0008610), P=0.035<br>Fatty Acid Biosynthesis (WP357), P=0.011<br>Lipid Metabolism Pathway (WP3965), P=0.014<br>Ethanol effects on histone modifications (WP3996), P=0.015<br>Fatty Acid Beta Oxidation (WP143), P=0.017<br>ethanol degradation II Homo sapiens PWY66-21 (HumanCyc), P=0.003<br>ethanol degradation IV Homo sapiens PWY66-162, P=0.003<br>oxidative ethanol degradation III Homo sapiens PWY66-161, P=0.003<br>Biological oxidations Homo sapiens R-HSA-211859, P= (Reactome) 0.0001<br>Metabolism Homo sapiens R-HSA-1430728 (Reactome), P=0.011<br>Ethanol oxidation Homo sapiens R-HSA-71384 (Reactome), P=0.006<br>Phase 1 - Functionalization of compounds Homo sapiens R-HSA-211945 (Reactome), P=0.045
GSS	glutathione synthetase	sulfur compound biosynthetic process (GO:0044272), P=0.002<br>glutathione biosynthetic process (GO:0006750), P=0.005<br>nonribosomal peptide biosynthetic process (GO:0019184), P=0.006

		cellular modified amino acid biosynthetic process (GO:0042398), P=0.012<br>glutathione metabolic process (GO:0006749), P=0.024<br>acid-amino acid ligase activity (GO:0016881), P=0.007<br>adenyl ribonucleotide binding (GO:0032559), P=0.008<br>Gamma-Glutamyl Cycle for the biosynthesis and degradation of glutathione, including diseases (WP4518), P=0.005<br>Cysteine and methionine catabolism (WP4504), P=0.007<br>Glutathione metabolism (WP100), P=0.011<br>Trans-sulfuration and one carbon metabolism (WP2525), P=0.015<br>Ferroptosis (WP4313), P=0.020<br>One carbon metabolism and related pathways (WP3940), P=0.026<br>Amino Acid metabolism (WP3925, P=) 0.045<br>γ-glutamyl cycle Homo sapiens PWY-4041 (HumanCyc), P=0.004<br>Metabolism Homo sapiens R-HSA-1430728 (Reactome), P=0.011<br>Ethanol oxidation Homo sapiens R-HSA-71384 (Reactome), P=0.006<br>Glutathione synthesis and recycling Homo sapiens R-HSA-174403 (Reactome), P=0.00002<br>Glutathione conjugation Homo sapiens R-HSA-156590 (Reactome), P=0.0002<br>Phase II conjugation Homo sapiens R-HSA-156580 (Reactome), P=0.001
TRPC4AP	tumor protein p53 inducible nuclear protein 2	calcium ion transmembrane transporter activity (GO:0015085), P=0.038<br>calcium ion transmembrane transport (GO:0070588), P= 0.042<br>calcium channel activity (GO:0005262), P=0.046<br>phosphatase binding (GO:0019902), P=0.050<br>TRP channels Homo sapiens R-HSA-3295583 (Reactome), P=0.012<br>Stimuli-sensing channels Homo sapiens R-HSA-2672351 (Reactome), P=0.049
MYH7B	myosin heavy chain 7B	Nicotinic acetylcholine receptor signaling pathway Homo sapiens (P00044), P=0.03<br>Cytoskeletal regulation by Rho GTPase Homo sapiens (P00016), P=0.03<br>Translocation of GLUT4 to the plasma membrane Homo sapiens R-HSA-1445148 (Reactome), P=0.030
MIR499A	microRNA 499a	No enriched terms
MIR499B	microRNA 499b	No enriched terms
MMP24-AS1-EDEM2	matrix metallopeptidase 24 antisense RNA 1-ER degradation enhancing alpha-mannosidase like protein 2	No enriched terms
EDEM2	ER degradation enhancing alpha-mannosidase like protein 2	endoplasmic reticulum quality control compartment (GO:0044322), P=0.004<br>positive regulation of protein exit from endoplasmic reticulum (GO:0070863), P=0.005<br>mitochondrial respiratory chain complex III biogenesis (GO:0097033), P=0.005<br>regulation of retrograde protein transport, ER to cytosol (GO:1904152), P=0.006<br>positive regulation of ERAD pathway (GO:1904294), P=0.006<br>maturation of 5.8S rRNA (GO:0000460), P=0.008<br>endoplasmic reticulum mannose trimming (GO:1904380), P=0.009<br>protein alpha-1,2-demannosylation (GO:0036508), P=0.012<br>glycoprotein metabolic process (GO:0009100), P=0.024<br>ubiquitin-dependent ERAD pathway (GO:0030433), P=0.029<br>Wolfram Syndrome Progression, Hypothesis (EPC), P=0.009<br>SERPINA1 Associated Liver Damage (EPC), P=0.012<br>Nitric Oxide Effects on beta-Cell (EPC), P=0.013<br>MYOC Associated Glaucoma (EPC), P=0.014<br>Hyperglycemia and Hyperlipidemia Trigger beta-Cell Apoptosis (EPC), P=0.014<br>CFTR Misfolding and Degradation (Class II Mutations) (EPC), P=0.015<br>ER Stress (Unfolded Protein Response) (EPC), P=0.015<br>ER quality control compartment, ERQC (BP), P=0.003<br>N-glycan trimming in the ER and calnexin/calreticulin cycle (BP), P=0.006<br>Asparagine N-linked glycosylation , P=(BP), P=0.04
PROCR	protein C receptor	glycoprotein catabolic process (GO:0006516), P=0.005<br>negative regulation of coagulation (GO:0050819), P=0.007<br>Hematopoietic Cell Lineage: B-cell (EPC), P=0.019<br>Arterial Hypertension (EPC), P=0.022<br>Vascular Endothelial Cell Activation by Growth Factors (EPC), P=0.025<br>Vascular Endothelial Cell Activation by Blood Coagulation Factors (EPC), P=0.028<br>Vascular Endothelial Cell Activation by Cytokines (EPC), P=0.033

MMP24	matrix metallopeptidase 24	trans-Golgi network membrane (GO:0032588), P=0.042<br>regulation of fatty acid metabolic process (GO:0019217), P=0.007<br>cell-cell adhesion mediated by cadherin (GO:0044331), P=0.011<br>sensory perception of pain (GO:0019233), P=0.012<br>glial cell differentiation (GO:0010001), P=0.012<br>Matrix metalloproteinases, P= (BP) 0.014<br>Activation of matrix metalloproteinases (BP), P=0.015<br>Extracellular matrix organization (BP), P=0.046
MMP24OS	MMP24 opposite strand	unknown function
EIF6	eukaryotic translation initiation factor 6	preribosome, large subunit precursor (GO:0030687), P=0.013<br>preribosome (GO:0030684), P=0.037<br>positive regulation of translation (GO:0045727), P=0.001<br>rRNA-containing ribonucleoprotein complex export from nucleus (GO:0071428), P=0.003<br>miRNA mediated inhibition of translation (GO:0035278), P=0.005<br>negative regulation of translation, ncRNA-mediated (GO:0040033), P=0.005<br>ribosomal subunit export from nucleus (GO:0000054), P=0.006<br>gliogenesis (GO:0042063), P=0.007<br>N-glycan processing (GO:0006491), P=0.008<br>posttranscriptional gene silencing by RNA (GO:0035194), P=0.009<br>maturation of LSU-rRNA (GO:0000470), P=0.011<br>regulation of fatty acid biosynthetic process (GO:0042304), P=0.014<br>regulation of lipid biosynthetic process (GO:0046890), P=0.014<br>regulation of cellular biosynthetic process (GO:0031326), P=0.014<br>gene silencing by miRNA (GO:0035195), P=0.015<br>regulation of carbohydrate catabolic process (GO:0043470), P=0.018<br>regulation of coenzyme metabolic process (GO:0051196), P=0.019<br>response to peptide hormone (GO:0043434), P=0.020<br>regulation of cellular metabolic process (GO:0031323), P=0.020<br>regulation of ATP metabolic process (GO:1903578), P=0.021<br>regulation of generation of precursor metabolites and energy (GO:0043467), P=0.023<br>regulation of megakaryocyte differentiation (GO:0045652), P=0.024<br>regulation of reactive oxygen species metabolic process (GO:2000377), P=0.024<br>ribosome assembly (GO:0042255), P=0.028<br>regulation of glycolytic process (GO:0006110), P=0.028<br>positive regulation of cellular amide metabolic process (GO:0034250), P=0.030<br>ribosomal large subunit biogenesis (GO:0042273), P=0.031<br>regulation of myeloid cell differentiation (GO:0045637), P=0.032<br>response to insulin (GO:0032868), P=0.035<br>negative regulation of translation (GO:0017148), P=0.043<br>positive regulation of cellular protein metabolic process (GO:0032270), P=0.045<br>Eukaryotic protein translation (BP) , P=0.008<br>Translation factors (BP), P=0.025
FAM83C-AS1	FAM83C antisense RNA 1	No enriched terms
FAM83C	family with sequence similarity 83 member C	No enriched terms
UQCC1	ubiquinol-cytochrome c reductase complex assembly factor 1	positive regulation of translation (GO:0045727), P=0.001<br>mitochondrial respiratory chain complex III assembly (GO:0034551), P=0.004<br>positive regulation of mitochondrial translation (GO:0070131), P=0.004<br>respiratory chain complex III assembly (GO:0017062), P=0.004<br>regulation of coagulation (GO:0050818), P=0.005<br>regulation of mitochondrial translation (GO:0070129), P=0.007<br>mitochondrial respiratory chain complex assembly (GO:0033108), P=0.047
MIR1289-1	microRNA 1289-1	No enriched terms
GDF5OS	GDF5 antisense RNA 1	No enriched terms
GDF5	growth differentiation factor 5	Waist-to-hip ratio adjusted for BMI (GWAS catalog), P=3.1E-08<br>Waist-to-hip ratio adjusted for BMI, age >50 (GWAS catalog), P=3.3E-11<br>Waist-to-hip ratio adjusted for BMI x sex x age interaction, 4df test (GWAS catalog), P=1.2E-09<br>Waist-to-hip ratio adjusted for BMI in active individuals, P=2.7E-02<br>Waist-to-hip ratio adjusted for BMI (joint analysis for main effect and physical activity interaction), P=3.3E-02

CEP250	centrosomal protein 250	Waist-to-hip ratio adjusted for BMI (GWAS catalog), P=3.1E-08<br>Waist-to-hip ratio adjusted for BMI, age >50 (GWAS catalog), P=3.3E-11<br>Waist-to-hip ratio adjusted for BMI x sex x age interaction, 4df test (GWAS catalog), P=1.2E-09<br>NRF1 ENCODE, P=0.021
ERGIC3	ERGIC and golgi 3	Waist-to-hip ratio adjusted for BMI (GWAS catalog), P=3.1E-08<br>Waist-to-hip ratio adjusted for BMI, age >50 (GWAS catalog), P=3.3E-11<br>Waist-to-hip ratio adjusted for BMI x sex x age interaction, 4df test (GWAS catalog), P=1.2E-09<br>NRF1 ENCODE, P=0.021
FER1L4	fer-1 like family member 4 (pseudogene)	Waist-to-hip ratio adjusted for BMI (GWAS catalog), P=3.1E-08<br>Waist-to-hip ratio adjusted for BMI, age >50 (GWAS catalog), P=3.3E-11<br>Waist-to-hip ratio adjusted for BMI x sex x age interaction, 4df test (GWAS catalog), P=1.2E-09
SPAG4	sperm associated antigen 4	Waist-to-hip ratio adjusted for BMI (GWAS catalog), P=3.1E-08<br>Waist-to-hip ratio adjusted for BMI, age >50 (GWAS catalog), P=3.3E-11<br>Waist-to-hip ratio adjusted for BMI x sex x age interaction, 4df test (GWAS catalog), P=1.2E-09<br>CREB1 CHEA, P=0.05<br>ATF2 ENCODE, P=0.019
CPNE1	copine 1	Waist-to-hip ratio adjusted for BMI (GWAS catalog), P=3.1E-08<br>Waist-to-hip ratio adjusted for BMI, age >50 (GWAS catalog), P=3.3E-11<br>Waist-to-hip ratio adjusted for BMI x sex x age interaction, 4df test (GWAS catalog), P=1.2E-09<br>NRF1 ENCODE, P=0.021<br>E2F1 CHEA, P=0.013<br>ATF2 ENCODE, P=0.019
RBM12	RNA binding motif protein 12	Waist-to-hip ratio adjusted for BMI, age >50 (GWAS catalog), P=3.3E-11<br>Waist-to-hip ratio adjusted for BMI x sex x age interaction, 4df test (GWAS catalog), P=1.2E-09<br>NRF1 ENCODE, P=0.021<br>ATF2 ENCODE, P=0.019
NFS1	NFS1 cysteine desulfurase	NR2C2 ENCODE, P=0.018
ROMO1	reactive oxygen species modulator 1	NR2C2 ENCODE, P=0.018
RBM39	RNA binding motif protein 39	E2F1 CHEA, P=0.013<br>CREB1 CHEA, P=0.050<br>ATF2 ENCODE, P=0.019
PHF20	PHD finger protein 20	E2F1 CHEA, P=0.013<br>CREB1 CHEA, P=0.05<br>ATF2 ENCODE, P=0.019

* Data obtained by the Enrichr bioinformatic tools (https://maayanlab.cloud/Enrichr/), date of access 01.06.2020.
Databases indicated: GO (Gene Ontology), WP (WikiPathways 2019 Human), KEGG, Reactome, BioCarta, Panther, BP (BioPlanet 2019), KEGG, NCI-Nature, HumanCyc 2016, Virus-Host PPI P-HIPSTer 2020, PWY (BioCyc), Elsevier Pathway Collection (EPC), GWAS catalog, ENCODE, CHEA 2016.
