# Supplementary material for: The Link between Type 2 Diabetes Mellitus and the Polymorphisms of Glutathione-Metabolizing Genes Suggests a New Hypothesis Explaining Disease Initiation and Progression
Source: Life (Basel). 2021 Aug 28;11(9):886. doi: 10.3390/life11090886 (PMC8466482; doi:10.3390/life11090886)
Supplement: Supplementary file 1 [file life-11-00886-s001.zip › Suppl Data S11.pdf]

Supplementary data 11. Tissue-specific gene expression correlated with T2D-associated alleles of *GSS* and *GGT7* genes and disease pathogenesis

The figure depicts the puzzle showing genes whose tissue-specific changes in expression level were correlated with a carriage of the T2D-associated polymorphisms of *GSS* and *GGT7* genes, and associated with the risk of type 2 diabetes. Understanding a source of these complex interrelationships will bring us to deciphering the molecular mechanisms by which the *GSS* and *GGT7* genes and other genes for glutathione metabolism are involved in the pathophysiology of type 2 diabetes.

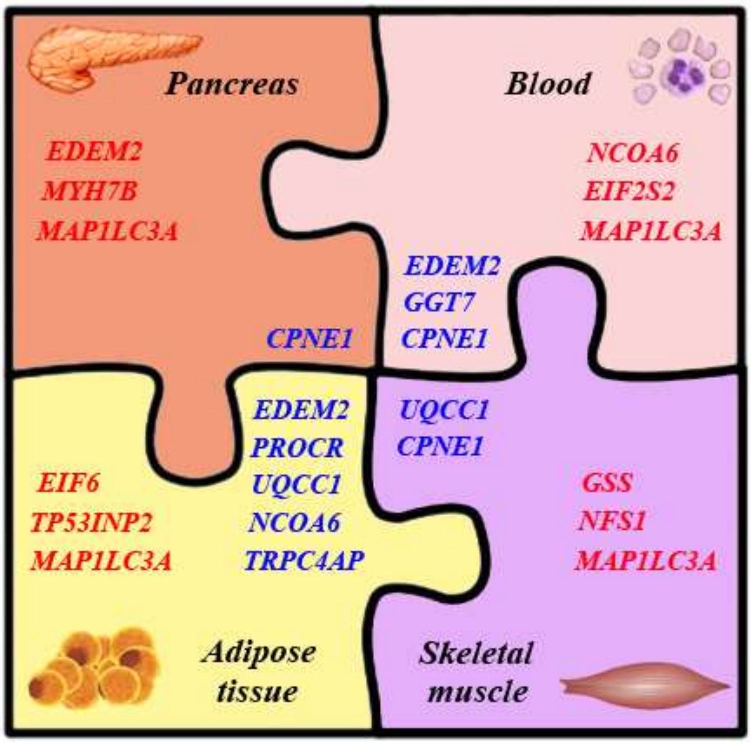

Figure. Tissue-specific changes in the expression level of genes that associated with the disease related polymorphisms of *GSS* and *GGT7* genes, as *in silico* predicted by the TWAS analysis in type diabetes

Blue and red colors depict genes whose tissue expression in diabetics is decreased and increased, respectively. EDEM2, endoplasmic reticulum degradation-enhancing alpha-mannosidase-like protein 2; MYH7B, myosin heavy chain 7B; PROCR, endothelial protein C receptor; MAP1LC3A, microtubule-associated proteins 1A/1B light chain 3A; TRPC4AP, short transient receptor potential channel 4-associated protein; TP53INP2, tumor protein p53-inducible nuclear protein 2; NFS1, mitochondrial cysteine desulfurase; EIF2S2, eukaryotic translation initiation factor 2 subunit beta; EIF6, eukaryotic translation initiation factor 6; CPNE1, cAMP responsive element binding protein 1; UQCC1, ubiquinol-cytochrome-c reductase complex assembly factor 1; GGT7, gamma-glutamyltransferase 7; GSS, glutathione synthetase.

Pursuing this interest, we carried out an in-depth analysis of the literature on the biological functions of genes whose expression is interrelated with polymorphic variants of the *GSS* and *GGT7* genes associated with the development of type 2 diabetes. A primary goal was to analyze biological functions of four genes such as *EDEM2*, *MYH7B*, *MAP1LC3A*, and *CPNE1* whose expression level in the pancreas was associated with a carriage of T2D-associated alleles of *GSS* and *GGT7*. Our interest in the pancreas is due to the fact that beta-cells located in this organ produce insulin, a hormone whose impaired maturation and secretion is linked to the development of type 2 diabetes mellitus.

EDEM2 is endoplasmic reticulum (ER) degradation-enhancing alpha-mannosidase-like protein 2, a protein involved in the ER-associated degradation (ERAD) pathway targeting misfolded glycoproteins for degradation in an N-glycan-dependent manner [64]. Endoplasmic reticulum is a multifunctional organelle that represents the Ca2 + reservoir in the cell, the site of protein assembly and folding, the site of steroid synthesis, as well as the platform of intercellular and interorganellar signaling [65]. To restore the accuracy of protein folding, the cell triggers unfolded protein response (UPR), a network of signaling pathways for reprogramming gene transcription, mRNA translation

and post-translational protein modification to restore protein homeostasis also called as proteostasis [65]. Endoplasmic reticulum stress (ER-stress) and associated UPR have been implicated in the pathogenesis of type 2 diabetes as a potential contributor to pancreatic beta-cell dysfunction and linked to diabetes-related phenotypes such as insulin resistance and obesity [66]. It is important to note the loss of activity of UPR participants such as IRE1α, XBP1, ATF6, PERK, and ATF4 is associated with both dysfunction and death of pancreatic beta-cells [67]. As a part of unfolded protein response induced by ER-stress, misfolded and unassembled proteins of the endoplasmic reticulum are subjected to recognition and elimination by the ERAD system, an evolutionarily conserved protein quality control system that destroys the targeted proteins by the cytoplasmic ubiquitin-proteasome pathway [68]. It is important to note that the ERAD system plays a critical role in supporting glucose-stimulated insulin secretion in pancreatic β-cells, and ERAD deficiency may be responsible for the development of diabetes through the impairment of glucose-stimulated insulin secretion that is due to affecting both folding-competent and folding-deficient processing of proteins in the ER [69]. Interestingly, loss-of-function mutations in the *EDEM2* gene are responsible for early-onset childhood diabetes [70]. Experimental down-regulation of *Edem2* in rat β-cells has been found to reduce the expression of genes involved in the glucose-stimulated insulin secretion pathway such as *Glut2* and *Pxd1*, ultimately leading to suppression of insulin secretion, but not apoptosis [70]. The TWAS analysis enabled us identifying that the expression level of *EDEM2* was increased in the pancreas in diabetics, pointing out that *EDEM2* is potentially involved in the ER-associated degradation of proteins in pancreatic tissue, in particular, it may target misfolded and unfolded proinsulin for proteasomal degradation. We hypothesize that the increased expression of *EDEM2* in the pancreas in patients with T2D may be considered as a sign of adaptive activation of the ER-associated protein degradation as a part of unfolded protein response, the hallmark in type 2 diabetes pathogenesis.

Pancreatic changes in the expression of *MYH7B*, a structural part of myosin in non-muscle cells, pays a special attention since the protein may be involved the transmembrane transport of glucose. MYH7B, myosin heavy chain 7B is a structural part of myosin, the major contractile protein mostly expressed in cardiomyocytes and skeletal muscle cells. It is well known that skeletal muscle is the major site for insulin-stimulated glucose disposal, and skeletal muscle insulin resistance is the key pathological condition in type 2 diabetes [71]. Alterations in the expression of myosin heavy chains in skeletal muscle can be induced by a variety of factors including physical activity, obesity, aging, and high fat/high sugar diet, i.e. the factors playing an essential role in the development of type 2 diabetes [72]. It has been revealed that myosin heavy chain isoforms impact insulin-stimulated glucose uptake by skeletal muscle cells in rats [73]. Interestingly, MYH7B is also found in non-muscle cells, but nothing is known about its functional roles in such types of cells including beta-cells of the pancreatic islets. A study in humans showed that the non-muscle myosin *MYH9* (myosin heavy chain 9, a marker of fast-twitch oxidative fiber and an analog of MYH7B) gene expression related positively with insulin-stimulated glucose uptake, whereas the expression levels of muscle myosin *MYH1* (myosin heavy chain 1, a marker fast-twitch glycolytic fiber) related negatively with insulin-stimulated glucose uptake [74]. Other studies have established that non-muscle myosins may play a role in the GLUT4 (insulin-responsive glucose transporter type 4) vesicle trafficking required for insulin-stimulated glucose uptake [75]. Interestingly, Liu with co-authors have recently observed an increased expression of *Myh7b* among 10 differentially expressed genes in the liver of rats with experimental type 2 diabetes [20]. A 5-week therapy of T2D rats with walnut meal extracts (walnut is rich in polyphenols and glutathione) resulted in decreasing of *Myh7b* expression that accompanied by a) decreasing fasting blood glucose and insulin resistance, b) increasing β-cell function and insulin sensitivity index, c) improving body weight, oral glucose tolerance test and insulin sensitivity, d) increasing the activities of antioxidant enzymes, and e) decreasing the levels of malondialdehyde in the liver and serum [76]. These experimental findings clearly show that the restoration of glutathione content in diabetics has the potential of decreasing fasting blood glucose, improving insulin sensitivity, and reducing oxidative stress. The TWAS analysis allowed us to observe the association between the increased levels of *MYH7B* in pancreatic tissue and with type 2 diabetes, supporting the findings of Li with co-authors [186] that there is a causal relationship between the increased level of *MYH7B* and susceptibility to type 2 disease. It can be assumed that the increased tissue level of *MYH7B* in diabetics may indicate increased insulin-stimulated glucose uptake by the cell that may be due to intracellular deficiency of glutathione. Although it is obvious that this protein is important for the disease development, at least due to its participation in the regulation of insulin-stimulated glucose uptake by the cell, a limited information on functions of this form of myosin in non-muscle cells does not now allow to draw a definitive conclusion on the roles of *MYH7B* in the pathogenesis of type 2 diabetes.

Autophagy is a post-endoplasmic reticulum pathway necessary for effective turnover of misfolded or aggregated proteins, clearing damaged organelles as well as for cellular survival under various stress stimuli [77]. In the pancreatic b-cells, autophagy is important for regulating proinsulin levels and insulin secretion, and the autophagic

degradation of proinsulin may have pathophysiological implications for the development of insulin deficiency in type 2 diabetes [78]. MAP1LC3A, microtubule-associated proteins 1A/1B light chain 3A is ubiquitin-like modifier protein mediating the physical interactions between microtubules and components of the cytoskeleton and involved in the formation of autophagosomes [79]. Upon nutrient stress, MAP1LC3A together with the reticulophagy receptor TEX264 plays a role in the remodeling of subdomains of the endoplasmic reticulum into autophagosomes [80], thereby playing a role in the regulation of autophagy. Autophagy is a post-endoplasmic reticulum pathway necessary for effective turnover of misfolded or aggregated proteins, clearing damaged organelles as well as for cellular survival under various stress stimuli [81]. In the pancreatic b-cells, autophagy is important for regulating proinsulin levels and insulin secretion, and the autophagic degradation of proinsulin may have pathophysiological implications for the development of insulin deficiency in type 2 diabetes [82]. Urinary expression of MAP1LC3A is considered as autophagy markers in patients with diabetic kidney disease [83]. Notably, the TWAS analysis allowed us identifying that the increased expression of *MAP1LC3A* in the pancreas, skeletal muscle, adipose tissue, and whole blood is associated with the susceptibility to type 2 diabetes. An increased multi-tissue expression level of *MAP1LC3A*, an ubiquitin-like modifier protein involved in the formation of autophagosomes [89], may indicate the systemic activation of autophagy, a post-endoplasmic reticulum pathway focused on effective turnover of misfolded or aggregated proteins [82]. In the pancreatic beta-cells, increased MAP1LC3A may have a protective role against ER-stress-mediated cell death through the autophagic degradation of misfolded or unfolded proinsulin molecules that have been established in type 2 diabetes [84,85,86]. It can be assumed that an increase in *MAP1LC3A* expression in the pancreas, skeletal muscle, and adipose tissue in diabetics depicts the increased autophagosome activation as a process of physiologic response of pancreas and insulin-sensitive tissues to the ER-induced stress and unfolded protein response attributed to the accumulation of unfolded/misfolded proteins in the ER lumen. A crosstalk between unfolded protein response and autophagy is known form the literature: the UPR may induce autophagy and vice versa autophagy can alleviate the UPR.

CPNE1, cAMP responsive element binding protein 1, is a calcium-dependent phospholipid-binding protein regulating a variety of intracellular molecular processes at the interface of the cell membrane and cytoplasm such as membrane trafficking, recruiting target proteins to the cell membrane [87,88,89]. CPNE1 is involved in TNF-alpha-induced transcriptional repression of NF-kappa-B by inducing endoprotease processing of transcription factor-protein complexes including NF-kappa-B p65/NFKB1, NF-kappa-B p50/NFKB1, p52/NFKB2, RELB and REL [90,91]. Interestingly, the increased levels of *Cpne1* were found in the β-cells of the pancreas of the streptozotocin-diabetic rats, a model of the chronic complications of human type 2 diabetes [202]. Since CPNE1 has the potential to control the transcription of NF-kappa-B, involved in cellular responses to various stress stimuli such as cytokines, free radicals, heavy metals, etc, and transcriptional repression of NF-kappa-B by CPNE1 could impact the inflammatory response and cell survival through susceptibility of the cell to apoptosis [93], a key pathophysiological process underlying β-cell damage in type 2 diabetes [94]. Notably, the TWAS analysis has shown that decreased levels of *CPNE1* in the pancreas, skeletal muscle, and whole blood were associated with the risk of type 2 diabetes. Based on existing biological functions of CPNE1, its decreased level, on the one hand, may be indicative for the inflammatory state of these tissues and their increased susceptibility to apoptosis, on the other hand, the decreased *CPNE1* may impair insulin secretion through the cell membrane. In addition, since CPNE1 exhibits a repressive effect on transcription factor NF-kappa-B, the decreased levels of CPNE1 in diabetics may facilitate an excessive activation of NF-κB signaling leading to induction of pro-inflammatory genes, including cytokines and chemokines that play a role in the pathogenesis of type 2 diabetes [95].

As can be seen from Figure, several other genes whose expression levels in insulin-sensitive tissues such as skeletal muscle, adipose tissue and also in whole blood were found to be correlated with both the genetic risk of type 2 diabetes and a carriage of disease-associated alleles of *GSS* and *GGT7* genes.

The *TP53INP2* (tumor protein p53-inducible nuclear protein 2) gene encodes a bifunctional protein acting as a transcriptional coactivator and as a key regulator of basal autophagy by stimulation of autophagosome formation and ubiquitinated protein degradation [96,97]. We found that *TP53INP2* expression in whole blood is negatively correlated with a carriage of T2D-associated allele rs11546155-A of the *GGT7* gene, thereby suggesting the link between glutathione metabolism with both autophagy and protein degradation.

The decreased expression of TRPC4AP in adipose tissue and liver were correlated with a carriage of T2D-associated allele rs13041792-A of the *GSS* gene. TRPC4AP (short transient receptor potential channel 4-associated protein) is a membrane protein involved in the transport of extracellular Ca2+ to cytosol and elevation of endoplasmic reticulum Ca^{2+} storage reduction in response to muscarinic acetylcholine receptor-1 [98]. TRPC4AP was enriched with the PathCards pathway "Transport of glucose and other sugars, bile salts and organic acids, metal ions and amine

compounds" (https://www.genecards.org) suggesting that the protein may participate in the transport of glucose across the cell membrane. The involvement of TRPC4AP in glucose transport can be supported by the findings of Malenczyk with coworkers who established the increased expression of *TRPC4AP* in β-cells from patients with T2D diabetics [99]. TRPC4AP acts as a substrate-specific adapter for a DCX E3 ubiquitin-protein ligase complex which participates in the control of cell cycle and specifically mediates the polyubiquitination and subsequent degradation of MYC [98], a transcription factor that is known to upregulate glucose and glutamine transporters as well as key enzymes in the glycolytic or glutaminolysis pathways [100]. In addition, TRPC4AP plays an essential role in regulating inflammation via the meditation of NF-κB signaling in inflammation [98,101], and *trpc4ap*-knockout mice exhibit decreased serum glucose and insulin levels along with decreased expression of pro-inflammatory cytokines which accompanied by the inactivation of the IKKα/IκBα/NF-κB pathway [101,102].

The TWAS bioinformatics analysis allowed us identifying the decreased expression of *NCOA6* in adipose tissue and increased expression of *NCOA6* in blood was associated with the genetic risk of type 2 diabetes. NCOA6 is a nuclear receptor coactivator 6 with a range of pleiotropic effects on various biological functions including growth, development, wound healing, and maintenance of energy homeostasis [103]. Experimental studies provided some evidence that NCOA6 could be a biomarker of human type 2 diabetes, and the involvement of NCOA6 in T2D pathogenesis might be related to the regulation of insulin secretion in the cellular response to a glucose stimulus [104]. A study conducted by Yeom and colleagues [105] showed that the overexpression of *Ncoa6* increased glucose-elicited insulin secretion, whereas *Ncoa6*-knockout mice exhibited the decreased insulin secretion in pancreatic islets. Moreover, *Ncoa6*-knockout mice showed reduced islet mass and number, which correlated with increased apoptosis and decreased proliferation of their pancreatic islets. Taking together, these findings point out that NCOA6 is an important regulator of insulin secretion and β-cell mass maintenance, and the reduced expression or activity of NCOA6 may linked with abnormal insulin secretion, a characteristic feature of type 2 diabetes. Additionally, NCOA6 is a co-activator of NF-kappa-B pathway [106], therefore the increased blood levels of *NCOA6* may be responsible for stimulating cellular signaling pathways linking to inflammation that play a role in type 2 diabetes.

We found that the increased levels of *NFS1* in skeletal muscle are related to genetic susceptibility to type 2 diabetes and was found to be positively correlated to a carriage of T2D-associated allele (rs13041792-A) at glutathione synthetase gene. NFS1 (cysteine desulfurase) is a mitochondrial enzyme that belongs to the family sulfurtransferases involved in the transfer of sulfur-containing groups. As a part of the sulfur relay system, NFS1 supplies inorganic sulfur to the Fe-S clusters required for the function of cellular enzymes by removing the sulfur from cysteine and other sulfur-containing amino acids [107,108] as well as known to play essential roles in energy metabolism, transcriptional regulation, DNA repair, and biosynthesis of nucleotides and amino acids [109]. Importantly, the maturation of Fe-S proteins requires glutathione [110]. Interestingly, the expression levels of NFS1 are significantly repressed in skeletal muscle from *Ndufab1*-knockout mice and accompanied by disruption of glucose homeostasis [111]. NDUFAB1 is an NADH: ubiquinone oxidoreductase subunit AB1, also known as mitochondrial acyl carrier protein transferring electrons from NADH to the respiratory chain [112] and acting as an enhancer of mitochondrial metabolism, protecting against obesity and insulin resistance [111]. Thus, the increased expression of *NFS1* may reflect, on the one hand, activation of the sulfur relay system, on the other hand, enhanced mitochondrial metabolism. It is known that mitochondrial function represents an integral part of glucose-stimulated insulin secretion in pancreatic β-cells and plays a key role in skeletal muscle oxidative phosphorylation [113]. It is well known that a pronounced increase in mitochondrial respiration is required for the synthesis and release of insulin by pancreatic β-cells adequately to uptake and metabolism of nutrients. Mitochondrial dysfunction as an important part of the pathogenesis of insulin resistance and T2D is manifested by high production of reactive oxygen species and low levels of ATP and thought to be related to changes in the autophagic process, endoplasmic reticulum stress, and inflammation [114]. Thus, based on literature data of biological functions of NFS1, we suggest that the increased expression of *NFS1* in insulin-resistant skeletal muscle cells occurring in diabetics, on the one hand, may be considered as a marker of adaptive enhance of mitochondrial functions for efficient energy production in the skeletal muscle requires oxidative phosphorylation [115, 116], on the other hand, the increased expression of *NFS1* in skeletal muscle in diabetics may mirror a demand of mitochondria in the active Fe-S proteins required for energy metabolism [109], and the lack of active Fe-S proteins is known to be exacerbated by a deficiency of intracellular glutathione [110].

The TWAS analysis showed that decreased levels of *UQCC1* in insulin-sensitive tissues such as skeletal muscle and adipose tissues are strongly associated with a diagnosis of type 2 diabetes in two cohorts from the UK Biobank (Figure 3). Moreover, the present study showed that two polymorphisms such as rs13041792 of *GSS* and rs6119534 of *GGT7* are associated with both the risk of type 2 diabetes and decreased levels of *UQCC1* in whole blood. This finding may suggest a relationship between glutathione metabolism and mitochondrial enzymes participating in cell

respiration and ATP production. UQCC1, ubiquinol-cytochrome-c reductase complex assembly factor 1 is a protein required for the assembly of the ubiquinol-cytochrome c reductase complex (complex III) is an energy-transducing, electron-transfer enzyme located in the inner mitochondrial membrane and participated in cell respiration [117]. This mitochondrial complex oxidizes quinol and reduces a water-soluble, c-type cytochrome linking this redox reaction to translocation of protons across the membrane, thereby contributing to the generation of proton motive force used for ATP synthesis [118]. UQCC1 promotes complex III synthesis and stability, thereby participating in cytochrome *b* biogenesis [117,119]. Taking into account the above functions of UQCC1, the decreased level of *UQCC1* in adipose tissue and skeletal muscle might be responsible for UQCC1-dependent perturbation in cytochrome *b* expression and subsequent decreasing the efficiency of cellular energy conversion, increasing ROS production, ultimately leading to oxidative stress, mitochondrial dysfunction - characteristic metabolic abnormalities underlying type 2 diabetes [120]. We also found that the increased levels of *EIF6* in adipose tissue were correlated to the risk of type 2 diabetes. EIF6 is eukaryotic translation initiation factor 6 also known as integrin beta 4 binding protein. EIF6 acts as a stimulatory factor for the initiation of translation of downstream insulin or growth factors, and cells with the decreased amount of EIF6 do not increase translation in response to insulin [121,122]. Moreover, EIF6 has been shown to control fatty acid synthesis and glycolysis in liver and fat tissue, where EIF6 acts by exerting translational control of adipogenic transcription factors such as CEBPB, CEBPD, and ATF4, thereby reshaping gene expression with increased levels of lipogenic and glycolytic enzymes [123]. It is important to add that EIF6 controls the transcriptional activity of genes encoding mitochondrial respiratory chain producing reactive oxygen species [124], and it has been revealed a correlation between expression of EIF6 and the levels of C-reactive protein, a marker of systemic inflammation [125]. We propose that the increased levels of *EIF6* in adipose tissue of patients with type 2 diabetes appear to enhance the production of adipogenic transcription factors activating the expression of genes for lipogenic and glycolytic enzymes necessary for energy production. The TWAS bioinformatics analysis revealed that a sharp increase in blood levels of *EIF2S2* is genetically linked to type 2 diabetes (Figure 3) and also associated with a carriage of rs13041792-A of GSS that has been also associated with the disease risk. EIF2S2 (eukaryotic translation initiation factor 2 subunit beta) is a member of eukaryotic translation initiation factors (eIF2) that functions in the early steps of protein synthesis through the formation of the ternary complex with GTP and initiator tRNA and binding to the 40S subunit of ribosomes [126]. Like eIF2, phosphorylation-dephosphorylation reactions of EIF2S2 contribute to glucose-induced activation of protein biosynthesis [127]. The eIF2α kinase PERK (RNA-dependent protein kinase-like ER kinase) is the key mediator of the translation arm of the unfolded protein response by rapidly decreasing the protein load on the endoplasmic reticulum via translational repression of mRNAs encoding secreted proteins [128]. Palam with colleagues suggested that high eIF2 levels are required for efficient translation of the target genes during ER stress-induced unfolded protein response [129]. Thus, based on biological functions of EIF2S2, the increased expression of this gene in blood of diabetes may be interpreted as a sign of the repressed protein synthesis machinery attributed to the endoplasmic reticulum ER stress and activation of unfolded protein response. The TWAS analysis has shown that increased level of *EIF2S2* along with decreased level of *GGT7* in blood are linked with genetic risk of type 2 diabetes. We suggest that the decreased level of *GGT7* in blood in diabetics mirrors a deficiency of this enzyme that may cause a decreased rate of glutathione utilization by peripheral tissues since gamma-glutamyl transferase is involved in glutathione recycling, supplementing the cell with GSH precursors. Thus, tissue-specific changes in gene expression that correlated with T2D-associated alleles of *GSS* and *GGT7* genes may mirror various pathological disorders established in type 2 diabetes mellitus.

Supplementary references

64. Ninagawa S, Okada T, Sumitomo Y, Kamiya Y, Kato K, Horimoto S, Ishikawa T, Takeda S, Sakuma T, Yamamoto T, Mori K. EDEM2 initiates mammalian glycoprotein ERAD by catalyzing the first mannose trimming step. J Cell Biol. 2014 Aug 4;206(3):347-56. doi: 10.1083/jcb.201404075.

65. Hetz C, Zhang K, Kaufman RJ. Mechanisms, regulation and functions of the unfolded protein response. Nat Rev Mol Cell Biol. 2020 Aug;21(8):421-438. doi: 10.1038/s41580-020-0250-z.

66. Ozcan U, Yilmaz E, Ozcan L, Furuhashi M, Vaillancourt E, Smith RO, Görgün CZ, Hotamisligil GS. Chemical chaperones reduce ER stress and restore glucose homeostasis in a mouse model of type 2 diabetes. Science. 2006 Aug 25;313(5790):1137-40. doi: 10.1126/science.1128294.

67. Charbord J, Ren L, Sharma RB, Johansson A, Ågren R, Chu L, Tworus D, Schulz N, Charbord P, Stewart AF, Wang P, Alonso LC, Andersson O. In vivo screen identifies a SIK inhibitor that induces β cell proliferation through a transient UPR. Nat Metab. 2021 May;3(5):682-700. doi: 10.1038/s42255-021-00391-x.

68. Meusser B, Hirsch C, Jarosch E, Sommer T. ERAD: the long road to destruction. Nat Cell Biol. 2005 Aug;7(8):766-72. doi: 10.1038/ncb0805-766.
69. Hu Y, Gao Y, Zhang M, Deng KY, Singh R, Tian Q, Gong Y, Pan Z, Liu Q, Boisclair YR, Long Q. Endoplasmic Reticulum-Associated Degradation (ERAD) Has a Critical Role in Supporting Glucose-Stimulated Insulin Secretion in Pancreatic β-Cells. Diabetes. 2019 Apr;68(4):733-746. doi: 10.2337/db18-0624.
70. Alhaidan Y, Christesen HT, Højlund K, Al Balwi MA, Brusgaard K. A novel gene in early childhood diabetes: EDEM2 silencing decreases SLC2A2 and PXD1 expression, leading to impaired insulin secretion. Mol Genet Genomics. 2020 Sep;295(5):1253-1262. doi: 10.1007/s00438-020-01695-5.
71. DeFronzo RA, Tripathy D. Skeletal muscle insulin resistance is the primary defect in type 2 diabetes. Diabetes Care. 2009 Nov;32 Suppl 2(Suppl 2):S157-63. doi: 10.2337/dc09-S302.
72. Hyatt JP, Nguyen L, Hall AE, Huber AM, Kocan JC, Mattison JA, de Cabo R, LaRocque JR, Talmadge RJ. Muscle-Specific Myosin Heavy Chain Shifts in Response to a Long-Term High Fat/High Sugar Diet and Resveratrol Treatment in Nonhuman Primates. Front Physiol. 2016 Mar 2;7:77. doi: 10.3389/fphys.2016.00077.
73. Mackrell JG, Cartee GD. A novel method to measure glucose uptake and myosin heavy chain isoform expression of single fibers from rat skeletal muscle. Diabetes. 2012 May;61(5):995-1003. doi: 10.2337/db11-1299.
74. Olsson AH, Rönn T, Elgzyri T, Hansson O, Eriksson KF, Groop L, Vaag A, Poulsen P, Ling C. The expression of myosin heavy chain (MHC) genes in human skeletal muscle is related to metabolic characteristics involved in the pathogenesis of type 2 diabetes. Mol Genet Metab. 2011 Jul;103(3):275-81. doi: 10.1016/j.ymgme.2011.03.017.
75. Woody S, Stall R, Ramos J, Patel YM. Regulation of myosin light chain kinase during insulin-stimulated glucose uptake in 3T3-L1 adipocytes. PLoS One. 2013 Oct 8;8(10):e77248. doi: 10.1371/journal.pone.0077248.
76. Li Y, Chen D, Xu C, Zhao Q, Ma Y, Zhao S, Chen C. Glycolipid metabolism and liver transcriptomic analysis of the therapeutic effects of pressed degreased walnut meal extracts on type 2 diabetes mellitus rats. Food Funct. 2020 Jun 24;11(6):5538-5552. doi: 10.1039/d0fo00670j.
77. Glick D, Barth S, Macleod KF. Autophagy: cellular and molecular mechanisms. J Pathol. 2010 May;221(1):3-12. doi: 10.1002/path.2697.
78. Riahi Y, Wikstrom JD, Bachar-Wikstrom E, Polin N, Zucker H, Lee MS, Quan W, Haataja L, Liu M, Arvan P, Cerasi E, Leibowitz G. Autophagy is a major regulator of beta cell insulin homeostasis. Diabetologia. 2016 Jul;59(7):1480-1491. doi: 10.1007/s00125-016-3868-9.
79. Cherra SJ 3rd, Kulich SM, Uechi G, Balasubramani M, Mountzouris J, Day BW, Chu CT. Regulation of the autophagy protein LC3 by phosphorylation. J Cell Biol. 2010 Aug 23;190(4):533-9. doi: 10.1083/jcb.201002108.
80. An H, Ordureau A, Paulo JA, Shoemaker CJ, Denic V, Harper JW. TEX264 Is an Endoplasmic Reticulum-Resident ATG8-Interacting Protein Critical for ER Remodeling during Nutrient Stress. Mol Cell. 2019 Jun 6;74(5):891-908.e10. doi: 10.1016/j.molcel.2019.03.034.
81. Glick D, Barth S, Macleod KF. Autophagy: cellular and molecular mechanisms. J Pathol. 2010 May;221(1):3-12. doi: 10.1002/path.2697.
82. Riahi Y, Wikstrom JD, Bachar-Wikstrom E, Polin N, Zucker H, Lee MS, Quan W, Haataja L, Liu M, Arvan P, Cerasi E, Leibowitz G. Autophagy is a major regulator of beta cell insulin homeostasis. Diabetologia. 2016 Jul;59(7):1480-1491. doi: 10.1007/s00125-016-3868-9.
83. Matboli M, Azazy AEM, Adel S, Bekhet MM, Eissa S. Evaluation of urinary autophagy transcripts expression in diabetic kidney disease. J Diabetes Complications. 2017 Oct;31(10):1491-1498. doi: 10.1016/j.jdiacomp.2017.06.009.
84. Sun J, Cui J, He Q, Chen Z, Arvan P, Liu M. Proinsulin misfolding and endoplasmic reticulum stress during the development and progression of diabetes. Mol Aspects Med. 2015 Apr;42:105-18. doi: 10.1016/j.mam.2015.01.001.
85. Haataja L, Manickam N, Soliman A, Tsai B, Liu M, Arvan P. Disulfide Mispairing During Proinsulin Folding in the Endoplasmic Reticulum. Diabetes. 2016 Apr;65(4):1050-60. doi: 10.2337/db15-1345.
86. Arunagiri A, Haataja L, Pottekat A, Pamenan F, Kim S, Zeltser LM, Paton AW, Paton JC, Tsai B, Itkin-Ansari P, Kaufman RJ, Liu M, Arvan P. Proinsulin misfolding is an early event in the progression to type 2 diabetes. Elife. 2019 Jun 11;8:e44532. doi: 10.7554/eLife.44532

87. Tomsig JL, Snyder SL, Creutz CE. Identification of targets for calcium signaling through the copine family of proteins. Characterization of a coiled-coil copine-binding motif. J Biol Chem. 2003 Mar 21;278(12):10048-54. doi: 10.1074/jbc.M212632200.

88. Tomsig JL, Sohma H, Creutz CE. Calcium-dependent regulation of tumour necrosis factor-alpha receptor signalling by copine. Biochem J. 2004 Mar 15;378(Pt 3):1089-94. doi: 10.1042/BJ20031654.

89. Creutz CE, Tomsig JL, Snyder SL, Gautier MC, Skouri F, Beisson J, Cohen J. The copines, a novel class of C2 domain-containing, calcium-dependent, phospholipid-binding proteins conserved from Paramecium to humans. J Biol Chem. 1998 Jan 16;273(3):1393-402. doi: 10.1074/jbc.273.3.1393.

90. Ramsey CS, Yeung F, Stoddard PB, Li D, Creutz CE, Mayo MW. Copine-I represses NF-kappaB transcription by endoproteolysis of p65. Oncogene. 2008 Jun 5;27(25):3516-26. doi: 10.1038/sj.onc.1211030.

91. Lu ZG, Liu H, Yamaguchi T, Miki Y, Yoshida K. Protein kinase Cdelta activates RelA/p65 and nuclear factor-kappaB signaling in response to tumor necrosis factor-alpha. Cancer Res. 2009 Jul 15;69(14):5927-35. doi: 10.1158/0008-5472.CAN-08-4786.

92. Altirriba J, Barbera A, Del Zotto H, Nadal B, Piquer S, Sánchez-Pla A, Gagliardino JJ, Gomis R. Molecular mechanisms of tungstate-induced pancreatic plasticity: a transcriptomics approach. BMC Genomics. 2009 Aug 28;10:406. doi: 10.1186/1471-2164-10-406.

93. Sheikh MS, Huang Y. Death receptor activation complexes: it takes two to activate TNF receptor 1. Cell Cycle. 2003 Nov-Dec;2(6):550-2.

94. Butler AE, Janson J, Bonner-Weir S, Ritzel R, Rizza RA, Butler PC. Beta-cell deficit and increased beta-cell apoptosis in humans with type 2 diabetes. Diabetes. 2003 Jan;52(1):102-10. doi: 10.2337/diabetes.52.1.102.

95. Baker RG, Hayden MS, Ghosh S. NF-κB, inflammation, and metabolic disease. Cell Metab. 2011 Jan 5;13(1):11-22. doi: 10.1016/j.cmet.2010.12.008.

96. Francis VA, Zorzano A, Teleman AA. dDOR is an EcR coactivator that forms a feed-forward loop connecting insulin and ecdysone signaling. Curr Biol. 2010 Oct 26;20(20):1799-808. doi: 10.1016/j.cub.2010.08.055.

97. Sala D, Ivanova S, Plana N, Ribas V, Duran J, Bach D, Turkseven S, Laville M, Vidal H, Karczewska-Kupczewska M, Kowalska I, Straczkowski M, Testar X, Palacín M, Sandri M, Serrano AL, Zorzano A. Autophagy-regulating TP53INP2 mediates muscle wasting and is repressed in diabetes. J Clin Invest. 2014 May;124(5):1914-27. doi: 10.1172/JCI72327.

98. Choi SH, Wright JB, Gerber SA, Cole MD. Myc protein is stabilized by suppression of a novel E3 ligase complex in cancer cells. Genes Dev. 2010 Jun 15;24(12):1236-41. doi: 10.1101/gad.1920310.

99. Malenczyk K, Girach F, Szodorai E, Storm P, Segerstolpe Å, Tortoriello G, Schnell R, Mulder J, Romanov RA, Borók E, Piscitelli F, Di Marzo V, Szabó G, Sandberg R, Kubicek S, Lubec G, Hökfelt T, Wagner L, Groop L, Harkany T. A TRPV1-to-secretagogin regulatory axis controls pancreatic β-cell survival by modulating protein turnover. EMBO J. 2017 Jul 14;36(14):2107-2125. doi: 10.15252/embj.201695347.

100. Peterson CW, Ayer DE. An extended Myc network contributes to glucose homeostasis in cancer and diabetes. Front Biosci (Landmark Ed). 2011 Jun 1;16:2206-23. doi: 10.2741/3848.

101. Zhu Q, Zhu YY, Wang WN. TRUSS inhibition protects against high fat diet (HFD)-stimulated brain injury by alleviation of inflammatory response. Biochem Biophys Res Commun. 2019 Mar 26;511(1):41-48. doi: 10.1016/j.bbrc.2019.01.058.

102. Yu CJ, Wang QS, Wu MM, Song BL, Liang C, Lou J, Tang LL, Yu XD, Niu N, Yang X, Zhang BL, Qu Y, Liu Y, Dong ZC, Zhang ZR. TRUSS Exacerbates NAFLD Development by Promoting IκBα Degradation in Mice. Hepatology. 2018 Nov;68(5):1769-1785. doi: 10.1002/hep.30066.

103. Lu SC. Glutathione synthesis. Biochim Biophys Acta. 2013 May;1830(5):3143-53. doi: 10.1016/j.bbagen.2012.09.008.

104. Kim GH, Lee KJ, Oh GS, Yoon J, Kim HW, Kim SW. Regulation of hepatic insulin sensitivity by activating signal co-integrator-2. Biochem J. 2012 Nov 1;447(3):437-47. doi: 10.1042/BJ20120861.

105. Yeom SY, Kim GH, Kim CH, Jung HD, Kim SY, Park JY, Pak YK, Rhee DK, Kuang SQ, Xu J, Han DJ, Song DK, Lee JW, Lee KU, Kim SW. Regulation of insulin secretion and beta-cell mass by activating signal cointegrator 2. Mol Cell Biol. 2006 Jun;26(12):4553-63. doi: 10.1128/MCB.01412-05.

106. Wu L, Zhao KQ, Wang W, Cui LN, Hu LL, Jiang XX, Shuai J, Sun YP. Nuclear receptor coactivator 6 promotes HTR-8/SVneo cell invasion and migration by activating NF-κB-mediated MMP9 transcription. Cell Prolif. 2020 Sep;53(9):e12876. doi: 10.1111/cpr.12876.

107. Marelja Z, Stöcklein W, Nimtz M, Leimkühler S. A novel role for human Nfs1 in the cytoplasm: Nfs1 acts as a sulfur donor for MOCS3, a protein involved in molybdenum cofactor biosynthesis. J Biol Chem. 2008 Sep 12;283(37):25178-25185. doi: 10.1074/jbc.M804064200.

108. Pain D, Dancis A. Roles of Fe-S proteins: from cofactor synthesis to iron homeostasis to protein synthesis. Curr Opin Genet Dev. 2016 Jun;38:45-51. doi: 10.1016/j.gde.2016.03.006.

109. Beinert H. Iron-sulfur proteins: ancient structures, still full of surprises. J Biol Inorg Chem. 2000 Feb;5(1):2-15. doi: 10.1007/s007750050002.

110. Sipos K, Lange H, Fekete Z, Ullmann P, Lill R, Kispal G. Maturation of cytosolic iron-sulfur proteins requires glutathione. J Biol Chem. 2002 Jul 26;277(30):26944-9. doi: 10.1074/jbc.M200677200.

111. Zhang R, Hou T, Cheng H, Wang X. NDUFAB1 protects against obesity and insulin resistance by enhancing mitochondrial metabolism. FASEB J. 2019 Dec;33(12):13310-13322. doi: 10.1096/fj.201901117RR.

112. Stroud DA, Surgenor EE, Formosa LE, Reljic B, Frazier AE, Dibley MG, Osellame LD, Stait T, Beilharz TH, Thorburn DR, Salim A, Ryan MT. Accessory subunits are integral for assembly and function of human mitochondrial complex I. Nature. 2016 Oct 6;538(7623):123-126. doi: 10.1038/nature19754.

113. Kwak SH, Park KS, Lee KU, Lee HK. Mitochondrial metabolism and diabetes. J Diabetes Investig. 2010 Oct 19;1(5):161-9. doi: 10.1111/j.2040-1124.2010.00047.x.

114. Rocha M, Apostolova N, Diaz-Rua R, Muntane J, Victor VM. Mitochondria and T2D: Role of Autophagy, ER Stress, and Inflammasome. Trends Endocrinol Metab. 2020 Oct;31(10):725-741. doi: 10.1016/j.tem.2020.03.004.

115. Kelley DE, He J, Menshikova EV, Ritov VB. Dysfunction of mitochondria in human skeletal muscle in type 2 diabetes. Diabetes. 2002 Oct;51(10):2944-50. doi: 10.2337/diabetes.51.10.2944.

116. Patti ME, Corvera S. The role of mitochondria in the pathogenesis of type 2 diabetes. Endocr Rev. 2010 Jun;31(3):364-95. doi: 10.1210/er.2009-0027.

117. Tucker EJ, Wanschers BF, Szklarczyk R, Mountford HS, Wijeyeratne XW, van den Brand MA, Leenders AM, Rodenburg RJ, Reljić B, Compton AG, Frazier AE, Bruno DL, Christodoulou J, Endo H, Ryan MT, Nijtmans LG, Huynen MA, Thorburn DR. Mutations in the UQCC1-interacting protein, UQCC2, cause human complex III deficiency associated with perturbed cytochrome b protein expression. PLoS Genet. 2013;9(12):e1004034. doi: 10.1371/journal.pgen.1004034.

118. Sarewicz M, Osyczka A. Cytochrome bc1 Complex (Respiratory Chain Complex III), Reference Module in Life Sciences, Elsevier, 2020, https://doi.org/10.1016/B978-0-12-819460-7.00018-9.

119. Fernández-Vizarra E, Zeviani M. Nuclear gene mutations as the cause of mitochondrial complex III deficiency. Front Genet. 2015 Apr 9;6:134. doi: 10.3389/fgene.2015.00134.

120. Lee DW, Selamoglu N, Lanciano P, Cooley JW, Forquer I, Kramer DM, Daldal F. Loss of a conserved tyrosine residue of cytochrome b induces reactive oxygen species production by cytochrome bc1. J Biol Chem. 2011 May 20;286(20):18139-48. doi: 10.1074/jbc.M110.214460.

121. Brina D, Miluzio A, Ricciardi S, Clarke K, Davidsen PK, Viero G, Tebaldi T, Offenhäuser N, Rozman J, Rathkolb B, Neschen S, Klingenspor M, Wolf E, Gailus-Durner V, Fuchs H, Hrabe de Angelis M, Quattrone A, Falciani F, Biffo S. eIF6 coordinates insulin sensitivity and lipid metabolism by coupling translation to transcription. Nat Commun. 2015 Sep 18;6:8261. doi: 10.1038/ncomms9261.

122. Brina D, Grosso S, Miluzio A, Biffo S. Translational control by 80S formation and 60S availability: the central role of eIF6, a rate limiting factor in cell cycle progression and tumorigenesis. Cell Cycle. 2011 Oct 15;10(20):3441-6. doi: 10.4161/cc.10.20.17796.

123. Biffo S, Manfrini N, Ricciardi S. Crosstalks between translation and metabolism in cancer. Curr Opin Genet Dev. 2018 Feb;48:75-81. doi: 10.1016/j.gde.2017.10.011.

124. Ricciardi S, Miluzio A, Brina D, Clarke K, Bonomo M, Aiolfi R, Guidotti LG, Falciani F, Biffo S. Eukaryotic translation initiation factor 6 is a novel regulator of reactive oxygen species-dependent megakaryocyte maturation. J Thromb Haemost. 2015 Nov;13(11):2108-18. doi: 10.1111/jth.13150.

125. Clarke K, Ricciardi S, Pearson T, Bharudin I, Davidsen PK, Bonomo M, Brina D, Scagliola A, Simpson DM, Beynon RJ, Khanim F, Ankers J, Sarzynski MA, Ghosh S, Pisconti A, Rozman J, Hrabe de Angelis M, Bunce C, Stewart

C, Egginton S, Caddick M, Jackson M, Bouchard C, Biffo S, Falciani F. The Role of Eif6 in Skeletal Muscle Homeostasis Revealed by Endurance Training Co-expression Networks. Cell Rep. 2017 Nov 7;21(6):1507-1520. doi: 10.1016/j.celrep.2017.10.040.

126. Kimball SR. Eukaryotic initiation factor eIF2. Int J Biochem Cell Biol. 1999 Jan;31(1):25-9. doi: 10.1016/s1357-2725(98)00128-9.

127. Vander Mierde D, Scheuner D, Quintens R, Patel R, Song B, Tsukamoto K, Beullens M, Kaufman RJ, Bollen M, Schuit FC. Glucose activates a protein phosphatase-1-mediated signaling pathway to enhance overall translation in pancreatic beta-cells. Endocrinology. 2007 Feb;148(2):609-17. doi: 10.1210/en.2006-1012.

128. Pavitt GD, Ron D. New insights into translational regulation in the endoplasmic reticulum unfolded protein response. Cold Spring Harb Perspect Biol. 2012 Jun 1;4(6):a012278. doi: 10.1101/cshperspect.a012278.

129. Palam LR, Baird TD, Wek RC. Phosphorylation of eIF2 facilitates ribosomal bypass of an inhibitory upstream ORF to enhance CHOP translation. J Biol Chem. 2011 Apr 1;286(13):10939-49. doi: 10.1074/jbc.M110.216093.
