# Supplementary material for: The Link between Type 2 Diabetes Mellitus and the Polymorphisms of Glutathione-Metabolizing Genes Suggests a New Hypothesis Explaining Disease Initiation and Progression
Source: Life (Basel). 2021 Aug 28;11(9):886. doi: 10.3390/life11090886 (PMC8466482; doi:10.3390/life11090886)
Supplement: Supplementary file 1 [file life-11-00886-s001.zip › Suppl Data S12.pdf]

Supplementary data 12. SNPs linked to GSS and GGT7 gene polymorphisms

The functional effects of *GSS* and *GGT7* gene polymorphisms might be explained by linkage disequilibrium to some other SNP(s) at neighboring genes. A polymorphism rs13041792 is located at 1.4kb 5' of the *GSS* gene and is in a strong linkage disequilibrium (D'≥0.91) to SNPs situated near or in neighboring genes such as *MYH7B* (rs6120772, rs6120775, rs7261969, rs6088667, rs3746446, rs7268266, rs6120788, rs3746436, and rs3746435), *MIR499A* (rs3746444) and *TRPC4AP* (rs752075). Interestingly, certain of these SNPs tightly linked to a polymorphism rs13041792 of the *GSS* gene are associated with the risk of several common diseases. In particular, an SNP rs3746446 at *MYH7B*, a myosin heavy chain 7B, showed a strong association with the risk of congenital heart defects [1]. Numerous studies have reported that SNP rs3746444 of *MIR499A*, a gene encoding microRNA 499a, is associated with the risk of biliary atresia [2], cancer [3,4,5,6], arthritis [7,8], and coronary heart disease [9] as well as diabetic neuropathy in type 2 diabetes [10,11]. Interestingly, Latini with co-workers observed a correlation of allele rs3746444-G at *MIR499A* with a decrease in the number of mtDNA copies in peripheral blood of patients with type 2 diabetes especially those who develop diabetic peripheral neuropathy [12]. A polymorphism rs3746444 in the miR-499 precursor was found to affect the maturation of miR-499-5p, thereby altering its antiapoptotic function [12]. Since polymorphisms rs13041792 of *GSS* and rs3746444 of *MIR499A* are in a strong LD (D'=0.9807) and rs13041792-A allele is correlated with rs3746444-G allele, it can be suggested that the carriage for rs13041792-A may increase susceptibility of the cell to apoptosis. This means that pancreatic β-cells may have an increased sensitivity to apoptosis in the carriers of the rs13041792-A allele of *GSS*. In addition, the DNAse and histone ChIP-Seq data from Roadmap Epigenomics Consortium showed that polymorphisms of the *GSS* gene are involved in epigenetic regulation of transcriptional activity of genes in the pancreatic islets through the changes in chromatin states at enhancer or promoter regions. In particular, a polymorphism rs13041792 at *GSS* along with closely linked SNPs such as rs6088662 (4kb 5' of *GSS*), rs6119548 (9kb 5' of *GSS*), rs6088667 (at *MYH7B*) are located at genomic region regulating gene expression in the pancreatic islets through DNase hypersensitivity site, acetylation at the 9th lysine residue of the histone H3 protein (H3K9ac), methylation at the 4th lysine residue of the histone H3 protein (H3K4me), and chromatin state, respectively.

References

1. Hanchard NA, Swaminathan S, Bucasas K, Furthner D, Fernbach S, Azamian MS, Wang X, Lewin M, Towbin JA, D'Alessandro LC, Morris SA, Dreyer W, Denfield S, Ayres NA, Franklin WJ, Justino H, Lantin-Hermoso MR, Ocampo EC, Santos AB, Parekh D, Moodie D, Jeewa A, Lawrence E, Allen HD, Penny DJ, Fraser CD, Lupski JR, Popoola M, Wadhwa L, Brook JD, Bu'Lock FA, Bhattacharya S, Lalani SR, Zender GA, Fitzgerald-Butt SM, Bowman J, Corsmeier D, White P, Lecerf K, Zapata G, Hernandez P, Goodship JA, Garg V, Keavney BD, Leal SM, Cordell HJ, Belmont JW, McBride KL. A genome-wide association study of congenital cardiovascular left-sided lesions shows association with a locus on chromosome 20. Hum Mol Genet. 2016 Jun 1;25(11):2331-2341. doi: 10.1093/hmg/ddw071.

2. Gawish E, El-Monem EA, El-Abd M, Sobhy GA, Ghanem H. MicroRNA-499 rs3746444 polymorphism in Egyptian children with biliary atresia. Clin Exp Hepatol. 2020 Sep;6(3):263-269. doi: 10.5114/ceh.2020.99526.

3. Tang W, Wang Y, Pan H, Qiu H, Chen S. Association of miRNA-499 rs3746444 A>G variants with adenocarcinoma of esophagogastric junction (AEG) risk and lymph node status. Onco Targets Ther. 2019 Aug 8;12:6245-6252. doi: 10.2147/OTT.S209013.

4. Yang S, Zheng Y, Zhou L, Jin J, Deng Y, Yao J, Yang P, Yao L, Wu Y, Zhai Z, Li N, Lyu L, Dai Z. miR-499 rs3746444 and miR-196a-2 rs11614913 Are Associated with the Risk of Glioma, but Not the Prognosis. Mol Ther Nucleic Acids. 2020 Sep 2;22:340-351. doi: 10.1016/j.omtn.2020.08.038.

5. Tan SC, Lim PY, Fang J, Mokhtar MFM, Hanif EAM, Jamal R. Association between MIR499A rs3746444 polymorphism and breast cancer susceptibility: a meta-analysis. Sci Rep. 2020 Feb 26;10(1):3508. doi: 10.1038/s41598-020-60442-3.

6. Yang X, Li X, Zhou B. A Meta-Analysis of miR-499 rs3746444 Polymorphism for Cancer Risk of Different Systems: Evidence From 65 Case-Control Studies. Front Physiol. 2018 Jun 12;9:737. doi: 10.3389/fphys.2018.00737.

7. Li K, Tie H, Hu N, Chen H, Yin X, Peng C, Wan J, Huang W. Association of two polymorphisms rs2910164 in miRNA-146a and rs3746444 in miRNA-499 with rheumatoid arthritis: a meta-analysis. Hum Immunol. 2014 Jul;75(7):602-8. doi: 10.1016/j.humimm.2014.05.002.

8. Wang D, Pan G. Association of rs2910164 Polymorphism in miRNA-146 and rs3746444 Polymorphism in miRNA-499 with Inflammatory Arthritis: A Meta-Analysis. Biomed Res Int. 2019 May 16;2019:7305750. doi: 10.1155/2019/7305750.

9. Lu JY, Chen MH, Zhang JF, Li ZZ, Liao PH. Association between miR-499 rs3746444 polymorphism and coronary heart disease susceptibility: An evidence-based meta-analysis of 5063 cases and 4603 controls. Gene. 2019 May 25;698:34-40. doi: 10.1016/j.gene.2019.02.045.

10. Ciccacci C, Latini A, Greco C, Politi C, D'Amato C, Lauro D, Novelli G, Borgiani P, Spallone V. Association between a MIR499A polymorphism and diabetic neuropathy in type 2 diabetes. J Diabetes Complications. 2018 Jan;32(1):11-17. doi: 10.1016/j.jdiacomp.2017.10.011.

11. Latini A, Borgiani P, De Benedittis G, D'Amato C, Greco C, Lauro D, Novelli G, Spallone V, Ciccacci C. Mitochondrial DNA Copy Number in Peripheral Blood Is Reduced in Type 2 Diabetes Patients with Polyneuropathy and Associated with a MIR499A Gene Polymorphism. DNA Cell Biol. 2020 Aug;39(8):1467-1472. doi: 10.1089/dna.2019.5326.

12. Ding W, Li M, Sun T, Han D, Guo X, Chen X, Wan Q, Zhang X, Wang J. A polymorphism rs3746444 within the pre-miR-499 alters the maturation of miR-499-5p and its antiapoptotic function. J Cell Mol Med. 2018 Nov;22(11):5418-5428. doi: 10.1111/jcmm.13813.
