# Supplementary material for: The Link between Type 2 Diabetes Mellitus and the Polymorphisms of Glutathione-Metabolizing Genes Suggests a New Hypothesis Explaining Disease Initiation and Progression
Source: Life (Basel). 2021 Aug 28;11(9):886. doi: 10.3390/life11090886 (PMC8466482; doi:10.3390/life11090886)
Supplement: Supplementary file 1 [file life-11-00886-s001.zip › Suppl Data S13.pdf]

Supplementary data 13. Environmental factors responsible for glutathione deficiency.

Type 2 diabetes mellitus is a complex multifactorial disease and its etiology is attributable to numerous risk factors such as genetic, ethnicity, race, poor diet, physical inactivity, and smoking [13,14]. It is widely accepted that nutritional status is one of the major factors influencing the risk of type 2 diabetes [15], and dietary factors play an essential role in maintaining glutathione metabolism. Low consumption of food rich in sulfur-containing amino acids such as cysteine and methionine necessary for the biosynthesis of glutathione is an important factor responsible for endogenous deficiency of glutathione. To prevent this condition, predominantly plant protein-packed foods with glutathione precursors, co-factors, and also whole foods have been shown to boost glutathione stores such as brassica vegetables, fresh fruits, beans, legumes, eggs, seafood, dairy, green tea, and herbs [16], and insufficient intake of such food increases the risk of type 2 diabetes [17,18]. Psychological stress, low physical activity, or sedentary lifestyle are also important factors increasing the risk of type 2 diabetes. Numerous studies [19,20,21,22] have shown that acute and chronic life stresses are associated with a drop of endogenous glutathione. A systematic review and meta-analysis of numerous published studies provided strong evidence for an inverse association between physical activity and the risk of type 2 diabetes [23]. It is suggested that contracting skeletal muscle improves the sensitivity of tissues to insulin action, increases blood flow in the muscle, and enhances glucose uptake into the cells [24,25]. Interestingly, lifelong physical activity was shown to upregulate antioxidant systems, including tissue glutathione homeostasis: regular exercise enhances and chronic inactivity decreases glutathione content in skeletal muscle [26,27,28,29]. Bad habits such as drinking alcohol and tobacco smoking may affect intracellular glutathione content: both tobacco smoking [30,31,32] and alcohol use [33,34,35] were found to deplete GSH pools. Chemical agents of the environment may affect glutathione content in two ways: to deplete the endogenous GSH pool and/or inhibit the biosynthesis of glutathione. The Comparative Toxicogenomics Database (http://ctdbase.org, date of access: 13.10.2020) contains data on 284 chemicals that have been identified to inhibit glutathione biosynthetic processes (GO:0006750) in both humans and laboratory animals (i.e. *Mus musculus* and *Rattus norvegicus*). Five chemical agents such as bisphenol A, diethylhexyl phthalate, hydrogen peroxide, palmitic acid, and streptozocin have the potential to affect the levels of glutathione in the pancreas, and many of these chemicals are well-known risk factors for diabetes [36,37,38,39]. Exposure to agricultural chemicals and industrial pollutants increases the risk of type 2 diabetes [40,41,42,43]. In particular, pesticides such as endosulfan, chlorpyrifos, carbaryl, and organochlorine insecticides [44,45,46] have been associated with the risk of type 2 diabetes, and their causative effects are realized through the depletion of endogenous glutathione stores [47,48,49,50]. There are some examples for glutathione depleting drugs such as Acetaminophen (Tylenol or Paracetamol) [51,52], doxorubicin [51,53], Cyclophosphamide [54], and some other medications [55] that deplete glutathione. Many animal and human studies have reported that the levels of intracellular glutathione progressively declines with aging [56,57] and that the decreased levels of endogenous glutathione have been identified in a variety of chronic diseases such as cardiovascular, respiratory, liver diseases and cancer [58,59,60,61,62,63]. Thus, endogenous deficiency of glutathione can be considered as acquired condition attributable to many factors generally that impact intracellular glutathione content in two ways: to decrease/increase GSH biosynthesis and deplete the endogenous pool of glutathione. Summarizing literature data discussed above it is important to draw a conclusion that almost all environmental factors that affect intracellular glutathione levels are also shared with the development of type 2 diabetes.

Supplementary references

13. Wu Y, Ding Y, Tanaka Y, Zhang W. Risk factors contributing to type 2 diabetes and recent advances in the treatment and prevention. Int J Med Sci. 2014 Sep 6;11(11):1185-200. doi: 10.7150/ijms.10001.

14. Bellou V, Belbasis L, Tzoulaki I, Evangelou E. Risk factors for type 2 diabetes mellitus: An exposure-wide umbrella review of meta-analyses. PLoS One. 2018 Mar 20;13(3):e0194127. doi: 10.1371/journal.pone.0194127.

15. Sami W, Ansari T, Butt NS, Hamid MRA. Effect of diet on type 2 diabetes mellitus: A review. Int J Health Sci (Qassim). 2017 Apr-Jun;11(2):65-71.

16. Minich DM, Brown BI. A Review of Dietary (Phyto)Nutrients for Glutathione Support. Nutrients. 2019 Sep 3;11(9):2073. doi: 10.3390/nu11092073.

17. Tian S, Xu Q, Jiang R, Han T, Sun C, Na L. Dietary Protein Consumption and the Risk of Type 2 Diabetes: A Systematic Review and Meta-Analysis of Cohort Studies. Nutrients. 2017 Sep 6;9(9):982. doi: 10.3390/nu9090982.

18. Zhao WT, Luo Y, Zhang Y, Zhou Y, Zhao TT. High protein diet is of benefit for patients with type 2 diabetes: An updated meta-analysis. Medicine (Baltimore). 2018 Nov;97(46):e13149. doi: 10.1097/MD.0000000000013149.

19. Persky H. Glutathione metabolism in men under psychological stress. Psychosom Med. 1954 Nov-Dec;16(6):489-95. doi: 10.1097/00006842-195411000-00003.

20. Liu J, Mori A. Involvement of reactive oxygen species in emotional stress: A hypothesis based on the immobilization stress-induced oxidative damage and antioxidant defense changes in rat brain, and the effect of antioxidant treatment with reduced glutathione. Int J Stress Manage 1, 249–263 (1994). https://doi.org/10.1007/BF01857992

21. Spiers J. (2014). The Effect of an Acute Psychological Stress on the Reduction-Oxidation Status of Central and Peripheral Tissue. PhD Thesis, School of Biomedical Sciences, The University of Queensland. https://doi.org/10.14264/uql.2014.243

22. Herbet M, Korga A, Gawrońska-Grzywacz M, Izdebska M, Piątkowska-Chmiel I, Poleszak E, Wróbel A, Matysiak W, Jodłowska-Jędrych B, Dudka J. Chronic Variable Stress Is Responsible for Lipid and DNA Oxidative Disorders and Activation of Oxidative Stress Response Genes in the Brain of Rats. Oxid Med Cell Longev. 2017; 2017:7313090. doi: 10.1155/2017/7313090.

23. Aune D, Norat T, Leitzmann M, Tonstad S, Vatten LJ. Physical activity and the risk of type 2 diabetes: a systematic review and dose-response meta-analysis. Eur J Epidemiol. 2015 Jul;30(7):529-42. doi: 10.1007/s10654-015-0056-z.

24. Goodyear LJ, Kahn BB. Exercise, glucose transport, and insulin sensitivity. Annu Rev Med. 1998;49:235-61. doi: 10.1146/annurev.med.49.1.235.

25. Balkau B, Mhamdi L, Oppert JM, Nolan J, Golay A, Porcellati F, Laakso M, Ferrannini E; EGIR-RISC Study Group. Physical activity and insulin sensitivity: the RISC study. Diabetes. 2008 Oct;57(10):2613-8. doi: 10.2337/db07-1605.

26. Sen C.K. (1998) Glutathione: A key role in skeletal muscle metabolism. In: Reznick A.Z., Packer L., Sen C.K., Holloszy J.O., Jackson M.J. (eds) Oxidative Stress in Skeletal Muscle. MCBU Molecular and Cell Biology Updates. Birkhäuser, Basel. https://doi.org/10.1007/978-3-0348-8958-2_8

27. Sen CK. (1999) Glutathione homeostasis in response to exercise training and nutritional supplements. In: Das D.K. (eds) Stress Adaptation, Prophylaxis and Treatment. Molecular and Cellular Biochemistry (An International Journal for Chemical Biology in Health and Disease), vol 32. Springer, Boston, MA. https://doi.org/10.1007/978-1-4615-5097-6_4

28. Francisco Javier O, Manuel R, Manuel RR. Regular physical activity increases glutathione peroxidase activity in adolescents with Down syndrome. Clin J Sport Med. 2006 Jul;16(4):355-6. doi: 10.1097/00042752-200607000-00013.

29. Nyberg M, Mortensen SP, Cabo H, Gomez-Cabrera MC, Viña J, Hellsten Y. Roles of sedentary aging and lifelong physical activity in exchange of glutathione across exercising human skeletal muscle. Free Radic Biol Med. 2014 Aug;73:166-73. doi: 10.1016/j.freeradbiomed.2014.05.008.

30. van der Toorn M, Smit-de Vries MP, Slebos DJ, de Bruin HG, Abello N, van Oosterhout AJ, Bischoff R, Kauffman HF. Cigarette smoke irreversibly modifies glutathione in airway epithelial cells. Am J Physiol Lung Cell Mol Physiol. 2007 Nov;293(5):L1156-62. doi: 10.1152/ajplung.00081.2007.

31. Bazzini C, Rossetti V, Civello DA, Sassone F, Vezzoli V, Persani L, Tiberio L, Lanata L, Bagnasco M, Paulmichl M, Meyer G, Garavaglia ML. Short- and long- term effects of cigarette smoke exposure on glutathione homeostasis in human bronchial epithelial cells. Cell Physiol Biochem. 2013;32(7):129-45. doi: 10.1159/000356633.

32. Altraja S, Mahlapuu R, Soomets U, Altraja A. Cigarette smoke-induced differential regulation of glutathione metabolism in bronchial epithelial cells is balanced by an antioxidant tetrapeptide UPF1. Exp Toxicol Pathol. 2013 Sep;65(6):711-7. doi: 10.1016/j.etp.2012.09.005.

33. Mitchell M.C., Raiford D.S., Mallat A. (1991) Effects of Ethanol on Glutathione Metabolism. In: Watson R.R. (eds) Liver Pathology and Alcohol. Drug and Alcohol Abuse Reviews, vol 2. Humana Press, Totowa, NJ. https://doi.org/10.1007/978-1-4612-0421-3_6

34. Vogt BL, Richie JP Jr. Glutathione depletion and recovery after acute ethanol administration in the aging mouse. Biochem Pharmacol. 2007 May 15;73(10):1613-21. doi: 10.1016/j.bcp.2007.01.033.

35. Waly MI, Kharbanda KK, Deth RC. Ethanol lowers glutathione in rat liver and brain and inhibits methionine synthase in a cobalamin-dependent manner. Alcohol Clin Exp Res. 2011 Feb;35(2):277-83. doi: 10.1111/j.1530-0277.2010.01343.x.

36. Hwang S, Lim JE, Choi Y, Jee SH. Bisphenol A exposure and type 2 diabetes mellitus risk: a meta-analysis. BMC Endocr Disord. 2018 Nov 6;18(1):81. doi: 10.1186/s12902-018-0310-y.

37. Lind PM, Zethelius B, Lind L. Circulating levels of phthalate metabolites are associated with prevalent diabetes in the elderly. Diabetes Care. 2012 Jul;35(7):1519-24. doi: 10.2337/dc11-2396.

38. Qureshi W, Santaren ID, Hanley AJ, Watkins SM, Lorenzo C, Wagenknecht LE. Risk of diabetes associated with fatty acids in the de novo lipogenesis pathway is independent of insulin sensitivity and response: the Insulin Resistance Atherosclerosis Study (IRAS). BMJ Open Diabetes Res Care. 2019 Sep 4;7(1):e000691. doi: 10.1136/bmjdrc-2019-000691.

39. Damasceno DC, Netto AO, Iessi IL, Gallego FQ, Corvino SB, Dallaqua B, Sinzato YK, Bueno A, Calderon IM, Rudge MV. Streptozotocin-induced diabetes models: pathophysiological mechanisms and fetal outcomes. Biomed Res Int. 2014;2014:819065. doi: 10.1155/2014/819065.

40. Taylor KW, Novak RF, Anderson HA, Birnbaum LS, Blystone C, Devito M, Jacobs D, Köhrle J, Lee DH, Rylander L, Rignell-Hydbom A, Tornero-Velez R, Turyk ME, Boyles AL, Thayer KA, Lind L. Evaluation of the association between persistent organic pollutants (POPs) and diabetes in epidemiological studies: a national toxicology program workshop review. Environ Health Perspect. 2013 Jul;121(7):774-83. doi: 10.1289/ehp.1205502.

41. Evangelou E, Ntritsos G, Chondrogiorgi M, Kavvoura FK, Hernández AF, Ntzani EE, Tzoulaki I. Exposure to pesticides and diabetes: A systematic review and meta-analysis. Environ Int. 2016 May;91:60-8. doi: 10.1016/j.envint.2016.02.013.

42. Meo SA, AlMutairi FJ, Alasbali MM, Alqahtani TB, AlMutairi SS, Albuhayjan RA, Al Rouq F, Ahmed N. Men's Health in Industries: Plastic Plant Pollution and Prevalence of Pre-diabetes and Type 2 Diabetes Mellitus. Am J Mens Health. 2018 Nov;12(6):2167-2172. doi: 10.1177/1557988318800203.

43. Bowe B, Xie Y, Li T, Yan Y, Xian H, Al-Aly Z. The 2016 global and national burden of diabetes mellitus attributable to PM2.5 air pollution. Lancet Planet Health 2018; 2: e301–312. doi: 10.1016/S2542-5196(18)30140-2.

44. Juntarawijit C, Juntarawijit Y. Association between diabetes and pesticides: a case-control study among Thai farmers. Environ Health Prev Med. 2018 Jan 27;23(1):3. doi: 10.1186/s12199-018-0692-5.

45. Son HK, Kim SA, Kang JH, Chang YS, Park SK, Lee SK, Jacobs DR Jr, Lee DH. Strong associations between low-dose organochlorine pesticides and type 2 diabetes in Korea. Environ Int. 2010 Jul;36(5):410-4. doi: 10.1016/j.envint.2010.02.012.

46. Kuo CC, Moon K, Thayer KA, Navas-Acien A. Environmental chemicals and type 2 diabetes: an updated systematic review of the epidemiologic evidence. Curr Diab Rep. 2013 Dec;13(6):831-49. doi: 10.1007/s11892-013-0432-6.

47. Ahmed T, Tripathi AK, Ahmed RS, Das S, Suke SG, Pathak R, Chakraboti A, Banerjee BD. Endosulfan-induced apoptosis and glutathione depletion in human peripheral blood mononuclear cells: Attenuation by N-acetylcysteine. J Biochem Mol Toxicol. 2008 Sep-Oct;22(5):299-304. doi: 10.1002/jbt.20240.

48. Bebe FN, Panemangalore M. Exposure to low doses of endosulfan and chlorpyrifos modifies endogenous antioxidants in tissues of rats. J Environ Sci Health B. 2003 May;38(3):349-63. doi: 10.1081/PFC-120019901.

49. Lechner DW, Abdel-Rahman MS. The effect of carbaryl and malathion in combination on gamma-glutamyl transpeptidase and glutathione S-alkyltransferase activity in vitro. Arch Environ Contam Toxicol. 1986 Nov;15(6):647-51. doi: 10.1007/BF01054911.

50. Dehn PF, Allen-Mocherie S, Karek J, Thenappan A. Organochlorine insecticides: impacts on human HepG2 cytochrome P4501A, 2B activities and glutathione levels. Toxicol In Vitro. 2005 Mar;19(2):261-73. doi: 10.1016/j.tiv.2004.10.002.

51. Wells PG, Boerth RC, Oates JA, Harbison RD. Toxicologic enhancement by a combination of drugs which deplete hepatic glutathione: acetaminophen and doxorubicin (adriamycin). Toxicol Appl Pharmacol. 1980 Jun 30;54(2):197-209. doi: 10.1016/0041-008x(80)90187-8.

52. Dimova S, Hoet PH, Dinsdale D, Nemery B. Acetaminophen decreases intracellular glutathione levels and modulates cytokine production in human alveolar macrophages and type II pneumocytes in vitro. Int J Biochem Cell Biol. 2005 Aug;37(8):1727-37. doi: 10.1016/j.biocel.2005.03.005.

53. Paranka NS, Dorr RT. Effect of doxorubicin on glutathione and glutathione-dependent enzymes in cultured rat heart cells. Anticancer Res. 1994 Sep-Oct;14(5A):2047-52.

54. DeLeve LD. Cellular target of cyclophosphamide toxicity in the murine liver: role of glutathione and site of metabolic activation. Hepatology. 1996 Oct;24(4):830-7. doi: 10.1002/hep.510240414.

55. Moine L, Rivoira M, Díaz de Barboza G, Pérez A, Tolosa de Talamoni N. Glutathione depleting drugs, antioxidants and intestinal calcium absorption. World J Gastroenterol. 2018 Nov 28;24(44):4979-4988. doi: 10.3748/wjg.v24.i44.4979.

56. Erden-Inal M, Sunal E, Kanbak G. Age-related changes in the glutathione redox system. Cell Biochem Funct. 2002 Mar;20(1):61-6. doi: 10.1002/cbf.937.

57. Maher P. The effects of stress and aging on glutathione metabolism. Ageing Res Rev. 2005 May;4(2):288-314. doi: 10.1016/j.arr.2005.02.005.

58. Cavalca V, Veglia F, Squellerio I, Marenzi G, Minardi F, De Metrio M, Cighetti G, Boccotti L, Ravagnani P, Tremoli E. Glutathione, vitamin E and oxidative stress in coronary artery disease: relevance of age and gender. Eur J Clin Invest. 2009 Apr;39(4):267-72. doi: 10.1111/j.1365-2362.2009.02094.x.

59. Shimizu H, Kiyohara Y, Kato I, Kitazono T, Tanizaki Y, Kubo M, Ueno H, Ibayashi S, Fujishima M, Iida M. Relationship between plasma glutathione levels and cardiovascular disease in a defined population: the Hisayama study. Stroke. 2004 Sep;35(9):2072-7. doi: 10.1161/01.STR.0000138022.86509.2d.

60. Rahman I, MacNee W. Lung glutathione and oxidative stress: implications in cigarette smoke-induced airway disease. Am J Physiol. 1999 Dec;277(6):L1067-88. doi: 10.1152/ajplung.1999.277.6.L1067.

61. Fitzpatrick AM, Teague WG, Holguin F, Yeh M, Brown LA; Severe Asthma Research Program. Airway glutathione homeostasis is altered in children with severe asthma: evidence for oxidant stress. J Allergy Clin Immunol. 2009 Jan;123(1):146-152.e8. doi: 10.1016/j.jaci.2008.10.047.

62. Shaw S, Rubin KP, Lieber CS. Depressed hepatic glutathione and increased diene conjugates in alcoholic liver disease. Evidence of lipid peroxidation. Dig Dis Sci. 1983 Jul;28(7):585-9. doi: 10.1007/BF01299917.

63. Balendiran GK, Dabur R, Fraser D. The role of glutathione in cancer. Cell Biochem Funct. 2004 Nov-Dec;22(6):343-52. doi: 10.1002/cbf.1149.
