# Supplementary material for: The Link between Type 2 Diabetes Mellitus and the Polymorphisms of Glutathione-Metabolizing Genes Suggests a New Hypothesis Explaining Disease Initiation and Progression
Source: Life (Basel). 2021 Aug 28;11(9):886. doi: 10.3390/life11090886 (PMC8466482; doi:10.3390/life11090886)
Supplement: Supplementary file 1 [file life-11-00886-s001.zip › Suppl Table S1.pdf]

Supplementary Table S1. Linkage disequilibrium between SNPs in the Russian population

Gene / SNP ID		*GSS* rs6088660	*GSS* rs13041792	*GGT7* rs6119534	*GGT7* rs11546155
GSS	rs1801310	**-0.1086**	**-0.0714**	**-0.1402**	**-0.0331**
		**0.9802**	**0.8986**	**0.8333**	**0.8165**
	rs6088660		**-0.0557**	**0.0615**	**0.0449**
			**0.9828**	**0.3813**	**0.6067**
	rs13041792			**0.0922**	**-0.0187**
				**0.7978**	**0.9014**
GGT7	rs6119534				**-0.0361**
					**0.8228**

Upper cells show *D*-values (gray fill); lower cells show *D*′-values ($P<2.0\times10^{-16}$).
