# Supplementary material for: The Link between Type 2 Diabetes Mellitus and the Polymorphisms of Glutathione-Metabolizing Genes Suggests a New Hypothesis Explaining Disease Initiation and Progression
Source: Life (Basel). 2021 Aug 28;11(9):886. doi: 10.3390/life11090886 (PMC8466482; doi:10.3390/life11090886)
Supplement: Supplementary file 1 [file life-11-00886-s001.zip › Suppl Table S2.pdf]

life

Supplementary Table S2. Linkage disequilibrium values (*D′*) between pairs of the studied SNPs across populations from the 1000 Genomes Project

Gene / SNP ID		MAF	Population	*GSS*		*GGT7*	
				rs6088660	rs13041792	rs6119534	rs11546155
GSS	rs1801310	0.394	Russian	0.9802	0.8986	0.8333	0.8165
		0.067	AFR	0.4512	0.9382	0.9471	1.0000
		0.599	AMR	0.9831	0.9660	0.9896	0.9549
		0.564	EAS	0.9839	0.9747	0.9873	0.4751
		0.428	EUR	1.0000	0.9770	0.9816	0.8487
		0.238	SAS	0.9873	0.9678	0.9877	0.8487
	rs6088660	0.281	Russian	*	0.9828	0.3813	0.6067
		0.166	AFR	*	1.0000	0.3411	0.3608
		0.143	AMR	*	1.0000	0.5012	0.6217
		0.218	EAS	*	1.0000	0.9018	0.5499
		0.258	EUR	*	1.0000	0.3201	0.6629
		0.337	SAS	*	1.0000	0.8170	0.7098
	rs13041792	0.202	Russian		*	0.7978	0.9014
		0.184	AFR		*	0.5859	1.0000
		0.141	AMR		*	0.8409	0.4258
		0.139	EAS		*	0.9502	0.8000
		0.202	EUR		*	0.9050	0.7247
		0.267	SAS		*	0.9236	0.9712
GGT7	rs6119534	0.428	Russian			*	0.8228
		0.215	AFR			*	0.9741
		0.231	AMR			*	0.8828
		0.139	EAS			*	0.9333
		0.378	EUR			*	0.9755
		0.348	SAS			*	0.9889
GGT7	rs11546155	0.103	Russian				*
		0.136	AFR				*
		0.053	AMR				*
		0.107	EAS				*
		0.107	EUR				*
		0.266	SAS				*

D′-values estimated by LDpair Tool (https://ldlink.nci.nih.gov/?tab=ldpair). Only statistically significant *D′*-values are shown.
* Populations: Russian (the studied population); AFR, African; AMR, Ad Mixed American; EAS, East Asian; EUR, European; SAS, South Asian. MAF, minor allele frequency.
